# Dataset of bond enthalpies (ε_AA_, ε_AB_, ε_BB_) in 975 binary intermetallic compounds

**DOI:** 10.1016/j.dib.2021.107652

**Published:** 2021-11-28

**Authors:** Daniel Miracle, Amanda Dahlman, Garth Wilks, James E. Dahlman

**Affiliations:** a AF Research Laboratory, Materials and Manufacturing Directorate, Wright-Patterson AFB, OH USA; b Independent Sales Director, Dayton, OH USA

**Keywords:** Thermodynamics, bond strength, metals and alloys, regular solution model, chemical bond strength, coordination number

## Abstract

The concept of a bond energy between a pair of atoms is well-established in chemistry and physics. In materials science, it is a fundamental parameter in the development of thermodynamic models such as the regular, quasi-chemical and sub-regular solution models, as well as the central atoms model. Accurate bond dissociation enthalpies are available for gaseous molecular compounds, but these values are likely to differ significantly from single bond strengths between atoms in liquids and solids. While interatomic potential functions have been developed for atomic pairs by fitting to observed quantities, these functions often contain invariant transformations that yield bond energies that differ by up to a factor of four from values provided by other potentials for the same system, even though both may produce the same physical properties. Moreover, there is presently no widely used approach to determine bond enthalpies in condensed phases. An approach has been developed earlier to calculate bond enthalpies in solid and liquid phases using classical thermodynamic concepts, measured enthalpies for compound formation and elemental sublimation, and by counting bonds in the thermodynamically stable product and reactant phases (Miracle et al., 2011). That work reported bond enthalpies for essentially all stable solid and liquid elements, as well as bond strengths between unlike atoms for 71 different intermetallic compounds in 15 binary systems. This earlier work was validated by estimating elemental fusion enthalpies and surface energies, as well as formation enthalpies for ternary intermetallic compounds. Given the utility of this approach, the present dataset applies this earlier methodology to produce bond enthalpies between unlike atom pairs in an additional 904 binary intermetallic compounds from 443 systems, giving a total dataset of 975 bond enthalpies from 458 binary systems. Typical errors in the values reported here (from enthalpy measurements) are ±4%, and larger errors of about ±10-30% occur for a small subset of values where the number of bonds in the structure are difficult to establish. Used appropriately, these bond enthalpies enable classical approximations that—to first order—can capture critical material properties. A wide range of such estimates are possible, including the energies of vacancies and other atomic defects, solution enthalpies for complex, concentrated solid solution alloys (CCAs), formation enthalpies of higher-order compounds, and metallic glass stability. These bond enthalpies may also be useful for establishing trends in systematic studies that cover many systems, thus narrowing the scope of subsequent experimental measurements or computations which are more accurate, but are also more difficult and time consuming.

## Specifications Table

**Table:** 

Subject	Materials Science
Specific subject area	Bond strengths between atom pairs in solids and liquids
Type of data	Table Graph
How data were acquired	The data provided here were calculated using binary compound formation enthalpies, elemental sublimation enthalpies (both compiled from published sources), and data available in crystallographic handbooks.
Data format	Raw Analyzed
Description of data collection	Elemental sublimation and compound formation enthalpies were collected from the literature. These values are primarily experimental and/or assessed values at standard temperature and pressure (298 K, 1 atm pressure). Published crystallographic data were used to determine the number of bonds in product and reactant phases for atom pairs separated by up to 125% of the minimum separation distances. The primary data sources for these initial inputs are provided below. These input data were then analyzed and used to produce the current raw dataset of bond counts and bond enthalpies.
Data source location	Primary data sources: See references [2], [3], [4], [5], [6], [7], [8], [9], [10], [11], [12], [13], [14], [15], [16], [17] at the end of the article.
Data accessibility	Repository name: Mendeley Data Data identification number: 10.17632/jycyj6dxyy.1 Direct URL to data: https://data.mendeley.com/datasets/jycyj6dxyy/1
Related research article	D.B. Miracle, G.B. Wilks, A.G. Dahlman and J.E. Dahlman, The strength of chemical bonds in solids and liquids, Acta Mater. 59 (2011) 7840-7854. https://doi.org/10.1016/j.actamat.2011.09.003

## Value of the Data

• The energy of a chemical bond between atoms in solids and liquids is a fundamental quantity that can be used to study a wide range of problems in materials science.
• This dataset can be used by researchers who require fundamental quantities to explore basic features and processes in materials science that range from the atomic scale to bulk behaviours.
• This dataset may be useful for classical approximations supporting initial screening studies, obtaining estimates where more accurate measurements or calculations are not feasible or available, exploring trends across diverse classes of alloy systems or structures, and discovering new insights into fundamental processes.

## 1 Data Description

Thermodynamic and crystallographic raw data are required as inputs to produce the bond enthalpies in the present dataset. Sublimation enthalpies for pure elements ($\Delta_{f}H\left(i,gas\right)$) and formation enthalpies for binary compounds ($\Delta_{f}H\left(A_{x}B_{y}\right)$) are taken from [2], [3], [4], [5], [6], [7], [8], [9],[11], [12], [13], [14], [15], [16], [17]. Crystallographic data used to count the number of A–A, A–B and B–B bonds in the equilibrium structures of the elements and compounds considered here are provided in [10]. The thermodynamic and crystallographic data taken from these sources, along with the condensed bond enthalpies derived from these raw data in the present work, are presented in Table S1. The first column lists either the element or the binary compound of interest. Elements are given first and are listed in order of atomic number. The compounds are listed in alphabetic order, and within a given binary system they are further sorted by increasing atom fraction of element *B* (defined below). The second column gives the Pearson symbol and the prototype element or compound for the structure of each condensed phase. The third and fourth columns list element *A* and *B*, respectively, in the binary compound *A_x_B_y_*. Element *A* and *B* are chosen so that the condensed bond enthalpy for an *A—A* bond in pure element *A*, $\varepsilon_{AA}^{A}$, is less negative than the condensed bond enthalpy for a *B—B* bond in pure element *B*, $\varepsilon_{BB}^{B}$. Element *B* generally has a higher melting temperature than element *A*. The fifth column gives either the enthalpy required to convert a pure element at standard temperature and pressure (298 K, 1 atm) to the vapor state (also called the sublimation enthalpy), or the formation enthalpy needed to produce the compound *A_x_B_y_* from *x* moles of *A* and *y* moles of *B* at standard temperature and pressure. The formation enthalpies are given per mole of compound. The sixth column gives the error in column five values. These are either the experimentally reported errors or, where no error is reported, this is the average of the reported errors within this dataset. Column seven gives the atom fraction of element *B*. Columns 8-10 list the numbers of *A—A, A*—*B* and *B—B* bonds per unit cell for the thermodynamically stable crystal structure adopted by the compound of interest at standard temperature and pressure. The method for counting bonds is given in [1], and additional details are given in Experimental Design, Materials and Methods regarding a correction for relative atom size. In the case that ambiguity exists in the number of bonds due to uncertainties in atom types in the published atom separation histograms [10], the average of the minimum and maximum values is used and the resulting uncertainty is included in the errors shown in columns 14, 16, 18 and 20. Columns 11, 12 give the average number of bonds formed by *A* and *B* atoms, respectively in the same thermodynamically stable crystal structures. These values are half the coordination numbers of *A* and *B* atoms, regardless of whether the bonds are formed between like or unlike atoms. Column 13 gives the condensed bond enthalpy (CBE) for *A—A* bonds in pure element *A* ($\varepsilon_{AA}^{A}$) or in compound *A_x_B_y_* ($\varepsilon_{AA}^{AxBy}$), and Column 14 lists the error in these values by accounting for the error reported in column six and, when present, in the uncertainties in the number of bonds per unit cell given in columns 8–10. Columns 15, 16 give the CBE for *B—B* bonds in compound *A_x_B_y_* ($\varepsilon_{BB}^{AxBy}$) and its error, respectively. Columns 17, 18 give the magnitude of the CBEs for *A—B* bonds in *A_x_B_y_* ($\varepsilon_{AB}$) and its error. The term condensed bond enthalpy (CBE) is used to avoid confusion with the well-established term, bond enthalpy (BE), defined by the International Union of Pure and Applied Chemistry (IUPAC) as the average value of the bond dissociation enthalpy (BDE) for all bonds of the same type within the same gaseous molecules.

A reference, or standard, thermodynamic state is needed to quantify the reaction enthalpies in column 5 and to derive the bond enthalpies in Table S1. We use the gas standard state as the reference state. In simple terms, this means that the enthalpy contained in *A—A, A—B* and *B—B* bonds is taken to be zero in the gas state, where the atoms are at essentially infinite separations. This is consistent with the approach typically used in the physics community for showing interatomic potentials, where the energy at infinite atomic separations is zero. The values of $\varepsilon_{AA}^{A}$, $\varepsilon_{AA}^{AxBy}$, $\varepsilon_{BB}^{AxBy}$ and $\varepsilon_{AB}$ in columns 13, 15 and 17 thus represent the depth of the interatomic well at the equilibrium atom separations. The materials thermodynamics community often uses a ‘metal’ standard state, where the enthalpy of an *A—A* bond in pure element *A* and a *B—B* bond in pure element *B* are both set to zero and the bond enthalpies between unlike atoms, $\varepsilon_{AB,metal}$, are given relative to these values – these are given in column 19 along with the error in column 20. These values are likely to have less utility than the values derived from the gas standard state but are given for completeness.

A digital form of the data contained in Table S1, along with detailed implementation of the methodology described in [1] and contained in the present work, is given in an Excel worksheet (https://data.mendeley.com/datasets/jycyj6dxyy/1). This Excel worksheet has five tabs. The first tab, e_AA, provides the input values and calculated results for the condensed bond enthalpies between like atoms in the pure, elemental state ($\varepsilon_{AA}^{A}$). The second tab, dH_assess, gives the raw, input data used to assess the formation enthalpies of the binary compounds, *A_x_B_y_*, that are thermodynamically stable at standard temperature and pressure. The third tab, pij_assess, gives the input data and details of the calculations used to determine the number of A–A, A–B and B–B bonds in each of the 240 distinct crystal structures represented in the current dataset. The fourth tab, e_AB, gives the principal output data included in Table S1 of this report, along with the intermediary results needed to produce these computed values. Finally, the fifth tab, Variables, gives a detailed listing and description for each of the Excel variables used in the preceding four tabs of the worksheet. Throughout this worksheet, values shown in blue font represent raw, input values and values shown in black font indicate calculated values.

The present approach is a simplification expected to provide general utility for classical approximations and for use in identifying trends across groups of systems. The enthalpies of actual bonds in structures may be more complex and nuanced. For example, bond enthalpies for a given bond type within a given structure may vary slightly due to small differences in atom separations at non-equivalent Wyckoff positions, and the occurrence of bond hybridization may alter the balance between like and unlike bond enthalpies. Such refinements are not included in the present work and may be considered elsewhere.

**Fig. 1:**
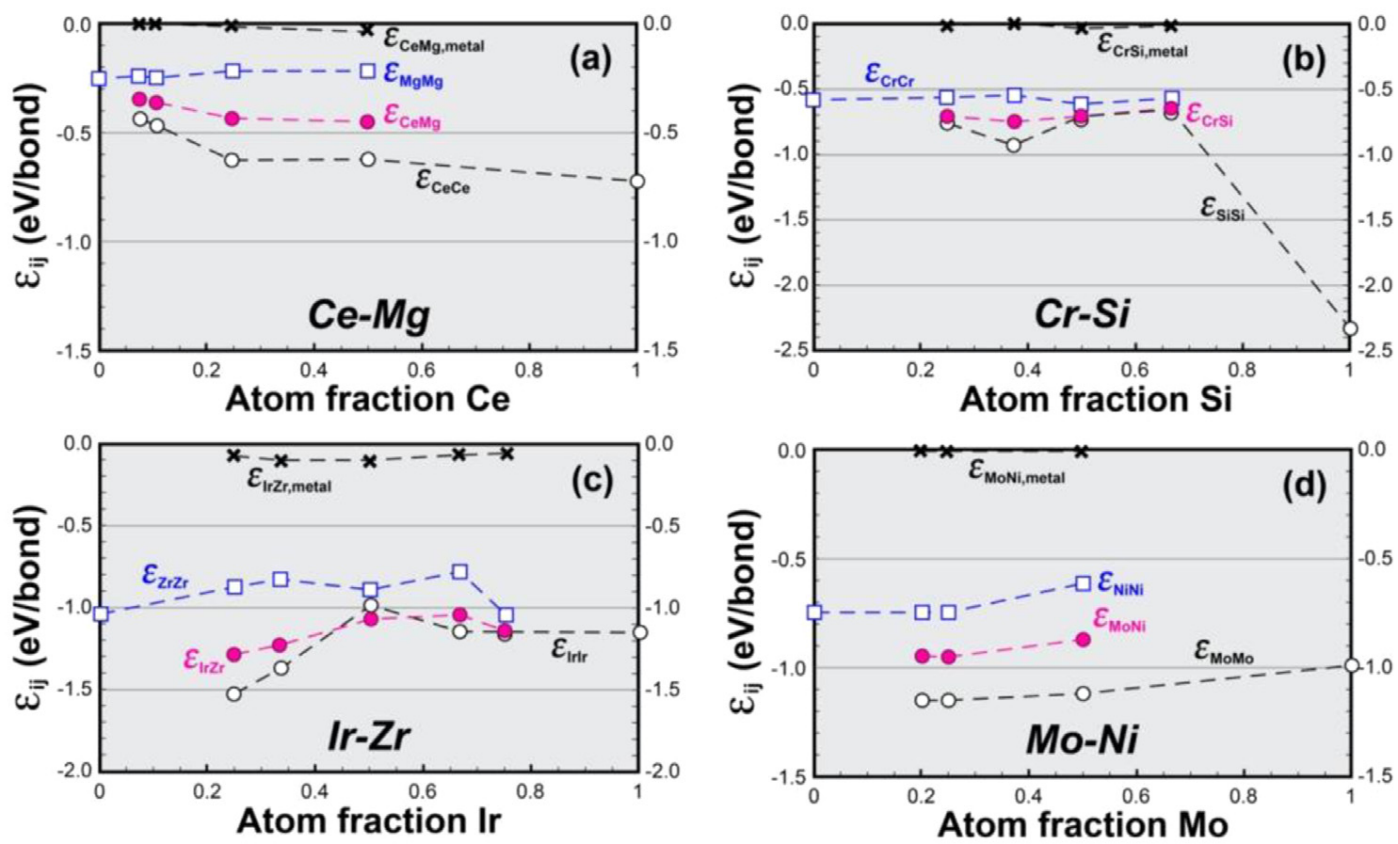
Plots of condensed bond enthalpies, $\varepsilon_{ij}$, for (a) the Ce-Mg system, (b) the Cr-Si system, (c) the Ir-Zr system and (d) the Mo-Ni system. $\varepsilon_{AA}$ is shown by open squares () and $\varepsilon_{BB}$ by open circles (○). $\varepsilon_{AB}$ determined from the gas standard state is shown as closed circles (), and $\varepsilon_{AB,metal}$ from the metal standard state as (×). The horizontal axis gives the atom fraction of element *B*.

Bond enthalpies for four binary systems from Table S1 are plotted in Fig. 1(a–d) to illustrate general features exhibited by binary systems within this dataset. Values for $\varepsilon_{AA}$ extend from atom fraction *B*, f*_B_* = 0 (for pure element *A*, $\varepsilon_{AA}^{A}$) to 0 < f*_B_* < 1 for compounds *A_x_B_y_* ($\varepsilon_{AA}^{AxBy}$). Conversely, $\varepsilon_{BB}$ values start at f*_B_* = 1 (for pure element *B*, $\varepsilon_{BB}^{B}$) and can occur for any f*_B_* > 0 for compounds *A_x_B_y_* ($\varepsilon_{BB}^{AxBy}$). $\varepsilon_{AB}$ values occur within the range 0 < f*_B_* < 1. The composition is given by the compound stoichiometry *A_x_B_y_*, since f*_B_* = y/(x+y). The difference between $\varepsilon_{AA}^{A}$ and $\varepsilon_{BB}^{B}$ can be small, as in Fig. 1(c), or it can be more significant. For example, Fig. 1(b) shows an extreme case, where $\varepsilon_{SiSi}^{Si}$ in pure Si (atom fraction Si = 1) is much more negative than $\varepsilon_{CrCr}^{Cr}$ in pure Cr (atom fraction Si = 0). The bond strength between like atoms in the pure element can differ from the same bond in compounds when the number of bonds formed per atom is different in the elemental and compound structures. These differences are usually modest, but can be large when an atom is sparsely bonded in either the element or the compound. This is illustrated in Fig. 1(b), where elemental Si has a coordination number of 4 but forms many more bonds in Cr-Si compounds. In this case, $\varepsilon_{BB}^{B}$ is much more negative than $\varepsilon_{BB}^{AxBy}$. Differences can occur in either direction, for example $\varepsilon_{BB}^{B}$ < $\varepsilon_{BB}^{AxBy}$ in Fig. 1(a,b) but the opposite trend is shown in Fig. 1(d). In Fig. 1(c), $\varepsilon_{BB}^{AxBy}$ values are both more negative and less negative than $\varepsilon_{BB}^{B}$. Similar differences are found between $\varepsilon_{AA}^{A}$ and $\varepsilon_{AA}^{AxBy}$ values. $\varepsilon_{AB}$ usually occurs between $\varepsilon_{AA}^{AxBy}$ and $\varepsilon_{BB}^{AxBy}$, but in unusual cases can be more negative than both of these values as shown in Fig. 1(c) at f*_B_* = 0.5. The bond enthalpy determined using the metal standard state, $\varepsilon_{AB,metal}$, is usually negative and very close to zero. $\varepsilon_{AB,metal}$ is essentially the deviation in the unlike bond enthalpy relative to the compositionally weighted average of $\varepsilon_{AA}^{AxBy}$ and $\varepsilon_{BB}^{AxBy}$, and since $\varepsilon_{AA}^{AxBy}=\varepsilon_{BB}^{AxBy}=0$ in the metal standard state, this shows that the bond energies between unlike atoms are slightly more negative than the weighted average between like-atom bonds.

## 2 Experimental Design, Materials and Methods

We start by calculating the bond enthalpy between like atoms in a condensed (liquid or solid) elemental phase. The enthalpy needed to convert one mole of pure, condensed element *A* at standard temperature and pressure to a non-interacting gas, $\Delta_{f}H\left(A,gas\right)$, has been measured experimentally as the heat of formation of a monatomic gas, also called the heat of sublimation. This is the enthalpy difference between the final and initial states,

(1) $$\Delta_{f}H\left(A,gas\right)=H\left(A,gas\right)-H\left(A,cond\right)$$

We choose the non-interacting gaseous atoms as the thermodynamic reference state, so that H(A,gas)=0. Further, we apply the classical thermodynamic concept that underlies the regular solution and quasi-chemical models – that the enthalpy of a condensed phase, *H*, can be represented as the sum of the bond enthalpies of the first atom neighbors.

(2) $$H=\sum_{i,j}\varepsilon_{ij}P_{ij}$$

where $P_{ij}$ is the number of *A—A, A—B* and *B—B* bonds per mole of condensed phase. Thus, the enthalpy of condensed element *A* is equal to the product of the enthalpy contained in an *A—A* bond, $\varepsilon_{AA}^{A}$, and the number of bonds in a mole of condensed element *A* in its equilibrium structure at standard temperature and pressure. This latter term is given by the coordination number, *Z_AA_*, times Avogadro's number, *N_Av_*, divided by two to avoid double-counting bonds. Inserting these in Eq. (1) gives

(3a) $$\Delta_{f}H\left(A,gas\right)=-H\left(A,cond\right)=-\varepsilon_{AA}^{A}\left(N_{Av}Z_{AA}\right)/2$$

and rearranging terms gives the result

(3b) $$\varepsilon_{AA}^{A}=-2\Delta_{f}H\left(A,gas\right)/\left(N_{Av}Z_{AA}\right)$$

The heat of sublimation is a positive quantity, so the bond enthalpy is a negative value. The values of $\varepsilon_{AA}^{A}$ are calculated using tabulated values of $\Delta_{f}H\left(A,gas\right)$ [2,^8^,^16^,17]. As calculated, $\varepsilon_{AA}^{A}$ has units of joules per bond, these are converted to electron-volts (eV) per bond for convenience. The values thus obtained are shown in Table S1 and have been reported in an earlier publication [1].

To calculate the enthalpy contained in bonds between unlike atoms, consider the reaction between *x* moles of element *A* with *y* moles of element *B* to form one mole of the equilibrium compound, *A_x_B_y_* at standard temperature and pressure. The formation enthalpy, $\Delta_{f}H\left(A_{x}B_{y}\right)$, can be measured experimentally, and represents the difference between the enthalpies of the products and reactants

(4) $$\Delta_{f}H\left(A_{x}B_{y}\right)=H\left(A_{x}B_{y}\right)-xH\left(A,cond\right)-yH\left(B,cond\right)$$

$H(A_{x}B_{y})$ is expanded using Eq. (2) to give

(5) $$H\left(A_{x}B_{y}\right)=\varepsilon_{AA}^{AxBy}P_{AA}^{AxBy}+\varepsilon_{AB}^{AxBy}P_{AB}^{AxBy}+\varepsilon_{BB}^{AxBy}P_{BB}^{AxBy}$$

$P_{ij}^{AxBy}$ is the number of *i*—*j* bonds per mole of *A_x_B_y_* and $\varepsilon_{ij}^{AxBy}$ is the enthalpy of an *i*—*j* bond in the compound *A_x_B_y_*. As described later, the enthalpies of *A—A* and *B—B* bonds can be different in the compound than in the pure element, and so the terms $\varepsilon_{AA}^{AxBy}$ and $\varepsilon_{BB}^{AxBy}$ are used to distinguish them from $\varepsilon_{AA}^{A}$ and $\varepsilon_{BB}^{B}$. $\varepsilon_{AB}^{AxBy}$ occurs only in the compound, and so the superscript is implied and is generally omitted for simplicity. Substituting results for H(i,cond) from Eq. (3a) and for $H(A_{x}B_{y})$ from Eq. (5) into Eq. (4) gives

(6) $$\Delta_{f}H\left(A_{x}B_{y}\right)=\varepsilon_{AA}^{AxBy}P_{AA}^{AxBy}+\varepsilon_{AB}P_{AB}^{AxBy}+\varepsilon_{BB}^{AxBy}P_{BB}^{AxBy}+x\Delta_{f}H\left(A,gas\right)+y\Delta_{f}H\left(B,gas\right)$$

Rearranging terms to solve for $\varepsilon_{AB}$ gives

(7) $$\varepsilon_{AB}=\left(1/P_{AB}^{AxBy}\right)\left[\Delta_{f}H\left(A_{x}B_{y}\right)-x\Delta_{f}H\left(A,gas\right)-y\Delta_{f}H\left(B,gas\right)-\varepsilon_{AA}^{AxBy}P_{AA}^{AxBy}-\varepsilon_{BB}^{AxBy}P_{BB}^{AxBy}\right]$$

The values of $P_{ij}^{AxBy}$ can be determined from published data [10] using the following relation,

(8) $$P_{ij}^{AxBy}=p_{ij}^{AxBy}\left[N_{Av}\left(x+y\right)/U\right]$$

where *U* is the number of atoms per unit cell (included in the Pearson symbol) and $p_{ij}^{AxBy}$ is the number of *i*—*j* bonds per *A_x_B_y_* unit cell. The bonds are counted for the particular structure of each compound that is thermodynamically stable at standard temperature and pressure. The method for counting bonds is described in [1]. Substituting this into Eq. (7) gives

(9) $$\begin{array}{ccc} \varepsilon_{AB} & = & \left[U/\left(p_{AB}^{AxBy}N_{Av}\left(x+y\right)\right)\right]\{\Delta_{f}H\left(A_{x}B_{y}\right)-x\Delta_{f}H\left(A,gas\right)-y\Delta_{f}H\left(B,gas\right)\\ & & -\;\varepsilon_{AA}^{AxBy}\left[\left(p_{AA}^{AxBy}N_{Av}\left(x+y\right)\right)/U\right]-\varepsilon_{BB}^{AxBy}\left[\left(p_{BB}^{AxBy}N_{Av}\left(x+y\right)\right)/U\right]\} \end{array}$$

and simplifying yields

(10) $$\begin{array}{ccc} \varepsilon_{AB} & = & \left\{1/\left(p_{AB}^{AxBy}\right)\right\}\{\left(U/N_{Av}\left(x+y\right)\right)\left[\Delta_{f}H\left(A_{x}B_{y}\right)-x\Delta_{f}H\left(A,gas\right)-y\Delta_{f}H\left(B,gas\right)\right]-\varepsilon_{AA}^{AxBy}p_{AA}^{AxBy}\\ & & -\;\varepsilon_{BB}^{AxBy}p_{BB}^{AxBy}\} \end{array}$$

Finally, the enthalpy of an *i*—*i* bond in a compound can be different than in pure element *i* due to chemical and structural differences between the element and the compound. An adjustment for structural effects is made following Pauling's rule for ionic bonding, which states that the bond strength depends directly on the number of bonds formed [18]. This suggests that an atomic species has a fixed capacity to form bonds, and the more bonds formed by an atom, the less will be the energy per bond. Though developed for ionically bonded compounds, this may have relevance for compounds that are characterized by a blend of metallic and covalent bonding as well. As a result,

(11) $$\varepsilon_{ii}^{AxBy}=\left({\bar{P}}_{i}^{i}/{\bar{P}}_{i}^{AxBy}\right)\varepsilon_{ii}^{i}$$

${\bar{P}}_{i}^{i}$ is the number of bonds formed by *i* atoms in pure element *i* and ${\bar{P}}_{i}^{AxBy}$ is the average number of bonds formed by *i* atoms in the compound *A_x_B_y_*. ${\bar{P}}_{i}^{i}$ consists only of bonds between like atoms, while ${\bar{P}}_{i}^{AxBy}$ includes both *i*—*i* and *i*—*j* bonds. The final equation for the enthalpy of *A*—*B* bonds from Eq. (10) is thus

(12) $$\begin{array}{ccc} \varepsilon_{AB} & = & \left(\frac{1}{p_{AB}^{AxBy}}\right)\{\left(\frac{U}{N_{Av}\left(x+y\right)}\right)\left[\Delta_{f}H\left(A_{x}B_{y}\right)-x\Delta_{f}H\left(A,gas\right)-y\Delta_{f}H\left(B,gas\right)\right]\\ & & -\;\varepsilon_{AA}^{A}p_{AA}^{AxBy}\left({\bar{P}}_{A}^{A}/{\bar{P}}_{A}^{AxBy}\right)-\varepsilon_{BB}^{B}p_{BB}^{AxBy}\left({\bar{P}}_{B}^{B}/{\bar{P}}_{B}^{AxBy}\right)\} \end{array}$$

All values on the right-hand side of Eq. (12) are known. *U, x* and *y* are readily available. $\Delta_{f}H\left(A_{x}B_{y}\right)$ and $\Delta_{f}H\left(i,gas\right)$ terms can be found in various sources [2], [3], [4], [5], [6], [7], [8], [9],[11], [12], [13], [14], [15], [16], [17]. The elemental bond enthalpies, $\varepsilon_{ii}^{i}$, can be calculated from Eq. (3b) and are listed in Table S1. The values of $p_{AA}^{AxBy}$, $p_{AB}^{AxBy}$ and $p_{BB}^{AxBy}$ have been counted using the approach detailed in [1] and are given in columns 8-10 of Table S1 for the compounds in this dataset. ${\bar{P}}_{A}^{AxBy}$ and ${\bar{P}}_{B}^{AxBy}$ are similarly counted and listed in Table S1 in columns 11 and 12. The derivation shown here corrects typographical errors in the earlier manuscript [1].

The method for counting the number of *i*—*j* bonds per *A_x_B_y_* unit cell, $p_{ij}^{AxBy}$, is described in [1]. Bonds that are within 125% of the minimum atomic separation for each of the A–A, A–B and B–B atom pairs are counted – longer bonds are excluded. Detailed atom separations used for bond counting are only available for the prototype compound of each structure [10], and so in [1] the $p_{ij}^{AxBy}$ values for the prototype compound are applied to all compounds with the same crystal structure, regardless of the relative sizes of A and B atoms in each distinct compound. However, in some structures, some bonds may systematically exceed the 125% criterion as the relative size of the A and B atoms change. For example, in the *cP*2 (ClCs prototype, Strukterbericht B2) structure, the distance between like atoms is always a_0_. If A and B atoms are of equal size, the distance between first neighbor A–A and B–B atoms is $2/\sqrt{3}=1.1547\cdots$ times the minimum separation and all bonds between like atoms are counted. However, if the radius of atom A is 80% that of atom B, then the separation between nearest A atoms is $2.25/\sqrt{3}=1.2990\cdots$ times the minimum A–A separation and the separation between nearest B atoms is $1.8/\sqrt{3}=1.0392\cdots$ times the minimum B–B separation. As a result, B–B bonds are counted but A–A bonds are not counted in this structure.

Corrections for the effect of relative atom size are applied here to four crystal structures, representing the most common crystal structure prototypes in the present dataset or structures with an unusually large range of relative atom sizes. These four structures account for 258 compounds in this dataset, or about 26% of the compounds. As a summary of these corrections:

• No corrections are needed for compounds with the *cF*24, Cu_2_Mg prototype structure;
• In compounds with the *cP*4, AuCu_3_ prototype structure, majority–majority atom bonds are always counted and minority–minority atom bonds are counted only when the majority atom-to-minority atom radius ratio is ≤0.768;
• In compounds with the *cP*2, ClSc prototype structure, bonds between large atoms are always counted and bonds between small atoms are only counted when the small-to-large atom radius ratio is ≥0.858;
• In compounds with the *cF*8, ClNa prototype structure, bonds between small atoms are never counted and bonds between large atoms are only counted when the small-to-large atom radius ratio is ≤0.768.

These corrections are included in Table S1 and are indicated in the Pearson Symbol (Prototype) column by cP2 ClSc_1 or cP2 ClSc_2; cP4 AuCu3_1 or cP4 AuCu3_2; and cF8 ClNa_1 or cF8 ClNa_2 as appropriate.

## Ethics Statements

No human or animal subjects were used in this work. This work did not involve data collection from social media platforms.

## CRediT authorship contribution statement

**Daniel Miracle:** Conceptualization, Methodology, Formal analysis, Data curation, Validation, Visualization, Writing – original draft, Writing – review & editing. **Amanda Dahlman:** Formal analysis, Data curation. **Garth Wilks:** Data curation, Visualization. **James E. Dahlman:** Formal analysis, Data curation.

## Declaration of Competing Interest

The authors declare that they have no known competing financial interests or personal relationships that could have appeared to influence the work reported in this paper.
