# Supplementary material for: Dataset of bond enthalpies (εAA, εAB, εBB) in 975 binary intermetallic compounds
Source: Data Brief. 2021 Nov 28;39:107652. doi: 10.1016/j.dib.2021.107652 (PMC8648938; doi:10.1016/j.dib.2021.107652)
Supplement: Supplementary Data S1 [file mmc1.docx]

Table 1 Condensed bond enthalpies between like atoms in pure elements ($\boldsymbol{\varepsilon}_{\boldsymbol{AA}}^{\boldsymbol{A}}$) and compounds ($\boldsymbol{\varepsilon}_{\boldsymbol{AA}}^{\boldsymbol{AxBy}}$, $\boldsymbol{\varepsilon}_{\boldsymbol{BB}}^{\boldsymbol{AxBy}}$); between unlike elements in compounds ($\boldsymbol{\varepsilon}_{\boldsymbol{AB}}$, $\boldsymbol{\varepsilon}_{\boldsymbol{AB,metal}}$); and the supporting data used to calculate these values (explained in the accompanying text). Modified from [1].

| **Element or Compound** | **Pearson Symbol (Prototype)** | **Element A in A_x_B_y_** | **Element B in A_x_B_y_** | **Δ_f_ H(A_x_ B_y_ ) or Δ_vap_ H(A) (kJ/mol)** | **±Δ_f_ H(A_x_ B_y_ ) or Δ_vap_ H(A) (kJ/mol)** | **Atom Fraction B** | $\boldsymbol{p}_{\boldsymbol{AA}}^{\boldsymbol{AxBy}}$ | $\boldsymbol{p}_{\boldsymbol{AB}}^{\boldsymbol{AxBy}}$ | $\boldsymbol{p}_{\boldsymbol{BB}}^{\boldsymbol{AxBy}}$ | ${\bar{\boldsymbol{P}}}_{\boldsymbol{A}}^{\boldsymbol{AxBy}}$ | ${\bar{\boldsymbol{P}}}_{\boldsymbol{B}}^{\boldsymbol{AxBy}}$ | $\boldsymbol{\varepsilon}_{\boldsymbol{AA}}^{\boldsymbol{A}}$ **or** $\boldsymbol{\varepsilon}_{\boldsymbol{AA}}^{\boldsymbol{AxBy}}$ **(eV/bond)** | $\boldsymbol{\pm\varepsilon}_{\boldsymbol{AA}}^{\boldsymbol{A}}$ **or** $\boldsymbol{\pm}\boldsymbol{\varepsilon}_{\boldsymbol{AA}}^{\boldsymbol{AxBy}}$ **(eV/bond)** | $\boldsymbol{\varepsilon}_{\boldsymbol{BB}}^{\boldsymbol{AxBy}}$ **(eV/bond)** | $\boldsymbol{\pm}\boldsymbol{\varepsilon}_{\boldsymbol{BB}}^{\boldsymbol{AxBy}}$ **(eV/bond)** | $\boldsymbol{\varepsilon}_{\boldsymbol{AB}}$ **(eV/bond)** | $\boldsymbol{\pm}\boldsymbol{\varepsilon}_{\boldsymbol{AB}}$ **(eV/bond)** | $\boldsymbol{\varepsilon}_{\boldsymbol{AB,metal}}$ **(eV/bond)** | $\boldsymbol{\pm}\boldsymbol{\varepsilon}_{\boldsymbol{AB,metal}}$ **(eV/bond)** |
| --- | --- | --- | --- | --- | --- | --- | --- | --- | --- | --- | --- | --- | --- | --- | --- | --- | --- | --- | --- |
| H (gas) |  |  |  | 218 | 0.0 | 0 |  |  |  | 1 |  | -2.259 | 0.000 |  |  |  |  |  |  |
| Li | cI2 (W) |  |  | 159.3 | 1.0 | 0 |  |  |  | 7 |  | -0.236 | 0.001 |  |  |  |  |  |  |
| Be | hP2 (Mg) |  |  | 324 | 5.0 | 0 |  |  |  | 6 |  | -0.560 | 0.009 |  |  |  |  |  |  |
| B | hR12 (B) |  |  | 565 | 5.0 | 0 |  |  |  | 3.25 |  | -1.802 | 0.016 |  |  |  |  |  |  |
| C | hP4 (C) |  |  | 716.68 | 0.5 | 0 |  |  |  | 1.5 |  | -4.952 | 0.003 |  |  |  |  |  |  |
| N (gas) |  |  |  | 472.68 | 0.4 | 0 |  |  |  | 1 |  | -4.899 | 0.004 |  |  |  |  |  |  |
| O (gas) |  |  |  | 249.23 | 0.0 | 0 |  |  |  | 1 |  | -2.583 | 0.000 |  |  |  |  |  |  |
| F (gas) |  |  |  | 79.38 | 0.3 | 0 |  |  |  | 1 |  | -0.823 | 0.003 |  |  |  |  |  |  |
| Na | cI2 (W) |  |  | 107.5 | 0.7 | 0 |  |  |  | 7 |  | -0.159 | 0.001 |  |  |  |  |  |  |
| Mg | hP2 (Mg) |  |  | 147.1 | 0.8 | 0 |  |  |  | 6 |  | -0.254 | 0.001 |  |  |  |  |  |  |
| Al | cF4 (Cu) |  |  | 330.9 | 4.0 | 0 |  |  |  | 6 |  | -0.572 | 0.007 |  |  |  |  |  |  |
| Si | cF8 (C) |  |  | 450 | 8.0 | 0 |  |  |  | 2 |  | -2.332 | 0.041 |  |  |  |  |  |  |
| P | oC8 (P) |  |  | 316.5 | 1.0 | 0 |  |  |  | 1.50 |  | -2.187 | 0.007 |  |  |  |  |  |  |
| S | oF128 (S) |  |  | 277.17 | 0.2 | 0 |  |  |  | 1 |  | -2.873 | 0.002 |  |  |  |  |  |  |
| Cl (gas) |  |  |  | 121.3 | 0.0 | 0 |  |  |  | 1 |  | -1.257 | 0.000 |  |  |  |  |  |  |
| K | cI2 (W) |  |  | 89 | 0.8 | 0 |  |  |  | 7 |  | -0.132 | 0.001 |  |  |  |  |  |  |
| Ca | cF4 (Cu) |  |  | 177.8 | 0.8 | 0 |  |  |  | 6 |  | -0.307 | 0.001 |  |  |  |  |  |  |
| Sc | hP2 (Mg) |  |  | 377.8 | 4.0 | 0 |  |  |  | 6 |  | -0.653 | 0.007 |  |  |  |  |  |  |
| Ti | hP2 (Mg) |  |  | 473 | 3.0 | 0 |  |  |  | 6 |  | -0.817 | 0.005 |  |  |  |  |  |  |
| V | cI2 (W) |  |  | 515.5 | 8.0 | 0 |  |  |  | 7 |  | -0.763 | 0.012 |  |  |  |  |  |  |
| Cr | cI2 (W) |  |  | 397.48 | 4.2 | 0 |  |  |  | 7 |  | -0.589 | 0.006 |  |  |  |  |  |  |
| Mn | cI58 (Mn) |  |  | 283.3 | 4.2 | 0 |  |  |  | 6.55 |  | -0.448 | 0.007 |  |  |  |  |  |  |
| Fe | cI2 (W) |  |  | 415.5 | 1.3 | 0 |  |  |  | 7 |  | -0.615 | 0.002 |  |  |  |  |  |  |
| Co | hP2 (Mg) |  |  | 426.7 | 8.5 | 0 |  |  |  | 6 |  | -0.737 | 0.015 |  |  |  |  |  |  |
| Ni | cF4 (Cu) |  |  | 430.1 | 8.4 | 0 |  |  |  | 6 |  | -0.743 | 0.015 |  |  |  |  |  |  |
| Cu | cF4 (Cu) |  |  | 337.4 | 1.2 | 0 |  |  |  | 6 |  | -0.583 | 0.002 |  |  |  |  |  |  |
| Zn | hP2 (Mg) |  |  | 130.4 | 0.4 | 0 |  |  |  | 6 |  | -0.225 | 0.001 |  |  |  |  |  |  |
| Ga | oC8 (Ga) |  |  | 271.96 | 2.1 | 0 |  |  |  | 3.5 |  | -0.805 | 0.006 |  |  |  |  |  |  |
| Ge | cF8 (C) |  |  | 372 | 3.0 | 0 |  |  |  | 2 |  | -1.928 | 0.016 |  |  |  |  |  |  |
| As | hR2 (As) |  |  | 302.5 | 13.0 | 0 |  |  |  | 3 |  | -1.045 | 0.045 |  |  |  |  |  |  |
| Se | hP3 (Se) |  |  | 227.2 | 4.0 | 0 |  |  |  | 1.5 |  | -1.570 | 0.028 |  |  |  |  |  |  |
| Br (gas) |  |  |  | 111.87 | 0.1 | 0 |  |  |  | 1 |  | -1.159 | 0.001 |  |  |  |  |  |  |
| Rb | cI2 (W) |  |  | 80.9 | 0.8 | 0 |  |  |  | 7 |  | -0.120 | 0.001 |  |  |  |  |  |  |
| Sr | cF4 (Cu) |  |  | 164 | 1.7 | 0 |  |  |  | 6 |  | -0.283 | 0.003 |  |  |  |  |  |  |
| Y | hP2 (Mg) |  |  | 424.7 | 2.1 | 0 |  |  |  | 6 |  | -0.734 | 0.004 |  |  |  |  |  |  |
| Zr | hP2 (Mg) |  |  | 610 | 8.4 | 0 |  |  |  | 6 |  | -1.054 | 0.015 |  |  |  |  |  |  |
| Nb | cI2 (W) |  |  | 733 | 8.0 | 0 |  |  |  | 7 |  | -1.085 | 0.012 |  |  |  |  |  |  |
| Mo | cI2 (W) |  |  | 658.98 | 3.8 | 0 |  |  |  | 7 |  | -0.976 | 0.006 |  |  |  |  |  |  |
| Tc | hP2 (Mg) |  |  | 678 | 13.6 | 0 |  |  |  | 6 |  | -1.171 | 0.023 |  |  |  |  |  |  |
| Ru | hP2 (Mg) |  |  | 650.6 | 6.3 | 0 |  |  |  | 6 |  | -1.124 | 0.011 |  |  |  |  |  |  |
| Rh | cF4 (Cu) |  |  | 556 | 4.0 | 0 |  |  |  | 6 |  | -0.960 | 0.007 |  |  |  |  |  |  |
| Pd | cF4 (Cu) |  |  | 376.6 | 2.1 | 0 |  |  |  | 6 |  | -0.651 | 0.004 |  |  |  |  |  |  |
| Ag | cF4 (Cu) |  |  | 284.9 | 0.8 | 0 |  |  |  | 6 |  | -0.492 | 0.001 |  |  |  |  |  |  |
| Cd | hP2 (Mg) |  |  | 111.8 | 0.2 | 0 |  |  |  | 6 |  | -0.193 | 0.000 |  |  |  |  |  |  |
| In | tI2 (In) |  |  | 243 | 4.0 | 0 |  |  |  | 6 |  | -0.420 | 0.007 |  |  |  |  |  |  |
| Sn | tI4 (Sn) |  |  | 301.2 | 1.5 | 0 |  |  |  | 5 |  | -0.624 | 0.003 |  |  |  |  |  |  |
| Sb | hR2 (As) |  |  | 264.4 | 2.5 | 0 |  |  |  | 3 |  | -0.913 | 0.009 |  |  |  |  |  |  |
| Te | hP3 (Se) |  |  | 196.6 | 2.1 | 0 |  |  |  | 1.5 |  | -1.358 | 0.015 |  |  |  |  |  |  |
| I (gas) |  |  |  | 106.76 | 0.0 | 0 |  |  |  | 1 |  | -1.107 | 0.000 |  |  |  |  |  |  |
| Cs | cI2 (W) |  |  | 76.5 | 1.0 | 0 |  |  |  | 7 |  | -0.113 | 0.001 |  |  |  |  |  |  |
| Ba | cI2 (W) |  |  | 179.1 | 5.0 | 0 |  |  |  | 7 |  | -0.265 | 0.007 |  |  |  |  |  |  |
| La | hP4 (La) |  |  | 431 | 2.1 | 0 |  |  |  | 6 |  | -0.745 | 0.004 |  |  |  |  |  |  |
| Ce | hP2 (Mg) |  |  | 420.1 | 2.1 | 0 |  |  |  | 6 |  | -0.726 | 0.004 |  |  |  |  |  |  |
| Pr | hP4 (La) |  |  | 356.9 | 2.1 | 0 |  |  |  | 6 |  | -0.617 | 0.004 |  |  |  |  |  |  |
| Nd | hP4 (La) |  |  | 326.9 | 2.1 | 0 |  |  |  | 6 |  | -0.565 | 0.004 |  |  |  |  |  |  |
| Sm | hR3 (Sm) |  |  | 206.7 | 2.1 | 0 |  |  |  | 6 |  | -0.357 | 0.004 |  |  |  |  |  |  |
| Eu | cI2 (W) |  |  | 177.4 | 2.1 | 0 |  |  |  | 7 |  | -0.263 | 0.003 |  |  |  |  |  |  |
| Gd | hP2 (Mg) |  |  | 397.5 | 2.1 | 0 |  |  |  | 6 |  | -0.687 | 0.004 |  |  |  |  |  |  |
| Tb | hP2 (Mg) |  |  | 388.7 | 2.1 | 0 |  |  |  | 6 |  | -0.671 | 0.004 |  |  |  |  |  |  |
| Dy | hP2 (Mg) |  |  | 290.4 | 2.1 | 0 |  |  |  | 6 |  | -0.502 | 0.004 |  |  |  |  |  |  |
| Ho | hP2 (Mg) |  |  | 300.6 | 2.1 | 0 |  |  |  | 6 |  | -0.519 | 0.004 |  |  |  |  |  |  |
| Er | hP2 (Mg) |  |  | 316.4 | 2.1 | 0 |  |  |  | 6 |  | -0.547 | 0.004 |  |  |  |  |  |  |
| Tm | hP2 (Mg) |  |  | 232.2 | 2.1 | 0 |  |  |  | 6 |  | -0.401 | 0.004 |  |  |  |  |  |  |
| Yb | cF4 (Cu) |  |  | 155.6 | 2.1 | 0 |  |  |  | 6 |  | -0.269 | 0.004 |  |  |  |  |  |  |
| Lu | hP2 (Mg) |  |  | 427.6 | 2.1 | 0 |  |  |  | 6 |  | -0.739 | 0.004 |  |  |  |  |  |  |
| Hf | hP2 (Mg) |  |  | 618.4 | 6.3 | 0 |  |  |  | 6 |  | -1.068 | 0.011 |  |  |  |  |  |  |
| Ta | cI2 (W) |  |  | 782 | 2.5 | 0 |  |  |  | 7 |  | -1.158 | 0.004 |  |  |  |  |  |  |
| W | cI2 (W) |  |  | 851 | 6.3 | 0 |  |  |  | 7 |  | -1.260 | 0.009 |  |  |  |  |  |  |
| Re | hP2 (Mg) |  |  | 774 | 6.3 | 0 |  |  |  | 6 |  | -1.337 | 0.011 |  |  |  |  |  |  |
| Os | hP2 (Mg) |  |  | 787 | 6.3 | 0 |  |  |  | 6 |  | -1.359 | 0.011 |  |  |  |  |  |  |
| Ir | cF4 (Cu) |  |  | 669 | 4.0 | 0 |  |  |  | 6 |  | -1.156 | 0.007 |  |  |  |  |  |  |
| Pt | cF4 (Cu) |  |  | 565.7 | 1.3 | 0 |  |  |  | 6 |  | -0.977 | 0.002 |  |  |  |  |  |  |
| Au | cF4 (Cu) |  |  | 368.2 | 2.1 | 0 |  |  |  | 6 |  | -0.636 | 0.004 |  |  |  |  |  |  |
| Hg | hR1 (Hg) |  |  | 61.38 | 0.0 | 0 |  |  |  | 6 |  | -0.106 | 0.000 |  |  |  |  |  |  |
| Tl | hP2 (Mg) |  |  | 182.2 | 0.4 | 0 |  |  |  | 6 |  | -0.315 | 0.001 |  |  |  |  |  |  |
| Pb | cF4 (Cu) |  |  | 195.2 | 0.8 | 0 |  |  |  | 6 |  | -0.337 | 0.001 |  |  |  |  |  |  |
| Bi | mC4 (Bi) |  |  | 209.6 | 2.1 | 0 |  |  |  | 4.5 |  | -0.483 | 0.005 |  |  |  |  |  |  |
| Po | cP1 (Po) |  |  | 142 | 2.8 | 0 |  |  |  | 3 |  | -0.491 | 0.010 |  |  |  |  |  |  |
| Ra | cI2 (W) |  |  | 159 | 3.2 | 0 |  |  |  | 7 |  | -0.235 | 0.005 |  |  |  |  |  |  |
| Ac | cF4 (Cu) |  |  | 406 | 8.1 | 0 |  |  |  | 6 |  | -0.701 | 0.014 |  |  |  |  |  |  |
| Th | cF4 (Cu) |  |  | 602 | 6.0 | 0 |  |  |  | 6 |  | -1.040 | 0.010 |  |  |  |  |  |  |
| Pa | tI2 (Pa) |  |  | 563 | 11.3 | 0 |  |  |  | 7 |  | -0.834 | 0.017 |  |  |  |  |  |  |
| U | oC4 (U) |  |  | 533 | 8.0 | 0 |  |  |  | 6 |  | -0.921 | 0.014 |  |  |  |  |  |  |
| Np | oP8 (Np) |  |  | 464.8 | 9.3 | 0 |  |  |  | 7.5 |  | -0.642 | 0.013 |  |  |  |  |  |  |
| Pu | mP16 (Pu) |  |  | 345 | 6.9 | 0 |  |  |  | 7 |  | -0.511 | 0.010 |  |  |  |  |  |  |
| AgBa | oP8 BFe | Ba | Ag | -17 | 1.0 | 0.5 | 20 | 28 | 4 | 8.50 | 4.50 | -0.218 | 0.007 | -0.656 | 0.001 | -0.463 | 0.016 | -0.025 | 0.001 |
| Ag5Ba | hP6 CaCu5 | Ba | Ag | -11 | 0.7 | 0.833 | 0 | 18 | 21 | 9.00 | 6.00 | -0.206 | 0.007 | -0.492 | 0.001 | -0.356 | 0.007 | -0.006 | 0.000 |
| Ag3Be8 | cF24 Cu2Mg | Ag | Be | 3 | 0.2 | 0.727 | 16 | 96 | 48 | 9.78 | 5.50 | -0.302 | 0.001 | -0.611 | 0.009 | -0.456 | 0.015 | 0.001 | 0.000 |
| Ag3Ca5 | tI32 B3Cr5 | Ca | Ag | -22 | 1.3 | 0.375 | 100 | 104 | 4 | 7.60 | 4.67 | -0.242 | 0.006 | -0.633 | 0.001 | -0.446 | 0.019 | -0.009 | 0.001 |
| AgCa | oC8 BCr | Ca | Ag | -26 | 1.5 | 0.5 | 20 | 28 | 4 | 8.50 | 4.50 | -0.217 | 0.001 | -0.656 | 0.001 | -0.474 | 0.006 | -0.038 | 0.002 |
| Ag2Ca | oI12 Hg2K | Ca | Ag | -23 | 1.4 | 0.667 | 8 | 48 | 24 | 8.00 | 6.00 | -0.230 | 0.001 | -0.492 | 0.001 | -0.381 | 0.004 | -0.020 | 0.001 |
| Ag2Cd3 | cI52 Cu5Zn8 | Cd | Ag | -6 | 0.4 | 0.4 | 84 | 192 | 30 | 5.77 | 6.06 | -0.201 | 0.000 | -0.487 | 0.001 | -0.348 | 0.002 | -0.004 | 0.000 |
| Ag2Ce | oI12 CeCu2 | Ag | Ce | -20 | 1.2 | 0.333 | 16 | 48 | 8 | 5.00 | 8.00 | -0.591 | 0.001 | -0.544 | 0.004 | -0.585 | 0.005 | -0.017 | 0.001 |
| AgCe | cP2 ClCs_2 | Ag | Ce | -22 | 1.3 | 0.5 | 0 | 8 | 3 | 4.00 | 7.00 | -0.738 | 0.001 | -0.622 | 0.004 | -0.709 | 0.007 | -0.029 | 0.002 |
| AgIn2 | tI12 Al2Cu | In | Ag | -4 | 0.2 | 0.333 | 44 | 32 | 4 | 7.50 | 5.00 | -0.336 | 0.007 | -0.591 | 0.001 | -0.468 | 0.021 | -0.005 | 0.000 |
| Ag2In | cP52 Al4Cu9 | In | Ag | -7 | 0.4 | 0.667 | 6 | 168 | 132 | 5.19 | 6.23 | -0.485 | 0.007 | -0.474 | 0.001 | -0.487 | 0.008 | -0.008 | 0.000 |
| AgLa | cP2 ClCs_2 | Ag | La | -17 | 2.3 | 0.5 | 0 | 8 | 3 | 4.00 | 7.00 | -0.738 | 0.001 | -0.638 | 0.004 | -0.710 | 0.008 | -0.022 | 0.003 |
| AgPr | cP2 ClCs_2 | Ag | Pr | -24 | 2.8 | 0.5 | 0 | 8 | 3 | 4.00 | 7.00 | -0.738 | 0.001 | -0.528 | 0.004 | -0.665 | 0.009 | -0.032 | 0.004 |
| Ag2Sc | tI6 MoSi2 | Ag | Sc | -27 | 0.6 | 0.333 | 18 | 20 | 4 | 7.00 | 7.00 | -0.422 | 0.001 | -0.559 | 0.007 | -0.519 | 0.009 | -0.028 | 0.001 |
| AgSc | cP2 ClCs_1 | Ag | Sc | -26 | 1.6 | 0.5 | 3 | 8 | 3 | 7.00 | 7.00 | -0.422 | 0.001 | -0.559 | 0.007 | -0.525 | 0.011 | -0.034 | 0.002 |
| AgTi | tP4 AuCu | Ag | Ti | -2 | 0.1 | 0.5 | 4 | 16 | 4 | 6.00 | 6.00 | -0.492 | 0.001 | -0.817 | 0.005 | -0.657 | 0.007 | -0.002 | 0.000 |
| AgTi2 | tI6 MoSi2 | Ag | Ti | -1 | 0.1 | 0.667 | 4 | 20 | 18 | 7.00 | 7.00 | -0.422 | 0.001 | -0.700 | 0.005 | -0.562 | 0.012 | -0.001 | 0.000 |
| AgY | cP2 ClCs_2 | Ag | Y | -27 | 3.2 | 0.5 | 0 | 8 | 3 | 4.00 | 7.00 | -0.738 | 0.001 | -0.629 | 0.004 | -0.718 | 0.009 | -0.035 | 0.004 |
| AgZr | tP4 CuTi | Ag | Zr | -7 | 0.4 | 0.5 | 8 | 12 | 8 | 7.00 | 7.00 | -0.422 | 0.001 | -0.903 | 0.015 | -0.674 | 0.027 | -0.012 | 0.001 |
| AgZr2 | tI6 MoSi2 | Ag | Zr | -9 | 0.5 | 0.667 | 4 | 20 | 18 | 7.00 | 7.00 | -0.422 | 0.001 | -0.903 | 0.015 | -0.672 | 0.032 | -0.009 | 0.001 |
| AlAs | cF8 SZn | Al | As | -59 | 3.5 | 0.5 | 0 | 16 | 0 | 2.00 | 2.00 | -1.715 | 0.007 | -1.568 | 0.045 | -1.793 | 0.053 | -0.152 | 0.009 |
| Al2Au | cF12 CaF2 | Al | Au | -22 | 1.3 | 0.333 | 24 | 32 | 24 | 5.00 | 10.00 | -0.686 | 0.007 | -0.382 | 0.004 | -0.563 | 0.023 | -0.029 | 0.002 |
| AlAu | cP2 ClCs_1 | Al | Au | -31 | 1.8 | 0.5 | 3 | 8 | 3 | 7.00 | 7.00 | -0.490 | 0.007 | -0.545 | 0.004 | -0.557 | 0.014 | -0.040 | 0.002 |
| AlB2 | hP3 AlB2 | Al | B | -11 | 0.7 | 0.667 | 4 | 12 | 3 | 10.00 | 4.50 | -0.343 | 0.007 | -1.301 | 0.016 | -0.832 | 0.019 | -0.010 | 0.001 |
| Al4C3 | hR7 Al4C3 | Al | C | -31 | 1.9 | 0.429 | 0 | 16 | 0 | 2.00 | 2.67 | -1.715 | 0.007 | -2.785 | 0.003 | -2.271 | 0.012 | -0.020 | 0.001 |
| Al2Ca | cF24 Cu2Mg | Ca | Al | -30 | 1.8 | 0.667 | 16 | 96 | 48 | 8.00 | 6.00 | -0.230 | 0.001 | -0.572 | 0.007 | -0.427 | 0.013 | -0.026 | 0.002 |
| Al4Ca | tI10 Al4Ba | Ca | Al | -21 | 1.2 | 0.8 | 4 | 34 | 26 | 10.50 | 5.38 | -0.176 | 0.001 | -0.638 | 0.007 | -0.420 | 0.016 | -0.013 | 0.001 |
| Al11Ce3 | oI28 Al11La3 | Al | Ce | -40 | 2.4 | 0.214 | 88 | 96 | 12 | 6.18 | 10.00 | -0.555 | 0.007 | -0.435 | 0.019 | -0.504 | 0.029 | -0.009 | 0.001 |
| Al3Ce | hP8 Ni3Sn | Al | Ce | -44 | 2.6 | 0.25 | 24 | 24 | 0 | 6.00 | 6.00 | -0.572 | 0.007 | -0.726 | 0.004 | -0.686 | 0.021 | -0.038 | 0.002 |
| Al2Ce | cF24 Cu2Mg | Al | Ce | -52 | 3.1 | 0.333 | 48 | 96 | 16 | 6.00 | 8.00 | -0.572 | 0.007 | -0.544 | 0.004 | -0.603 | 0.015 | -0.045 | 0.003 |
| AlCe | oC16 AlCe | Al | Ce | -46 | 2.7 | 0.5 | 12 | 64 | 28 | 5.50 | 7.50 | -0.624 | 0.007 | -0.581 | 0.004 | -0.662 | 0.014 | -0.059 | 0.004 |
| AlCe3 | hP8 Ni3Sn | Al | Ce | -25 | 1.5 | 0.75 | 0 | 24 | 24 | 6.00 | 6.00 | -0.572 | 0.007 | -0.726 | 0.004 | -0.670 | 0.014 | -0.022 | 0.001 |
| Al9Co2 | mP22 Al9Co2 | Al | Co | -34 | 2.0 | 0.182 | 99 | 36 | 0 | 6.50 | 4.50 | -0.528 | 0.014 | -0.983 | 0.015 | -0.775 | 0.113 | -0.020 | 0.001 |
| Al5Co2 | hP28 Al5Co2 | Al | Co | -45 | 2.7 | 0.286 | 96 | 78 | 6 | 6.75 | 5.63 | -0.508 | 0.007 | -0.786 | 0.015 | -0.671 | 0.031 | -0.024 | 0.001 |
| AlCo | cP2 ClCs_1 | Al | Co | -58 | 3.5 | 0.5 | 3 | 8 | 3 | 7.00 | 7.00 | -0.490 | 0.007 | -0.632 | 0.015 | -0.637 | 0.029 | -0.076 | 0.004 |
| Al13Cr2 | mC104 Al45V7 | Al | Cr | -12 | 0.7 | 0.133 | 456 | 160 | 4 | 5.95 | 6.06 | -0.577 | 0.007 | -0.680 | 0.006 | -0.634 | 0.047 | -0.005 | 0.000 |
| AlCr2 | tI6 MoSi2 | Al | Cr | -11 | 0.6 | 0.667 | 4 | 20 | 18 | 7.00 | 7.00 | -0.490 | 0.007 | -0.589 | 0.006 | -0.551 | 0.020 | -0.011 | 0.001 |
| Al2Cu | tI12 Al2Cu | Al | Cu | -16 | 0.9 | 0.333 | 44 | 32 | 4 | 7.50 | 5.00 | -0.457 | 0.007 | -0.699 | 0.002 | -0.599 | 0.023 | -0.020 | 0.001 |
| AlCu | mC20 AlCu | Al | Cu | -20 | 1.2 | 0.5 | 22 | 68 | 22 | 5.60 | 5.60 | -0.612 | 0.007 | -0.624 | 0.002 | -0.649 | 0.013 | -0.031 | 0.002 |
| Al4Cu9 | cP52 Al4Cu9 | Al | Cu | -23 | 1.4 | 0.692 | 6 | 168 | 132 | 5.63 | 6.00 | -0.610 | 0.007 | -0.583 | 0.002 | -0.602 | 0.009 | -0.006 | 0.000 |
| AlDy2 | oP12 Co2Si | Dy | Al | -38 | 2.3 | 0.333 | 32 | 40 | 0 | 6.50 | 5.00 | -0.463 | 0.004 | -0.686 | 0.007 | -0.614 | 0.014 | -0.040 | 0.002 |
| Al2Dy3 | tP20 Al2Gd3 | Dy | Al | -44 | 2.6 | 0.4 | 54 | 64 | 6 | 7.17 | 4.75 | -0.420 | 0.004 | -0.722 | 0.022 | -0.599 | 0.039 | -0.028 | 0.002 |
| AlDy | oP16 AlDy | Dy | Al | -49 | 2.9 | 0.5 | 28 | 64 | 12 | 7.50 | 5.50 | -0.401 | 0.004 | -0.624 | 0.007 | -0.577 | 0.015 | -0.064 | 0.004 |
| Al2Dy | cF24 Cu2Mg | Dy | Al | -54 | 3.2 | 0.667 | 16 | 96 | 48 | 8.00 | 6.00 | -0.376 | 0.004 | -0.572 | 0.007 | -0.521 | 0.016 | -0.047 | 0.003 |
| Al3Dy | hP16 Ni3Ti | Dy | Al | -42 | 2.5 | 0.75 | 0 | 48 | 48 | 6.00 | 6.00 | -0.502 | 0.004 | -0.572 | 0.007 | -0.573 | 0.021 | -0.037 | 0.002 |
| Al2Er | cF24 Cu2Mg | Er | Al | -50 | 3.0 | 0.667 | 16 | 96 | 48 | 8.00 | 6.00 | -0.410 | 0.004 | -0.572 | 0.007 | -0.534 | 0.015 | -0.043 | 0.003 |
| Al2Eu | cF24 Cu2Mg | Eu | Al | -36 | 2.1 | 0.667 | 16 | 96 | 48 | 8.00 | 6.00 | -0.230 | 0.003 | -0.572 | 0.007 | -0.432 | 0.015 | -0.031 | 0.002 |
| Al5Fe2 | oC16 Al5Fe2 | Al | Fe | -30 | 1.8 | 0.286 | 60 | 40 | 4 | 7.00 | 5.25 | -0.490 | 0.025 | -0.820 | 0.002 | -0.673 | 0.100 | -0.018 | 0.001 |
| Al2Fe | aP18 Al2Fe | Al | Fe | -30 | 1.8 | 0.333 | 41 | 50 | 10 | 5.50 | 5.75 | -0.624 | 0.007 | -0.749 | 0.002 | -0.723 | 0.020 | -0.037 | 0.002 |
| AlFe | cP2 ClCs_1 | Al | Fe | -25 | 1.5 | 0.5 | 3 | 8 | 3 | 7.00 | 7.00 | -0.490 | 0.007 | -0.615 | 0.002 | -0.585 | 0.012 | -0.032 | 0.002 |
| AlFe3 | cF16 BiF3 | Al | Fe | -13 | 0.8 | 0.75 | 0 | 56 | 56 | 7.00 | 7.00 | -0.490 | 0.007 | -0.615 | 0.002 | -0.562 | 0.008 | -0.010 | 0.001 |
| Al3Gd | hP8 Ni3Sn | Al | Gd | -41 | 2.4 | 0.25 | 24 | 24 | 0 | 6.00 | 6.00 | -0.572 | 0.007 | -0.687 | 0.004 | -0.665 | 0.021 | -0.035 | 0.002 |
| Al2Gd | cF24 Cu2Mg | Al | Gd | -53 | 3.1 | 0.333 | 48 | 96 | 16 | 6.00 | 8.00 | -0.572 | 0.007 | -0.515 | 0.004 | -0.589 | 0.015 | -0.046 | 0.003 |
| AlGd | oP16 AlDy | Al | Gd | -49 | 2.9 | 0.5 | 12 | 64 | 28 | 5.50 | 7.50 | -0.624 | 0.007 | -0.549 | 0.004 | -0.649 | 0.015 | -0.063 | 0.004 |
| Al2Gd3 | tP20 Al2Gd3 | Al | Gd | -44 | 2.6 | 0.6 | 6 | 64 | 54 | 4.75 | 7.17 | -0.722 | 0.038 | -0.575 | 0.004 | -0.677 | 0.040 | -0.028 | 0.002 |
| AlGd2 | oP12 Co2Si | Al | Gd | -38 | 2.3 | 0.667 | 0 | 40 | 32 | 5.00 | 6.50 | -0.686 | 0.007 | -0.634 | 0.004 | -0.699 | 0.014 | -0.039 | 0.002 |
| Al3Hf | tI16 Al3Zr | Al | Hf | -41 | 2.4 | 0.25 | 48 | 48 | 0 | 6.00 | 6.00 | -0.572 | 0.007 | -1.068 | 0.011 | -0.856 | 0.025 | -0.036 | 0.002 |
| Al2Hf | hP12 MgZn2 | Al | Hf | -46 | 2.7 | 0.333 | 24 | 48 | 8 | 6.00 | 8.00 | -0.572 | 0.007 | -0.801 | 0.011 | -0.726 | 0.020 | -0.040 | 0.002 |
| Al3Hf2 | oF40 Al3Zr2 | Al | Hf | -48 | 2.8 | 0.4 | 52 | 148 | 56 | 5.25 | 8.13 | -0.653 | 0.031 | -0.789 | 0.011 | -0.748 | 0.068 | -0.027 | 0.002 |
| AlHf | oC8 BCr | Al | Hf | -47 | 2.0 | 0.5 | 4 | 28 | 20 | 4.50 | 8.50 | -0.762 | 0.007 | -0.754 | 0.011 | -0.827 | 0.027 | -0.069 | 0.003 |
| Al3Hf4 | hP7 Al3Zr4 | Al | Hf | -46 | 2.7 | 0.571 | 6 | 24 | 17 | 6.00 | 7.25 | -0.572 | 0.007 | -0.884 | 0.011 | -0.748 | 0.027 | -0.020 | 0.001 |
| Al2Hf3 | tP20 Al2Zr3 | Al | Hf | -44 | 2.6 | 0.6 | 8 | 64 | 59 | 5.00 | 7.58 | -0.686 | 0.007 | -0.845 | 0.038 | -0.794 | 0.121 | -0.029 | 0.002 |
| AlHf2 | tI12 Al2Cu | Al | Hf | -41 | 2.4 | 0.667 | 4 | 32 | 44 | 5.00 | 7.50 | -0.686 | 0.007 | -0.855 | 0.011 | -0.823 | 0.040 | -0.053 | 0.003 |
| AlHo2 | oP12 Co2Si | Ho | Al | -40 | 2.4 | 0.333 | 32 | 40 | 0 | 6.50 | 5.00 | -0.479 | 0.004 | -0.686 | 0.007 | -0.624 | 0.014 | -0.041 | 0.002 |
| Al2Ho3 | tP20 Al2Gd3 | Ho | Al | -45 | 2.6 | 0.4 | 54 | 64 | 6 | 7.17 | 4.75 | -0.435 | 0.004 | -0.722 | 0.023 | -0.607 | 0.039 | -0.029 | 0.002 |
| AlHo | oP16 AlDy | Ho | Al | -51 | 3.0 | 0.5 | 28 | 64 | 12 | 7.50 | 5.50 | -0.415 | 0.004 | -0.624 | 0.007 | -0.585 | 0.015 | -0.065 | 0.004 |
| Al2Ho | cF24 Cu2Mg | Ho | Al | -56 | 3.3 | 0.667 | 16 | 96 | 48 | 8.00 | 6.00 | -0.389 | 0.004 | -0.572 | 0.007 | -0.529 | 0.016 | -0.048 | 0.003 |
| Al3Ho | hR20 Al3Ho | Ho | Al | -32 | 1.9 | 0.75 | 0 | 60 | 60 | 6.00 | 6.00 | -0.519 | 0.004 | -0.572 | 0.007 | -0.573 | 0.021 | -0.028 | 0.002 |
| Al11La3 | oI28 Al11La3 | Al | La | -39 | 2.3 | 0.214 | 88 | 96 | 12 | 6.18 | 10.00 | -0.555 | 0.007 | -0.447 | 0.019 | -0.509 | 0.029 | -0.008 | 0.001 |
| Al2La | cF24 Cu2Mg | Al | La | -51 | 3.0 | 0.333 | 48 | 96 | 16 | 6.00 | 8.00 | -0.572 | 0.007 | -0.558 | 0.004 | -0.609 | 0.015 | -0.044 | 0.003 |
| AlLa3 | hP8 Ni3Sn | Al | La | -25 | 1.5 | 0.75 | 0 | 24 | 24 | 6.00 | 6.00 | -0.572 | 0.007 | -0.745 | 0.004 | -0.680 | 0.014 | -0.022 | 0.001 |
| Al2Li3 | hR5 Ga2Li3 | Li | Al | -18 | 1.1 | 0.4 | 10 | 22 | 3 | 7.00 | 7.00 | -0.236 | 0.001 | -0.490 | 0.007 | -0.371 | 0.007 | -0.008 | 0.001 |
| AlLi | cF16 NaTl | Li | Al | -21 | 1.2 | 0.5 | 16 | 80 | 16 | 7.00 | 7.00 | -0.236 | 0.001 | -0.490 | 0.007 | -0.384 | 0.008 | -0.021 | 0.001 |
| Al11Mn4 | aP15 Al11Mn4 | Mn | Al | -23 | 1.3 | 0.733 | 5 | 40 | 57 | 6.25 | 7.00 | -0.470 | 0.007 | -0.490 | 0.007 | -0.486 | 0.027 | -0.006 | 0.000 |
| Al6Mn | oC28 Al6Mn | Mn | Al | -15 | 0.9 | 0.857 | 0 | 36 | 112 | 4.50 | 5.42 | -0.652 | 0.007 | -0.633 | 0.007 | -0.660 | 0.055 | -0.017 | 0.001 |
| Al12Mo | cI26 Al12W | Al | Mo | -11 | 0.6 | 0.077 | 120 | 24 | 0 | 5.50 | 6.00 | -0.624 | 0.007 | -1.138 | 0.006 | -0.890 | 0.080 | -0.009 | 0.001 |
| Al5Mo | hP12 Al5W | Al | Mo | -23 | 1.4 | 0.167 | 48 | 24 | 0 | 6.00 | 6.00 | -0.572 | 0.007 | -1.138 | 0.006 | -0.875 | 0.036 | -0.020 | 0.001 |
| Al4Mo | mC30 Al4W | Al | Mo | -28 | 1.6 | 0.2 | 120 | 64 | 4 | 6.33 | 6.00 | -0.542 | 0.007 | -1.138 | 0.006 | -0.867 | 0.034 | -0.027 | 0.002 |
| Al8Mo3 | mC22 Al8Mo3 | Al | Mo | -38 | 2.2 | 0.273 | 62 | 68 | 2 | 6.00 | 6.00 | -0.572 | 0.007 | -1.138 | 0.006 | -0.866 | 0.020 | -0.011 | 0.001 |
| AlMo3 | cP8 Cr3Si | Al | Mo | -22 | 1.3 | 0.75 | 0 | 24 | 30 | 6.00 | 7.00 | -0.572 | 0.007 | -0.976 | 0.006 | -0.793 | 0.021 | -0.019 | 0.001 |
| Al3Nb | tI8 Al3Ti | Al | Nb | -33 | 2.0 | 0.25 | 24 | 24 | 0 | 6.00 | 6.00 | -0.572 | 0.007 | -1.266 | 0.012 | -0.947 | 0.026 | -0.029 | 0.002 |
| AlNb2 | tP30 CrFe-a | Al | Nb | -25 | 1.5 | 0.667 | 12 | 94 | 88 | 5.90 | 6.75 | -0.581 | 0.010 | -1.125 | 0.030 | -0.881 | 0.144 | -0.028 | 0.002 |
| AlNb3 | cP8 Cr3Si | Al | Nb | -19 | 1.1 | 0.75 | 0 | 24 | 30 | 6.00 | 7.00 | -0.572 | 0.007 | -1.085 | 0.012 | -0.845 | 0.040 | -0.016 | 0.001 |
| AlNd3 | hP8 Ni3Sn | Nd | Al | -27 | 1.6 | 0.25 | 24 | 24 | 0 | 6.00 | 6.00 | -0.565 | 0.004 | -0.572 | 0.007 | -0.591 | 0.014 | -0.023 | 0.001 |
| AlNd2 | oP12 Co2Si | Nd | Al | -36 | 2.1 | 0.333 | 32 | 40 | 0 | 6.50 | 5.00 | -0.521 | 0.004 | -0.686 | 0.007 | -0.641 | 0.014 | -0.037 | 0.002 |
| AlNd | oP16 AlDy | Nd | Al | -50 | 3.0 | 0.5 | 28 | 64 | 12 | 7.50 | 5.50 | -0.452 | 0.004 | -0.624 | 0.007 | -0.602 | 0.015 | -0.065 | 0.004 |
| Al2Nd | cF24 Cu2Mg | Nd | Al | -53 | 3.1 | 0.667 | 16 | 96 | 48 | 8.00 | 6.00 | -0.424 | 0.004 | -0.572 | 0.007 | -0.543 | 0.015 | -0.046 | 0.003 |
| Al3Nd | hP8 Ni3Sn | Nd | Al | -45 | 2.7 | 0.75 | 0 | 24 | 24 | 6.00 | 6.00 | -0.565 | 0.004 | -0.572 | 0.007 | -0.607 | 0.021 | -0.039 | 0.002 |
| Al11Nd3 | oI28 Al11La3 | Nd | Al | -39 | 2.3 | 0.786 | 12 | 96 | 88 | 10.00 | 6.18 | -0.339 | 0.011 | -0.555 | 0.007 | -0.455 | 0.026 | -0.008 | 0.001 |
| Al3Ni | oP16 CFe3 | Al | Ni | -48 | 2.8 | 0.25 | 68 | 34 | 2 | 7.08 | 4.75 | -0.484 | 0.007 | -0.938 | 0.078 | -0.770 | 0.148 | -0.059 | 0.007 |
| Al3Ni2 | hP5 Al3Ni2 | Al | Ni | -61 | 3.6 | 0.4 | 9 | 16 | 3 | 5.67 | 5.50 | -0.605 | 0.007 | -0.810 | 0.015 | -0.748 | 0.028 | -0.040 | 0.002 |
| AlNi | cP2 ClCs_1 | Al | Ni | -66 | 3.9 | 0.5 | 3 | 8 | 3 | 7.00 | 7.00 | -0.490 | 0.007 | -0.637 | 0.015 | -0.649 | 0.029 | -0.085 | 0.005 |
| Al3Ni5 | oC16 Ga3Pt5 | Al | Ni | -54 | 3.2 | 0.625 | 8 | 56 | 32 | 6.00 | 6.00 | -0.572 | 0.007 | -0.743 | 0.015 | -0.677 | 0.030 | -0.020 | 0.001 |
| AlNi3 | cP4 AuCu3_2 | Al | Ni | -41 | 2.4 | 0.75 | 0 | 12 | 12 | 6.00 | 6.00 | -0.572 | 0.007 | -0.743 | 0.015 | -0.693 | 0.042 | -0.035 | 0.002 |
| AlP | cF8 SZn | Al | P | -82 | 4.9 | 0.5 | 0 | 16 | 0 | 2.00 | 2.00 | -1.715 | 0.007 | -1.640 | 0.007 | -1.890 | 0.026 | -0.213 | 0.013 |
| Al11Pr3 | oI28 Al11La3 | Al | Pr | -44 | 2.6 | 0.214 | 88 | 96 | 12 | 6.18 | 10.00 | -0.555 | 0.007 | -0.370 | 0.019 | -0.472 | 0.028 | -0.009 | 0.001 |
| Al2Pr | cF24 Cu2Mg | Al | Pr | -54 | 3.2 | 0.333 | 48 | 96 | 16 | 6.00 | 8.00 | -0.572 | 0.007 | -0.462 | 0.004 | -0.564 | 0.016 | -0.047 | 0.003 |
| Al3Pt2 | hP5 Al3Ni2 | Al | Pt | -87 | 5.2 | 0.4 | 9 | 16 | 3 | 5.67 | 5.50 | -0.605 | 0.007 | -1.066 | 0.002 | -0.892 | 0.017 | -0.056 | 0.003 |
| AlPt | cP8 FeSi | Al | Pt | -102 | 6.0 | 0.5 | 12 | 28 | 12 | 6.50 | 6.50 | -0.528 | 0.007 | -0.902 | 0.002 | -0.866 | 0.021 | -0.151 | 0.009 |
| Al3Pt5 | oP16 Ge3Rh5 | Al | Pt | -88 | 5.2 | 0.625 | 5 | 57 | 38 | 5.58 | 6.65 | -0.614 | 0.104 | -0.882 | 0.005 | -0.780 | 0.088 | -0.032 | 0.002 |
| Al6Ru | oC28 Al6Mn | Al | Ru | -28 | 1.7 | 0.143 | 112 | 36 | 0 | 5.42 | 4.50 | -0.633 | 0.007 | -1.498 | 0.011 | -1.098 | 0.058 | -0.032 | 0.002 |
| Al2Ru | oF24 Si2Ti | Al | Ru | -51 | 3.0 | 0.333 | 40 | 80 | 16 | 5.00 | 7.00 | -0.686 | 0.007 | -0.963 | 0.011 | -0.877 | 0.024 | -0.053 | 0.003 |
| AlRu | cP2 ClCs_1 | Al | Ru | -62 | 3.7 | 0.5 | 3 | 8 | 3 | 7.00 | 7.00 | -0.490 | 0.007 | -0.963 | 0.011 | -0.807 | 0.025 | -0.080 | 0.005 |
| AlSb | cF8 SZn | Al | Sb | -24 | 1.5 | 0.5 | 0 | 16 | 0 | 2.00 | 2.00 | -1.715 | 0.007 | -1.370 | 0.009 | -1.606 | 0.021 | -0.063 | 0.004 |
| Al3Sc | cP4 AuCu3_2 | Al | Sc | -42 | 2.5 | 0.25 | 12 | 12 | 0 | 6.00 | 6.00 | -0.572 | 0.007 | -0.653 | 0.007 | -0.648 | 0.023 | -0.036 | 0.002 |
| Al2Sc | cF24 Cu2Mg | Al | Sc | -48 | 2.8 | 0.333 | 48 | 96 | 16 | 6.00 | 8.00 | -0.572 | 0.007 | -0.489 | 0.007 | -0.572 | 0.017 | -0.041 | 0.002 |
| AlSc | cP2 ClCs_1 | Al | Sc | -46 | 2.7 | 0.5 | 3 | 8 | 3 | 7.00 | 7.00 | -0.490 | 0.007 | -0.559 | 0.007 | -0.584 | 0.019 | -0.060 | 0.004 |
| AlSc2 | hP6 InNi2 | Al | Sc | -37 | 2.2 | 0.667 | 0 | 22 | 14 | 5.50 | 6.25 | -0.624 | 0.007 | -0.626 | 0.007 | -0.660 | 0.018 | -0.035 | 0.002 |
| AlSm2 | oP12 Co2Si | Sm | Al | -38 | 2.3 | 0.333 | 32 | 40 | 0 | 6.50 | 5.00 | -0.330 | 0.004 | -0.686 | 0.007 | -0.547 | 0.014 | -0.039 | 0.002 |
| AlSm | oP16 AlDy | Sm | Al | -49 | 2.9 | 0.5 | 28 | 64 | 12 | 7.50 | 5.50 | -0.286 | 0.004 | -0.624 | 0.007 | -0.518 | 0.015 | -0.063 | 0.004 |
| Al2Sm | cF24 Cu2Mg | Sm | Al | -55 | 3.2 | 0.667 | 16 | 96 | 48 | 8.00 | 6.00 | -0.268 | 0.004 | -0.572 | 0.007 | -0.467 | 0.016 | -0.047 | 0.003 |
| Al3Sm | hP8 Ni3Sn | Sm | Al | -48 | 2.8 | 0.75 | 0 | 24 | 24 | 6.00 | 6.00 | -0.357 | 0.004 | -0.572 | 0.007 | -0.505 | 0.022 | -0.041 | 0.002 |
| Al7Sr8 | cP60 Al7Sr8 | Sr | Al | -21 | 1.2 | 0.467 | 144 | 174 | 36 | 7.22 | 4.39 | -0.235 | 0.003 | -0.781 | 0.007 | -0.513 | 0.014 | -0.005 | 0.000 |
| Al2Sr | oI12 CeCu2 | Sr | Al | -31 | 1.8 | 0.667 | 8 | 48 | 16 | 8.00 | 5.00 | -0.212 | 0.003 | -0.686 | 0.007 | -0.476 | 0.013 | -0.027 | 0.002 |
| Al4Sr | tI10 Al4Ba | Sr | Al | -26 | 1.5 | 0.8 | 4 | 34 | 26 | 10.50 | 5.38 | -0.162 | 0.003 | -0.638 | 0.007 | -0.416 | 0.017 | -0.016 | 0.001 |
| Al3Ta | tI8 Al3Ti | Al | Ta | -26 | 1.5 | 0.25 | 24 | 24 | 0 | 6.00 | 6.00 | -0.572 | 0.007 | -1.351 | 0.004 | -0.984 | 0.021 | -0.022 | 0.001 |
| Al3Ti | tI8 Al3Ti | Al | Ti | -36 | 2.1 | 0.25 | 24 | 24 | 0 | 6.00 | 6.00 | -0.572 | 0.007 | -0.817 | 0.005 | -0.726 | 0.022 | -0.031 | 0.002 |
| Al2Ti | tI24 Ga2Hf | Al | Ti | -41 | 2.4 | 0.333 | 56 | 80 | 10 | 6.00 | 6.25 | -0.572 | 0.007 | -0.784 | 0.023 | -0.720 | 0.041 | -0.042 | 0.002 |
| AlTi | tP4 AuCu | Al | Ti | -40 | 2.4 | 0.5 | 4 | 16 | 4 | 6.00 | 6.00 | -0.572 | 0.007 | -0.817 | 0.005 | -0.746 | 0.015 | -0.052 | 0.003 |
| AlTi3 | hP8 Ni3Sn | Al | Ti | -28 | 1.6 | 0.75 | 0 | 24 | 24 | 6.00 | 6.00 | -0.572 | 0.007 | -0.817 | 0.005 | -0.718 | 0.018 | -0.024 | 0.001 |
| Al2Tm | cF24 Cu2Mg | Tm | Al | -51 | 3.0 | 0.667 | 16 | 96 | 48 | 8.00 | 6.00 | -0.301 | 0.004 | -0.572 | 0.007 | -0.480 | 0.015 | -0.044 | 0.003 |
| Al45V7 | mC104 Al45V7 | Al | V | -15 | 0.9 | 0.135 | 456 | 160 | 4 | 5.96 | 6.00 | -0.576 | 0.007 | -0.890 | 0.012 | -0.735 | 0.051 | -0.002 | 0.000 |
| Al23V4 | hP54 Al23V4 | Al | V | -16 | 0.9 | 0.148 | 228 | 84 | 6 | 5.87 | 6.00 | -0.584 | 0.007 | -0.890 | 0.012 | -0.741 | 0.050 | -0.004 | 0.000 |
| Al3V | tI8 Al3Ti | Al | V | -27 | 1.6 | 0.25 | 24 | 24 | 0 | 6.00 | 6.00 | -0.572 | 0.007 | -0.890 | 0.012 | -0.754 | 0.026 | -0.023 | 0.001 |
| Al8V5 | cI52 Cu5Zn8 | Al | V | -23 | 1.3 | 0.385 | 84 | 192 | 30 | 5.63 | 6.30 | -0.610 | 0.007 | -0.848 | 0.012 | -0.734 | 0.021 | -0.005 | 0.000 |
| Al5W | hP12 Al5W | Al | W | -10 | 0.6 | 0.167 | 48 | 24 | 0 | 6.00 | 6.00 | -0.572 | 0.007 | -1.470 | 0.009 | -1.029 | 0.037 | -0.008 | 0.000 |
| Al4W | mC30 Al4W | Al | W | -12 | 0.7 | 0.2 | 120 | 64 | 4 | 6.33 | 6.00 | -0.542 | 0.007 | -1.470 | 0.009 | -1.017 | 0.036 | -0.011 | 0.001 |
| Al3Y | hP8 Ni3Sn | Al | Y | -48 | 2.8 | 0.25 | 24 | 24 | 0 | 6.00 | 6.00 | -0.572 | 0.007 | -0.734 | 0.004 | -0.694 | 0.022 | -0.041 | 0.002 |
| Al2Y | cF24 Cu2Mg | Al | Y | -50 | 3.0 | 0.333 | 48 | 96 | 16 | 6.00 | 8.00 | -0.572 | 0.007 | -0.550 | 0.004 | -0.604 | 0.015 | -0.044 | 0.003 |
| AlY | oC8 BCr | Al | Y | -45 | 2.7 | 0.5 | 4 | 28 | 20 | 4.50 | 8.50 | -0.762 | 0.007 | -0.518 | 0.004 | -0.707 | 0.017 | -0.067 | 0.004 |
| Al2Y3 | tP20 Al2Zr3 | Al | Y | -40 | 2.4 | 0.6 | 8 | 64 | 59 | 5.00 | 7.58 | -0.686 | 0.007 | -0.580 | 0.038 | -0.659 | 0.092 | -0.026 | 0.002 |
| AlY2 | oP12 Co2Si | Al | Y | -35 | 2.1 | 0.667 | 0 | 40 | 32 | 5.00 | 6.50 | -0.686 | 0.007 | -0.677 | 0.004 | -0.718 | 0.014 | -0.036 | 0.002 |
| Al2Yb | cF24 Cu2Mg | Yb | Al | -33 | 2.0 | 0.667 | 16 | 96 | 48 | 8.00 | 6.00 | -0.202 | 0.004 | -0.572 | 0.007 | -0.415 | 0.014 | -0.029 | 0.002 |
| Al3Yb | cP4 AuCu3_1 | Yb | Al | -33 | 1.9 | 0.75 | 3 | 12 | 12 | 9.00 | 6.00 | -0.179 | 0.004 | -0.572 | 0.007 | -0.403 | 0.022 | -0.028 | 0.002 |
| Al3Zr | tI16 Al3Zr | Al | Zr | -41 | 2.4 | 0.25 | 48 | 48 | 0 | 6.00 | 6.00 | -0.572 | 0.007 | -1.054 | 0.015 | -0.848 | 0.027 | -0.035 | 0.002 |
| Al2Zr | hP12 MgZn2 | Al | Zr | -46 | 2.7 | 0.333 | 24 | 48 | 8 | 6.00 | 8.00 | -0.572 | 0.007 | -0.790 | 0.015 | -0.720 | 0.022 | -0.040 | 0.002 |
| Al3Zr2 | oF40 Al3Zr2 | Al | Zr | -47 | 2.8 | 0.4 | 52 | 148 | 56 | 5.25 | 8.13 | -0.653 | 0.031 | -0.778 | 0.015 | -0.742 | 0.072 | -0.026 | 0.002 |
| AlZr | oC8 BCr | Al | Zr | -45 | 2.6 | 0.5 | 4 | 28 | 20 | 4.50 | 8.50 | -0.762 | 0.007 | -0.744 | 0.015 | -0.819 | 0.034 | -0.066 | 0.004 |
| Al2Zr3 | tP20 Al2Zr3 | Al | Zr | -38 | 2.3 | 0.6 | 8 | 64 | 59 | 5.00 | 7.58 | -0.686 | 0.007 | -0.834 | 0.038 | -0.785 | 0.124 | -0.025 | 0.001 |
| AlZr2 | hP6 InNi2 | Al | Zr | -33 | 2.0 | 0.667 | 0 | 22 | 14 | 5.50 | 6.25 | -0.624 | 0.007 | -1.012 | 0.015 | -0.849 | 0.031 | -0.031 | 0.002 |
| AlZr3 | cP4 AuCu3_2 | Al | Zr | -27 | 1.6 | 0.75 | 0 | 12 | 12 | 6.00 | 6.00 | -0.572 | 0.007 | -1.054 | 0.015 | -0.836 | 0.041 | -0.023 | 0.001 |
| AsCo | oP8 MnP | Co | As | -35 | 2.1 | 0.5 | 12 | 24 | 4 | 6.00 | 4.00 | -0.737 | 0.015 | -0.784 | 0.045 | -0.821 | 0.056 | -0.060 | 0.004 |
| AsFe2 | tP6 Cu2Sb | Fe | As | -11 | 0.7 | 0.333 | 12 | 18 | 0 | 5.25 | 4.50 | -0.820 | 0.002 | -0.697 | 0.045 | -0.771 | 0.020 | -0.013 | 0.001 |
| AsFe | oP8 MnP | Fe | As | -19 | 1.1 | 0.5 | 12 | 24 | 4 | 6.00 | 4.00 | -0.718 | 0.002 | -0.784 | 0.045 | -0.784 | 0.035 | -0.033 | 0.002 |
| As2Fe | oP6 FeS2 | Fe | As | -20 | 1.2 | 0.667 | 0 | 12 | 2 | 3.00 | 2.00 | -1.435 | 0.002 | -1.568 | 0.045 | -1.536 | 0.057 | -0.034 | 0.002 |
| AsGa | cF8 SZn | Ga | As | -44 | 2.6 | 0.5 | 0 | 16 | 0 | 2.00 | 2.00 | -1.409 | 0.006 | -1.568 | 0.045 | -1.604 | 0.046 | -0.115 | 0.007 |
| AsGe | mC24 GaTe | As | Ge | -4 | 0.2 | 0.5 | 0 | 36 | 6 | 1.50 | 2.00 | -2.090 | 0.045 | -1.928 | 0.016 | -2.023 | 0.059 | -0.014 | 0.001 |
| AsIn | cF8 SZn | In | As | -29 | 1.7 | 0.5 | 0 | 16 | 0 | 2.00 | 2.00 | -1.259 | 0.007 | -1.568 | 0.045 | -1.489 | 0.049 | -0.076 | 0.004 |
| AsMn | hP4 AsNi | Mn | As | -29 | 1.7 | 0.5 | 2 | 12 | 0 | 4.00 | 3.00 | -0.734 | 0.007 | -1.045 | 0.045 | -0.940 | 0.034 | -0.050 | 0.003 |
| AsNi | hP4 AsNi | Ni | As | -35 | 2.1 | 0.5 | 2 | 12 | 0 | 4.00 | 3.00 | -1.114 | 0.015 | -1.045 | 0.045 | -1.140 | 0.043 | -0.060 | 0.004 |
| As2Os | oP6 FeS2 | As | Os | -26 | 1.5 | 0.333 | 2 | 12 | 0 | 2.00 | 3.00 | -1.568 | 0.045 | -2.719 | 0.011 | -2.188 | 0.066 | -0.045 | 0.003 |
| Au2Ce | oI12 CeCu2 | Au | Ce | -58 | 4.7 | 0.333 | 16 | 48 | 8 | 5.00 | 8.00 | -0.763 | 0.004 | -0.544 | 0.004 | -0.703 | 0.011 | -0.050 | 0.004 |
| AuCe | oP8 BFe | Au | Ce | -63 | 4.2 | 0.5 | 4 | 28 | 20 | 4.50 | 8.50 | -0.848 | 0.004 | -0.512 | 0.004 | -0.773 | 0.016 | -0.093 | 0.006 |
| Au5Hf | tI10 MoNi4 | Au | Hf | -49 | 2.9 | 0.167 | 36 | 24 | 0 | 5.76 | 7.20 | -0.663 | 0.004 | -0.890 | 0.011 | -0.812 | 0.020 | -0.035 | 0.002 |
| Au4Hf | oP20 Au4Zr | Au | Hf | -51 | 3.0 | 0.2 | 72 | 48 | 0 | 6.00 | 6.00 | -0.636 | 0.004 | -1.068 | 0.011 | -0.896 | 0.021 | -0.044 | 0.003 |
| Au3Hf | oP8 Cu3Ti | Au | Hf | -54 | 3.2 | 0.25 | 24 | 24 | 0 | 6.00 | 6.00 | -0.636 | 0.004 | -1.068 | 0.011 | -0.899 | 0.017 | -0.047 | 0.003 |
| Au2Hf | tI6 MoSi2 | Au | Hf | -59 | 3.5 | 0.333 | 18 | 20 | 4 | 7.00 | 7.00 | -0.545 | 0.004 | -0.916 | 0.011 | -0.792 | 0.020 | -0.062 | 0.004 |
| AuHf | tP4 CuTi | Au | Hf | -61 | 3.6 | 0.5 | 8 | 12 | 8 | 7.00 | 7.00 | -0.545 | 0.004 | -0.916 | 0.011 | -0.836 | 0.030 | -0.106 | 0.006 |
| AuHf2 | tI6 MoSi2 | Au | Hf | -41 | 2.4 | 0.667 | 4 | 20 | 18 | 7.00 | 7.00 | -0.545 | 0.004 | -0.916 | 0.011 | -0.773 | 0.028 | -0.042 | 0.003 |
| AuIn2 | cF12 CaF2 | In | Au | -27 | 1.6 | 0.333 | 24 | 32 | 24 | 5.00 | 10.00 | -0.504 | 0.007 | -0.382 | 0.004 | -0.477 | 0.023 | -0.035 | 0.002 |
| Au3In | oP8 Cu3Ti | In | Au | -11 | 0.7 | 0.75 | 0 | 24 | 24 | 6.00 | 6.00 | -0.420 | 0.007 | -0.636 | 0.004 | -0.537 | 0.013 | -0.010 | 0.001 |
| AuLa | oP8 BFe | Au | La | -70 | 2.3 | 0.5 | 4 | 28 | 20 | 4.50 | 8.50 | -0.848 | 0.004 | -0.526 | 0.004 | -0.790 | 0.013 | -0.103 | 0.003 |
| Au2Nb | hP3 AlB2 | Au | Nb | -13 | 2.0 | 0.333 | 3 | 12 | 4 | 4.50 | 10.00 | -0.848 | 0.004 | -0.760 | 0.012 | -0.815 | 0.017 | -0.012 | 0.002 |
| AuNd | oP8 BFe | Nd | Au | -70 | 3.7 | 0.5 | 20 | 28 | 4 | 8.50 | 4.50 | -0.399 | 0.004 | -0.848 | 0.004 | -0.727 | 0.015 | -0.104 | 0.005 |
| AuPb2 | tI12 Al2Cu | Pb | Au | -3 | 0.2 | 0.333 | 44 | 32 | 4 | 7.50 | 5.00 | -0.270 | 0.001 | -0.763 | 0.004 | -0.520 | 0.007 | -0.004 | 0.000 |
| Au2Pb | cF24 Cu2Mg | Pb | Au | -3 | 0.2 | 0.667 | 16 | 96 | 48 | 8.00 | 6.00 | -0.253 | 0.001 | -0.636 | 0.004 | -0.447 | 0.007 | -0.003 | 0.000 |
| AuPr | oP8 BFe | Pr | Au | -72 | 4.4 | 0.5 | 20 | 28 | 4 | 8.50 | 4.50 | -0.435 | 0.004 | -0.848 | 0.004 | -0.749 | 0.016 | -0.107 | 0.007 |
| Au2Pr | oI12 CeCu2 | Pr | Au | -64 | 3.0 | 0.667 | 16 | 48 | 8 | 10.00 | 4.00 | -0.370 | 0.004 | -0.954 | 0.004 | -0.717 | 0.010 | -0.055 | 0.003 |
| AuTe2 | mC6 AuTe2 | Au | Te | -3 | 0.2 | 0.667 | 0 | 12 | 6 | 3.00 | 3.00 | -1.272 | 0.004 | -0.679 | 0.015 | -0.981 | 0.018 | -0.005 | 0.000 |
| Au4Ti | tI10 MoNi4 | Au | Ti | -40 | 2.4 | 0.2 | 36 | 24 | 0 | 6.00 | 6.00 | -0.636 | 0.004 | -0.817 | 0.005 | -0.761 | 0.017 | -0.034 | 0.002 |
| Au2Ti | tI6 MoSi2 | Au | Ti | -58 | 3.4 | 0.333 | 18 | 20 | 4 | 7.00 | 7.00 | -0.545 | 0.004 | -0.700 | 0.005 | -0.683 | 0.015 | -0.060 | 0.004 |
| AuTi | tP4 CuTi | Au | Ti | -45 | 2.7 | 0.5 | 8 | 12 | 8 | 7.00 | 7.00 | -0.545 | 0.004 | -0.700 | 0.005 | -0.701 | 0.019 | -0.078 | 0.005 |
| AuTi3 | cP8 Cr3Si | Au | Ti | -29 | 1.7 | 0.75 | 0 | 24 | 30 | 6.00 | 7.00 | -0.636 | 0.004 | -0.700 | 0.005 | -0.693 | 0.018 | -0.025 | 0.001 |
| Au4V | tI10 MoNi4 | Au | V | -98 | 2.5 | 0.2 | 36 | 24 | 0 | 6.00 | 6.00 | -0.636 | 0.004 | -0.890 | 0.012 | -0.848 | 0.022 | -0.085 | 0.002 |
| Au2V | oC12 Au2V | Au | V | -10 | 3.5 | 0.333 | 32 | 40 | 6 | 6.50 | 6.50 | -0.587 | 0.045 | -0.822 | 0.045 | -0.715 | 0.159 | -0.011 | 0.004 |
| Au4Zr | oP20 Au4Zr | Au | Zr | -42 | 2.5 | 0.2 | 72 | 48 | 0 | 6.00 | 6.00 | -0.636 | 0.004 | -1.054 | 0.015 | -0.881 | 0.022 | -0.036 | 0.002 |
| Au3Zr | oP8 Cu3Ti | Au | Zr | -51 | 3.0 | 0.25 | 24 | 24 | 0 | 6.00 | 6.00 | -0.636 | 0.004 | -1.054 | 0.015 | -0.889 | 0.019 | -0.044 | 0.003 |
| Au2Zr | tI6 MoSi2 | Au | Zr | -59 | 3.5 | 0.333 | 18 | 20 | 4 | 7.00 | 7.00 | -0.545 | 0.004 | -0.903 | 0.015 | -0.786 | 0.023 | -0.062 | 0.004 |
| AuZr2 | tI6 MoSi2 | Au | Zr | -49 | 2.9 | 0.667 | 4 | 20 | 18 | 7.00 | 7.00 | -0.545 | 0.004 | -0.903 | 0.015 | -0.775 | 0.036 | -0.051 | 0.003 |
| AuZr3 | cP8 Cr3Si | Au | Zr | -37 | 2.2 | 0.75 | 0 | 24 | 30 | 6.00 | 7.00 | -0.636 | 0.004 | -0.903 | 0.015 | -0.801 | 0.044 | -0.032 | 0.002 |
| BCo3 | oP16 CFe3 | Co | B | -15 | 0.9 | 0.25 | 68 | 34 | 2 | 7.08 | 4.75 | -0.624 | 0.015 | -1.233 | 0.101 | -0.947 | 0.202 | -0.018 | 0.002 |
| BCo2 | tI12 Al2Cu | Co | B | -23 | 1.4 | 0.333 | 44 | 32 | 4 | 7.50 | 5.00 | -0.590 | 0.015 | -1.171 | 0.016 | -0.910 | 0.053 | -0.030 | 0.002 |
| BCo | oP8 BFe | Co | B | -32 | 1.9 | 0.5 | 20 | 28 | 4 | 8.50 | 4.50 | -0.520 | 0.015 | -1.301 | 0.016 | -0.958 | 0.036 | -0.047 | 0.003 |
| BCr2 | oF48 BMn4 | Cr | B | -38 | 2.2 | 0.333 | 176 | 128 | 16 | 7.50 | 5.00 | -0.549 | 0.006 | -1.171 | 0.016 | -0.909 | 0.031 | -0.049 | 0.003 |
| B3Cr5 | tI32 B3Cr5 | Cr | B | -40 | 2.4 | 0.375 | 100 | 104 | 4 | 7.60 | 4.67 | -0.542 | 0.014 | -1.255 | 0.016 | -0.914 | 0.050 | -0.016 | 0.001 |
| BCr | oC8 BCr | Cr | B | -48 | 2.8 | 0.5 | 20 | 28 | 4 | 8.50 | 4.50 | -0.485 | 0.006 | -1.301 | 0.016 | -0.963 | 0.025 | -0.070 | 0.004 |
| B2Cr | hP3 AlB2 | Cr | B | -38 | 2.3 | 0.667 | 4 | 12 | 3 | 10.00 | 4.50 | -0.412 | 0.006 | -1.301 | 0.016 | -0.889 | 0.020 | -0.033 | 0.002 |
| BFe2 | tI12 Al2Cu | Fe | B | -27 | 1.6 | 0.333 | 44 | 32 | 4 | 7.50 | 5.00 | -0.574 | 0.002 | -1.171 | 0.016 | -0.907 | 0.017 | -0.035 | 0.002 |
| BFe | oP8 BFe | Fe | B | -36 | 2.1 | 0.5 | 20 | 28 | 4 | 8.50 | 4.50 | -0.507 | 0.002 | -1.301 | 0.016 | -0.957 | 0.016 | -0.053 | 0.003 |
| BHf | cF8 ClNa_2 | Hf | B | -80 | 4.7 | 0.5 | 24 | 24 | 0 | 9.00 | 3.00 | -0.712 | 0.011 | -1.952 | 0.016 | -1.470 | 0.039 | -0.138 | 0.008 |
| B2Hf | hP3 AlB2 | Hf | B | -107 | 6.3 | 0.667 | 4 | 12 | 3 | 10.00 | 4.50 | -0.641 | 0.011 | -1.301 | 0.016 | -1.063 | 0.027 | -0.092 | 0.005 |
| BMn2 | tI12 Al2Cu | Mn | B | -31 | 1.8 | 0.333 | 44 | 32 | 4 | 7.50 | 5.00 | -0.391 | 0.007 | -1.171 | 0.016 | -0.821 | 0.031 | -0.040 | 0.002 |
| BMn | oP8 BFe | Mn | B | -37 | 2.2 | 0.5 | 20 | 28 | 4 | 8.50 | 4.50 | -0.345 | 0.007 | -1.301 | 0.016 | -0.878 | 0.024 | -0.055 | 0.003 |
| BMo2 | tI12 Al2Cu | Mo | B | -47 | 2.8 | 0.333 | 44 | 32 | 4 | 7.50 | 5.00 | -0.911 | 0.006 | -1.171 | 0.016 | -1.101 | 0.030 | -0.061 | 0.004 |
| BMo | tI16 BMo | Mo | B | -58 | 3.5 | 0.5 | 40 | 56 | 8 | 8.50 | 4.50 | -0.804 | 0.006 | -1.301 | 0.016 | -1.139 | 0.024 | -0.086 | 0.005 |
| B5Mo2 | hR7 B5Mo2 | Mo | B | -54 | 3.2 | 0.714 | 7 | 23 | 14 | 9.25 | 5.00 | -0.738 | 0.006 | -1.171 | 0.044 | -0.979 | 0.120 | -0.024 | 0.001 |
| B2Nb | hP3 AlB2 | Nb | B | -72 | 4.2 | 0.667 | 4 | 12 | 3 | 10.00 | 4.50 | -0.760 | 0.012 | -1.301 | 0.016 | -1.092 | 0.027 | -0.062 | 0.004 |
| BNi3 | oP16 CFe3 | Ni | B | -16 | 1.0 | 0.25 | 68 | 34 | 2 | 7.08 | 4.75 | -0.629 | 0.015 | -1.233 | 0.102 | -0.951 | 0.202 | -0.020 | 0.002 |
| BNi2 | tI12 Al2Cu | Ni | B | -21 | 1.3 | 0.333 | 44 | 32 | 4 | 7.50 | 5.00 | -0.594 | 0.015 | -1.171 | 0.016 | -0.910 | 0.052 | -0.028 | 0.002 |
| BNi | oC8 BCr | Ni | B | -19 | 1.1 | 0.5 | 20 | 28 | 4 | 8.50 | 4.50 | -0.524 | 0.015 | -1.301 | 0.016 | -0.941 | 0.034 | -0.028 | 0.002 |
| B2Pd5 | mC28 B2Pd5 | Pd | B | -26 | 1.5 | 0.286 | 112 | 60 | 0 | 7.10 | 3.75 | -0.550 | 0.004 | -1.562 | 0.016 | -1.074 | 0.022 | -0.018 | 0.001 |
| BRe3 | oC16 BRe3 | Re | B | -32 | 1.9 | 0.25 | 68 | 34 | 2 | 7.08 | 4.75 | -1.133 | 0.013 | -1.233 | 0.183 | -1.222 | 0.213 | -0.039 | 0.005 |
| B2Sc | hP3 AlB2 | Sc | B | -102 | 6.1 | 0.667 | 4 | 12 | 3 | 10.00 | 4.50 | -0.392 | 0.007 | -1.301 | 0.016 | -0.935 | 0.024 | -0.088 | 0.005 |
| B3Si | hR14 B6P | B | Si | -6 | 0.4 | 0.25 | 33 | 6 | 1 | 3.43 | 1.14 | -1.708 | 0.016 | -4.081 | 0.041 | -2.933 | 0.236 | -0.039 | 0.002 |
| B2Ta | hP3 AlB2 | Ta | B | -66 | 3.9 | 0.667 | 4 | 12 | 3 | 10.00 | 4.50 | -0.810 | 0.004 | -1.301 | 0.016 | -1.113 | 0.019 | -0.057 | 0.003 |
| BTi | oP8 BFe | Ti | B | -82 | 4.8 | 0.5 | 20 | 28 | 4 | 8.50 | 4.50 | -0.577 | 0.005 | -1.301 | 0.016 | -1.060 | 0.025 | -0.121 | 0.007 |
| B2Ti | hP3 AlB2 | Ti | B | -106 | 6.3 | 0.667 | 4 | 12 | 3 | 10.00 | 4.50 | -0.490 | 0.005 | -1.301 | 0.016 | -0.988 | 0.022 | -0.092 | 0.005 |
| B2V3 | tP10 Si2U3 | V | B | -57 | 3.3 | 0.4 | 32 | 32 | 2 | 8.00 | 4.50 | -0.668 | 0.012 | -1.301 | 0.016 | -1.021 | 0.037 | -0.037 | 0.002 |
| BV | oC8 BCr | V | B | -68 | 4.0 | 0.5 | 20 | 28 | 4 | 8.50 | 4.50 | -0.629 | 0.012 | -1.301 | 0.016 | -1.065 | 0.036 | -0.100 | 0.006 |
| B3V2 | oC8 BCr | V | B | -68 | 4.0 | 0.6 | 20 | 28 | 4 | 10.63 | 3.75 | -0.503 | 0.012 | -1.562 | 0.016 | -1.073 | 0.031 | -0.040 | 0.002 |
| B2V | hP3 AlB2 | V | B | -68 | 4.0 | 0.667 | 4 | 12 | 3 | 10.00 | 4.50 | -0.534 | 0.012 | -1.301 | 0.016 | -0.976 | 0.027 | -0.058 | 0.003 |
| BW2 | tI12 Al2Cu | W | B | -26 | 1.5 | 0.333 | 7 | 23 | 14 | 9.25 | 5.00 | -0.954 | 0.009 | -1.171 | 0.057 | -1.075 | 0.131 | -0.013 | 0.001 |
| BW | tI16 BMo | W | B | -33 | 1.9 | 0.5 | 40 | 56 | 8 | 8.50 | 4.50 | -1.038 | 0.009 | -1.301 | 0.016 | -1.218 | 0.029 | -0.048 | 0.003 |
| B5W2 | hR7 B5Mo2 | W | B | -28 | 1.7 | 0.714 | 44 | 32 | 4 | 7.50 | 5.00 | -1.176 | 0.009 | -1.171 | 0.016 | -1.207 | 0.040 | -0.033 | 0.002 |
| BaCu | hP8 BaCu | Ba | Cu | -3 | 0.2 | 0.5 | 20 | 24 | 6 | 8.00 | 4.50 | -0.232 | 0.007 | -0.777 | 0.002 | -0.509 | 0.018 | -0.005 | 0.000 |
| BaCu13 | cF112 NaZn13 | Ba | Cu | -1 | 0.1 | 0.929 | 0 | 96 | 528 | 6.00 | 5.54 | -0.309 | 0.007 | -0.631 | 0.002 | -0.471 | 0.029 | -0.001 | 0.000 |
| Be17Hf2 | hR19 Be17Nb2 | Be | Hf | -17 | 1.0 | 0.105 | 85 | 38 | 0 | 6.12 | 9.50 | -0.549 | 0.009 | -0.675 | 0.011 | -0.616 | 0.046 | -0.005 | 0.000 |
| Be5Hf | hP6 CaCu5 | Be | Hf | -19 | 1.1 | 0.167 | 21 | 18 | 0 | 6.00 | 9.00 | -0.560 | 0.009 | -0.712 | 0.011 | -0.647 | 0.029 | -0.011 | 0.001 |
| Be2Hf | hP3 AlB2 | Be | Hf | -18 | 1.1 | 0.333 | 3 | 12 | 4 | 4.50 | 10.00 | -0.746 | 0.009 | -0.641 | 0.011 | -0.709 | 0.021 | -0.016 | 0.001 |
| Be12Mo | tI26 Mn12Th | Be | Mo | -8 | 0.5 | 0.077 | 132 | 40 | 0 | 6.33 | 10.00 | -0.530 | 0.009 | -0.683 | 0.006 | -0.611 | 0.062 | -0.004 | 0.000 |
| BeNi | cP2 ClCs_1 | Be | Ni | -42 | 2.5 | 0.5 | 3 | 8 | 3 | 7.00 | 7.00 | -0.480 | 0.009 | -0.637 | 0.015 | -0.613 | 0.029 | -0.054 | 0.003 |
| Be13Y | cF112 NaZn13 | Be | Y | -20 | 1.2 | 0.071 | 528 | 96 | 0 | 5.54 | 6.00 | -0.606 | 0.009 | -0.734 | 0.004 | -0.687 | 0.106 | -0.018 | 0.001 |
| BiCe | cF8 ClNa_1 | Bi | Ce | -115 | 6.8 | 0.5 | 0 | 24 | 0 | 3.00 | 3.00 | -0.724 | 0.005 | -1.451 | 0.004 | -1.286 | 0.019 | -0.198 | 0.012 |
| Bi3Ce4 | cI28 P4Th3 | Bi | Ce | -101 | 6.0 | 0.571 | 48 | 96 | 40 | 8.00 | 5.50 | -0.272 | 0.005 | -0.792 | 0.004 | -0.575 | 0.013 | -0.044 | 0.003 |
| Bi3Ce5 | hP16 Mn5Si3 | Bi | Ce | -92 | 5.4 | 0.625 | 6 | 54 | 46 | 5.50 | 7.30 | -0.395 | 0.005 | -0.596 | 0.004 | -0.531 | 0.012 | -0.035 | 0.002 |
| BiCe2 | tI12 La2Sb | Bi | Ce | -82 | 4.8 | 0.667 | 0 | 36 | 40 | 4.50 | 7.25 | -0.483 | 0.005 | -0.601 | 0.004 | -0.636 | 0.017 | -0.094 | 0.006 |
| BiDy | cF8 ClNa_1 | Bi | Dy | -96 | 5.7 | 0.5 | 0 | 24 | 0 | 3.00 | 3.00 | -0.724 | 0.005 | -1.003 | 0.004 | -1.030 | 0.017 | -0.166 | 0.010 |
| Bi3Dy5 | oP32 Sb3Yb5 | Bi | Dy | -82 | 4.8 | 0.625 | 0 | 106 | 92 | 4.42 | 7.25 | -0.492 | 0.009 | -0.415 | 0.017 | -0.485 | 0.048 | -0.032 | 0.002 |
| BiEr | cF8 ClNa_1 | Bi | Er | -90 | 5.3 | 0.5 | 0 | 24 | 0 | 3.00 | 3.00 | -0.724 | 0.005 | -1.093 | 0.004 | -1.064 | 0.016 | -0.155 | 0.009 |
| BiGd | cF8 ClNa_1 | Bi | Gd | -97 | 5.7 | 0.5 | 0 | 24 | 0 | 3.00 | 3.00 | -0.724 | 0.005 | -1.373 | 0.004 | -1.216 | 0.017 | -0.167 | 0.010 |
| Bi3Gd4 | cI28 P4Th3 | Bi | Gd | -92 | 5.5 | 0.571 | 48 | 96 | 40 | 8.00 | 5.50 | -0.272 | 0.005 | -0.749 | 0.004 | -0.550 | 0.013 | -0.040 | 0.002 |
| Bi3Gd5 | hP16 Mn5Si3 | Bi | Gd | -88 | 5.2 | 0.625 | 6 | 54 | 46 | 5.50 | 7.30 | -0.395 | 0.005 | -0.564 | 0.004 | -0.513 | 0.012 | -0.034 | 0.002 |
| BiIn2 | hP6 InNi2 | In | Bi | 0 | 0.0 | 0.333 | 14 | 22 | 0 | 6.25 | 5.50 | -0.403 | 0.007 | -0.395 | 0.005 | -0.399 | 0.014 | 0.000 | 0.000 |
| Bi3In5 | tI32 B3Cr5 | In | Bi | -1 | 0.0 | 0.375 | 100 | 104 | 4 | 7.60 | 4.67 | -0.331 | 0.009 | -0.466 | 0.005 | -0.399 | 0.032 | 0.000 | 0.000 |
| Bi2K | cF24 Cu2Mg | K | Bi | -29 | 1.7 | 0.667 | 16 | 96 | 48 | 8.00 | 6.00 | -0.115 | 0.001 | -0.362 | 0.005 | -0.264 | 0.008 | -0.025 | 0.001 |
| BiLa | cF8 ClNa_1 | Bi | La | -111 | 6.6 | 0.5 | 0 | 24 | 0 | 3.00 | 3.00 | -0.724 | 0.005 | -1.489 | 0.004 | -1.298 | 0.019 | -0.192 | 0.011 |
| Bi3La4 | cI28 P4Th3 | Bi | La | -103 | 6.1 | 0.571 | 48 | 96 | 40 | 8.00 | 5.50 | -0.272 | 0.005 | -0.812 | 0.004 | -0.586 | 0.013 | -0.045 | 0.003 |
| BiMn | hP4 AsNi | Mn | Bi | -10 | 0.6 | 0.5 | 0 | 12 | 2 | 3.00 | 4.00 | -0.979 | 0.007 | -0.543 | 0.005 | -0.778 | 0.013 | -0.017 | 0.001 |
| BiNa3 | hP8 AsNa3 | Na | Bi | -48 | 4.2 | 0.25 | 20 | 22 | 0 | 5.17 | 5.50 | -0.216 | 0.001 | -0.395 | 0.005 | -0.350 | 0.009 | -0.045 | 0.004 |
| BiNa | tP4 AuCu | Na | Bi | -32 | 1.9 | 0.5 | 4 | 16 | 4 | 6.00 | 6.00 | -0.186 | 0.001 | -0.362 | 0.005 | -0.316 | 0.008 | -0.042 | 0.002 |
| BiNd | cF8 ClNa_1 | Bi | Nd | -111 | 6.6 | 0.5 | 0 | 24 | 0 | 3.00 | 3.00 | -0.724 | 0.005 | -1.129 | 0.004 | -1.119 | 0.019 | -0.192 | 0.011 |
| Bi3Nd4 | cI28 P4Th3 | Bi | Nd | -105 | 6.2 | 0.571 | 48 | 96 | 40 | 8.00 | 5.50 | -0.272 | 0.005 | -0.616 | 0.004 | -0.489 | 0.013 | -0.045 | 0.003 |
| Bi3Nd5 | hP16 Mn5Si3 | Bi | Nd | -92 | 5.5 | 0.625 | 6 | 54 | 46 | 5.50 | 7.30 | -0.395 | 0.005 | -0.464 | 0.004 | -0.465 | 0.012 | -0.035 | 0.002 |
| BiNi | hP4 AsNi | Bi | Ni | -4 | 0.2 | 0.5 | 2 | 12 | 0 | 4.00 | 3.00 | -0.543 | 0.005 | -1.486 | 0.015 | -1.021 | 0.019 | -0.007 | 0.000 |
| BiPd | oC32 BiPd | Bi | Pd | -34 | 2.0 | 0.5 | 70 | 113 | 20 | 7.91 | 4.78 | -0.275 | 0.005 | -0.816 | 0.004 | -0.595 | 0.023 | -0.049 | 0.003 |
| BiPr | cF8 ClNa_1 | Bi | Pr | -100 | 5.9 | 0.5 | 0 | 24 | 0 | 3.00 | 3.00 | -0.724 | 0.005 | -1.233 | 0.004 | -1.151 | 0.017 | -0.173 | 0.010 |
| Bi3Pr4 | cI28 P4Th3 | Bi | Pr | -94 | 5.6 | 0.571 | 48 | 96 | 40 | 8.00 | 5.50 | -0.272 | 0.005 | -0.673 | 0.004 | -0.513 | 0.013 | -0.040 | 0.002 |
| Bi3Sm5 | hP16 Mn5Si3 | Sm | Bi | -94 | 5.6 | 0.375 | 46 | 54 | 6 | 7.30 | 5.50 | -0.293 | 0.004 | -0.395 | 0.005 | -0.380 | 0.012 | -0.036 | 0.002 |
| Bi3Sm4 | cI28 P4Th3 | Sm | Bi | -104 | 6.2 | 0.429 | 40 | 96 | 48 | 5.50 | 8.00 | -0.390 | 0.004 | -0.272 | 0.005 | -0.375 | 0.013 | -0.045 | 0.003 |
| BiSm | cF8 ClNa_1 | Sm | Bi | -108 | 6.4 | 0.5 | 0 | 24 | 0 | 3.00 | 3.00 | -0.714 | 0.004 | -0.724 | 0.005 | -0.906 | 0.018 | -0.187 | 0.011 |
| BiY | cF8 ClNa_1 | Bi | Y | -92 | 5.5 | 0.5 | 0 | 24 | 0 | 3.00 | 3.00 | -0.724 | 0.005 | -1.467 | 0.004 | -1.255 | 0.017 | -0.159 | 0.009 |
| Bi3Y5 | oP32 Bi3Y5 | Bi | Y | -82 | 4.8 | 0.625 | 0 | 104 | 86 | 4.33 | 6.90 | -0.501 | 0.005 | -0.638 | 0.022 | -0.602 | 0.063 | -0.033 | 0.002 |
| C6Cr23 | cF116 C6Cr23 | Cr | C | -11 | 0.7 | 0.207 | 536 | 204 | 0 | 6.93 | 4.25 | -0.594 | 0.006 | -1.748 | 0.003 | -1.173 | 0.037 | -0.002 | 0.000 |
| C3Cr7 | oP40 C3Cr7 | Cr | C | -15 | 0.9 | 0.3 | 156 | 87 | 0 | 7.13 | 3.63 | -0.578 | 0.010 | -2.049 | 0.047 | -1.321 | 0.140 | -0.007 | 0.001 |
| C2Cr3 | oP16 CFe3 | Cr | C | -15 | 0.9 | 0.4 | 68 | 34 | 2 | 8.85 | 2.97 | -0.465 | 0.006 | -2.502 | 0.075 | -1.498 | 0.267 | -0.015 | 0.002 |
| CFe3 | oP16 CFe3 | Fe | C | 6 | 0.3 | 0.25 | 68 | 34 | 2 | 7.08 | 4.75 | -0.608 | 0.007 | -1.564 | 0.098 | -1.079 | 0.182 | 0.007 | 0.000 |
| CHf | cF8 ClNa_2 | Hf | C | -111 | 6.5 | 0.5 | 24 | 24 | 0 | 9.00 | 3.00 | -0.712 | 0.011 | -2.476 | 0.003 | -1.785 | 0.034 | -0.191 | 0.011 |
| C6Mn23 | cF116 C6Cr23 | Mn | C | -11 | 0.6 | 0.207 | 536 | 204 | 0 | 6.93 | 4.25 | -0.423 | 0.007 | -1.748 | 0.003 | -1.088 | 0.038 | -0.002 | 0.000 |
| C3Mn7 | oP40 C3Cr7 | Mn | C | -11 | 0.7 | 0.3 | 156 | 87 | 0 | 7.13 | 3.63 | -0.412 | 0.007 | -2.049 | 0.033 | -1.236 | 0.128 | -0.005 | 0.001 |
| CSi | cF8 SZn | Si | C | -35 | 2.1 | 0.5 | 0 | 16 | 0 | 2.00 | 2.00 | -2.332 | 0.041 | -3.714 | 0.003 | -3.114 | 0.027 | -0.091 | 0.005 |
| CTa | cF8 ClNa_2 | Ta | C | -73 | 4.3 | 0.5 | 24 | 24 | 0 | 9.00 | 3.00 | -0.901 | 0.004 | -2.476 | 0.003 | -1.815 | 0.016 | -0.126 | 0.007 |
| CTi | cF8 ClNa_2 | Ti | C | -93 | 5.5 | 0.5 | 24 | 24 | 0 | 9.00 | 3.00 | -0.545 | 0.005 | -2.476 | 0.003 | -1.671 | 0.021 | -0.161 | 0.010 |
| CV2 | hP4 AsNi | V | C | -49 | 2.9 | 0.333 | 2 | 12 | 0 | 3.00 | 4.50 | -1.781 | 0.012 | -1.651 | 0.003 | -1.772 | 0.024 | -0.056 | 0.003 |
| CV | cF8 ClNa_2 | V | C | -52 | 3.1 | 0.5 | 24 | 24 | 0 | 9.00 | 3.00 | -0.594 | 0.012 | -2.476 | 0.003 | -1.625 | 0.032 | -0.090 | 0.005 |
| CZr | cF8 ClNa_2 | Zr | C | -100 | 5.9 | 0.5 | 24 | 24 | 0 | 9.00 | 3.00 | -0.702 | 0.015 | -2.476 | 0.003 | -1.762 | 0.040 | -0.173 | 0.010 |
| CaCu | mP20 CaCu | Ca | Cu | -8 | 0.5 | 0.5 | 50 | 70 | 14 | 8.50 | 4.90 | -0.217 | 0.001 | -0.714 | 0.002 | -0.477 | 0.005 | -0.012 | 0.001 |
| CaCu5 | hP6 CaCu5 | Ca | Cu | -8 | 0.5 | 0.833 | 0 | 18 | 21 | 9.00 | 6.00 | -0.205 | 0.001 | -0.583 | 0.002 | -0.398 | 0.007 | -0.004 | 0.000 |
| CaLi2 | hP12 MgZn2 | Li | Ca | -4 | 0.2 | 0.333 | 24 | 48 | 8 | 6.00 | 8.00 | -0.275 | 0.001 | -0.230 | 0.001 | -0.256 | 0.004 | -0.003 | 0.000 |
| CaMg2 | hP12 MgZn2 | Mg | Ca | -13 | 0.8 | 0.333 | 24 | 48 | 8 | 6.00 | 8.00 | -0.254 | 0.001 | -0.230 | 0.001 | -0.253 | 0.004 | -0.011 | 0.001 |
| Ca2Si | oP12 Co2Si | Ca | Si | -70 | 4.1 | 0.333 | 32 | 40 | 0 | 6.50 | 5.00 | -0.284 | 0.001 | -0.933 | 0.041 | -0.680 | 0.015 | -0.072 | 0.004 |
| CaSi | oC8 BCr | Ca | Si | -60 | 3.6 | 0.5 | 20 | 28 | 4 | 8.50 | 4.50 | -0.217 | 0.001 | -1.036 | 0.041 | -0.716 | 0.025 | -0.089 | 0.005 |
| CaSi2 | hR6 CaSi2 | Ca | Si | -50 | 3.0 | 0.667 | 6 | 17 | 6 | 7.25 | 3.63 | -0.254 | 0.001 | -1.287 | 0.041 | -0.832 | 0.039 | -0.061 | 0.004 |
| CaZn13 | cF112 NaZn13 | Zn | Ca | -15 | 0.9 | 0.071 | 528 | 96 | 0 | 5.54 | 6.00 | -0.244 | 0.001 | -0.307 | 0.001 | -0.289 | 0.010 | -0.013 | 0.001 |
| CaZn11 | tI48 BaCd11 | Zn | Ca | -18 | 1.0 | 0.083 | 224 | 86 | 0 | 6.07 | 10.75 | -0.223 | 0.001 | -0.171 | 0.001 | -0.206 | 0.005 | -0.008 | 0.001 |
| CaZn5 | hP6 CaCu5 | Zn | Ca | -20 | 1.2 | 0.167 | 21 | 18 | 0 | 6.00 | 9.00 | -0.225 | 0.001 | -0.205 | 0.001 | -0.226 | 0.003 | -0.011 | 0.001 |
| CaZn2 | oI12 CeCu2 | Zn | Ca | -22 | 1.3 | 0.333 | 16 | 48 | 8 | 5.00 | 8.00 | -0.270 | 0.001 | -0.230 | 0.001 | -0.269 | 0.003 | -0.019 | 0.001 |
| CaZn | oC8 BCr | Zn | Ca | -18 | 1.1 | 0.5 | 4 | 28 | 20 | 4.50 | 8.50 | -0.300 | 0.001 | -0.217 | 0.001 | -0.285 | 0.004 | -0.026 | 0.002 |
| Ca5Zn3 | tI32 B3Cr5 | Zn | Ca | -14 | 0.8 | 0.625 | 4 | 104 | 100 | 4.67 | 7.60 | -0.290 | 0.001 | -0.242 | 0.008 | -0.271 | 0.019 | -0.005 | 0.000 |
| Ca3Zn | oC16 BRe3 | Zn | Ca | -9 | 0.5 | 0.75 | 2 | 34 | 68 | 4.75 | 7.08 | -0.285 | 0.046 | -0.260 | 0.003 | -0.283 | 0.047 | -0.011 | 0.001 |
| Cd3Mg | hP8 Ni3Sn | Cd | Mg | -5 | 0.3 | 0.25 | 24 | 24 | 0 | 6.00 | 6.00 | -0.193 | 0.000 | -0.254 | 0.001 | -0.228 | 0.002 | -0.004 | 0.000 |
| CdMg | oP4 AuCd | Cd | Mg | -7 | 0.4 | 0.5 | 4 | 16 | 4 | 6.00 | 6.00 | -0.193 | 0.000 | -0.254 | 0.001 | -0.233 | 0.002 | -0.009 | 0.001 |
| CdMg3 | hP8 Ni3Sn | Cd | Mg | -6 | 0.3 | 0.75 | 0 | 24 | 24 | 6.00 | 6.00 | -0.193 | 0.000 | -0.254 | 0.001 | -0.228 | 0.004 | -0.005 | 0.000 |
| Cd11Na2 | cP39 Mg2Zn11 | Na | Cd | -8 | 0.8 | 0.846 | 3 | 93 | 153 | 8.25 | 6.05 | -0.135 | 0.004 | -0.192 | 0.001 | -0.166 | 0.009 | -0.003 | 0.000 |
| Cd2Pd3 | tP4 AuCu-a | Cd | Pd | -1 | 0.1 | 0.5 | 3 | 14 | 7 | 4.80 | 7.20 | -0.241 | 0.000 | -0.542 | 0.004 | -0.393 | 0.005 | -0.001 | 0.000 |
| Cd46Pd54 | tP4 AuCu-b | Cd | Pd | -1 | 0.0 | 0.5 | 3 | 15 | 5 | 5.52 | 6.48 | -0.210 | 0.000 | -0.602 | 0.004 | -0.407 | 0.005 | -0.001 | 0.000 |
| CdSb | oP16 CdSb | Cd | Sb | -10 | 0.6 | 0.5 | 4 | 32 | 4 | 2.50 | 2.50 | -0.463 | 0.000 | -1.096 | 0.009 | -0.806 | 0.010 | -0.026 | 0.002 |
| CdTe | cF8 ClNa_1 | Cd | Te | -50 | 3.0 | 0.5 | 0 | 24 | 0 | 3.00 | 3.00 | -0.386 | 0.000 | -0.679 | 0.015 | -0.620 | 0.009 | -0.087 | 0.005 |
| Cd58Y13 | hP142 Cd58Gd13 | Cd | Y | -26 | 1.5 | 0.183 | 453 | 403 | 0 | 5.64 | 7.75 | -0.205 | 0.005 | -0.568 | 0.008 | -0.388 | 0.027 | -0.001 | 0.000 |
| Cd2Y | hP3 Cd2Ce | Cd | Y | -38 | 2.2 | 0.333 | 8 | 12 | 1 | 7.00 | 7.00 | -0.166 | 0.000 | -0.629 | 0.004 | -0.430 | 0.005 | -0.033 | 0.002 |
| CdY | cP2 ClCs_1 | Cd | Y | -52 | 3.1 | 0.5 | 3 | 8 | 3 | 7.00 | 7.00 | -0.166 | 0.000 | -0.629 | 0.004 | -0.464 | 0.008 | -0.067 | 0.004 |
| CeCo2 | cF24 Cu2Mg | Ce | Co | -19 | 1.1 | 0.667 | 16 | 96 | 48 | 8.00 | 6.00 | -0.544 | 0.004 | -0.737 | 0.015 | -0.657 | 0.025 | -0.016 | 0.001 |
| CeCo3 | hR12 Be3Nb | Ce | Co | -19 | 1.1 | 0.75 | 5 | 42 | 33 | 8.67 | 6.00 | -0.502 | 0.004 | -0.737 | 0.015 | -0.634 | 0.033 | -0.014 | 0.001 |
| CeCu5 | hP6 CaCu5 | Cu | Ce | -13 | 0.8 | 0.167 | 21 | 18 | 0 | 6.00 | 9.00 | -0.583 | 0.002 | -0.484 | 0.004 | -0.541 | 0.008 | -0.007 | 0.000 |
| CeCu2 | oI12 CeCu2 | Cu | Ce | -21 | 3.0 | 0.333 | 16 | 48 | 8 | 5.00 | 8.00 | -0.699 | 0.002 | -0.544 | 0.004 | -0.640 | 0.008 | -0.018 | 0.003 |
| CeIn3 | cP4 AuCu3_2 | In | Ce | -50 | 3.0 | 0.25 | 12 | 12 | 0 | 6.00 | 6.00 | -0.420 | 0.007 | -0.726 | 0.004 | -0.616 | 0.022 | -0.043 | 0.003 |
| CeIr2 | cF24 Cu2Mg | Ce | Ir | -74 | 2.8 | 0.667 | 16 | 96 | 48 | 8.00 | 6.00 | -0.544 | 0.004 | -1.156 | 0.007 | -0.914 | 0.015 | -0.064 | 0.002 |
| CeMg12 | tI26 Mn12Th | Mg | Ce | -11 | 0.6 | 0.077 | 132 | 40 | 0 | 6.33 | 10.00 | -0.241 | 0.001 | -0.435 | 0.004 | -0.344 | 0.011 | -0.006 | 0.000 |
| Ce5Mg41 | tI92 Ce5Mg41 | Mg | Ce | -13 | 0.7 | 0.109 | 412 | 184 | 0 | 6.15 | 9.20 | -0.248 | 0.002 | -0.473 | 0.011 | -0.362 | 0.025 | -0.001 | 0.000 |
| CeMg3 | cF16 BiF3 | Mg | Ce | -19 | 1.1 | 0.25 | 56 | 56 | 0 | 7.00 | 7.00 | -0.218 | 0.001 | -0.622 | 0.004 | -0.434 | 0.006 | -0.014 | 0.001 |
| CeMg | cP2 ClCs_1 | Mg | Ce | -23 | 1.4 | 0.5 | 3 | 8 | 3 | 7.00 | 7.00 | -0.218 | 0.001 | -0.622 | 0.004 | -0.450 | 0.007 | -0.030 | 0.002 |
| CeNi | oC8 BCr | Ce | Ni | -30 | 1.4 | 0.5 | 20 | 28 | 4 | 8.50 | 4.50 | -0.512 | 0.004 | -0.991 | 0.015 | -0.796 | 0.022 | -0.045 | 0.002 |
| CeNi5 | hP6 CaCu5 | Ce | Ni | -32 | 1.3 | 0.833 | 0 | 18 | 21 | 9.00 | 6.00 | -0.484 | 0.004 | -0.743 | 0.015 | -0.632 | 0.043 | -0.018 | 0.001 |
| CePb3 | cP4 AuCu3_2 | Pb | Ce | -49 | 2.9 | 0.25 | 12 | 12 | 0 | 6.00 | 6.00 | -0.337 | 0.001 | -0.726 | 0.004 | -0.574 | 0.008 | -0.042 | 0.003 |
| CePd3 | cP4 AuCu3_2 | Pd | Ce | -73 | 3.8 | 0.25 | 12 | 12 | 0 | 6.00 | 6.00 | -0.651 | 0.004 | -0.726 | 0.004 | -0.751 | 0.014 | -0.063 | 0.003 |
| CePd | oC8 BCr | Pd | Ce | -78 | 1.5 | 0.5 | 4 | 28 | 20 | 4.50 | 8.50 | -0.867 | 0.004 | -0.512 | 0.004 | -0.806 | 0.012 | -0.116 | 0.002 |
| CePt | oC8 BCr | Ce | Pt | -103 | 4.0 | 0.5 | 20 | 28 | 4 | 8.50 | 4.50 | -0.512 | 0.004 | -1.303 | 0.002 | -1.061 | 0.014 | -0.153 | 0.006 |
| CePt2 | cF24 Cu2Mg | Ce | Pt | -91 | 3.5 | 0.667 | 16 | 96 | 48 | 8.00 | 6.00 | -0.544 | 0.004 | -0.977 | 0.002 | -0.839 | 0.009 | -0.078 | 0.003 |
| Ce5Rh4 | oP36 Ge4Sm5 | Ce | Rh | -71 | 1.9 | 0.444 | 88 | 126 | 6 | 7.55 | 4.31 | -0.577 | 0.004 | -1.336 | 0.025 | -0.980 | 0.050 | -0.023 | 0.001 |
| CeRh2 | cF24 Cu2Mg | Ce | Rh | -67 | 2.3 | 0.667 | 16 | 96 | 48 | 8.00 | 6.00 | -0.544 | 0.004 | -0.960 | 0.007 | -0.810 | 0.015 | -0.057 | 0.002 |
| CeRh3 | cP4 AuCu3_1 | Ce | Rh | -55 | 1.6 | 0.75 | 3 | 12 | 12 | 9.00 | 6.00 | -0.484 | 0.004 | -0.960 | 0.007 | -0.770 | 0.021 | -0.048 | 0.001 |
| CeRu2 | cF24 Cu2Mg | Ce | Ru | -28 | 1.5 | 0.667 | 16 | 96 | 48 | 8.00 | 6.00 | -0.544 | 0.004 | -1.124 | 0.011 | -0.858 | 0.020 | -0.024 | 0.001 |
| Ce2Sb | tI12 La2Sb | Ce | Sb | -103 | 6.1 | 0.333 | 40 | 36 | 0 | 7.25 | 4.50 | -0.601 | 0.004 | -0.609 | 0.009 | -0.723 | 0.019 | -0.118 | 0.007 |
| Ce5Sb3 | hP16 Mn5Si3 | Ce | Sb | -109 | 6.4 | 0.375 | 46 | 54 | 6 | 7.30 | 5.50 | -0.596 | 0.004 | -0.498 | 0.009 | -0.589 | 0.013 | -0.042 | 0.002 |
| Ce4Sb3 | cI28 P4Th3 | Ce | Sb | -117 | 6.9 | 0.429 | 40 | 96 | 48 | 5.50 | 8.00 | -0.792 | 0.004 | -0.343 | 0.009 | -0.617 | 0.016 | -0.050 | 0.003 |
| CeSb | cF8 ClNa_1 | Ce | Sb | -126 | 7.5 | 0.5 | 0 | 24 | 0 | 3.00 | 3.00 | -1.451 | 0.004 | -0.913 | 0.009 | -1.401 | 0.021 | -0.218 | 0.013 |
| CeSb2 | oC24 Sb2Sm | Ce | Sb | -90 | 5.3 | 0.667 | 0 | 72 | 20 | 4.50 | 3.50 | -0.968 | 0.004 | -0.783 | 0.009 | -0.979 | 0.017 | -0.104 | 0.006 |
| CeSi | oP8 BFe | Ce | Si | -71 | 4.2 | 0.5 | 20 | 28 | 4 | 8.50 | 4.50 | -0.512 | 0.004 | -1.036 | 0.041 | -0.880 | 0.030 | -0.105 | 0.006 |
| CeSn3 | cP4 AuCu3_2 | Sn | Ce | -57 | 3.4 | 0.25 | 12 | 12 | 0 | 6.00 | 6.00 | -0.520 | 0.003 | -0.726 | 0.004 | -0.672 | 0.012 | -0.049 | 0.003 |
| Ce3Sn5 | oC32 Pd5Pu3 | Sn | Ce | -75 | 4.5 | 0.375 | 44 | 116 | 37 | 5.10 | 7.92 | -0.612 | 0.003 | -0.550 | 0.059 | -0.608 | 0.069 | -0.027 | 0.002 |
| Ce5Sn4 | oP36 Ge4Sm5 | Sn | Ce | -84 | 5.0 | 0.556 | 6 | 126 | 88 | 4.31 | 7.55 | -0.724 | 0.032 | -0.577 | 0.005 | -0.678 | 0.034 | -0.028 | 0.002 |
| Ce3Sn | cP4 AuCu3_2 | Sn | Ce | -49 | 2.9 | 0.75 | 0 | 12 | 12 | 6.00 | 6.00 | -0.520 | 0.003 | -0.726 | 0.004 | -0.666 | 0.013 | -0.043 | 0.003 |
| CeTl3 | cP4 AuCu3_2 | Tl | Ce | -45 | 2.7 | 0.25 | 12 | 12 | 0 | 6.00 | 6.00 | -0.315 | 0.001 | -0.726 | 0.004 | -0.559 | 0.006 | -0.039 | 0.002 |
| Co2Dy | cF24 Cu2Mg | Dy | Co | -17 | 1.0 | 0.667 | 16 | 96 | 48 | 8.00 | 6.00 | -0.376 | 0.004 | -0.737 | 0.015 | -0.572 | 0.025 | -0.015 | 0.001 |
| Co3Dy | hR12 Be3Nb | Dy | Co | -18 | 1.1 | 0.75 | 5 | 42 | 33 | 8.67 | 6.00 | -0.347 | 0.004 | -0.737 | 0.015 | -0.556 | 0.033 | -0.014 | 0.001 |
| Co17Dy2 | hR19 Th2Zn17 | Dy | Co | -9 | 0.5 | 0.895 | 0 | 38 | 76 | 9.50 | 5.59 | -0.317 | 0.004 | -0.791 | 0.015 | -0.556 | 0.070 | -0.002 | 0.000 |
| CoEr3 | oP16 CFe3 | Er | Co | -7 | 0.4 | 0.25 | 68 | 34 | 2 | 7.08 | 4.75 | -0.463 | 0.005 | -0.931 | 0.075 | -0.706 | 0.130 | -0.009 | 0.001 |
| Co2Er | cF24 Cu2Mg | Er | Co | -19 | 1.1 | 0.667 | 16 | 96 | 48 | 8.00 | 6.00 | -0.410 | 0.004 | -0.737 | 0.015 | -0.590 | 0.025 | -0.016 | 0.001 |
| Co3Er | hR12 Be3Nb | Er | Co | -15 | 0.9 | 0.75 | 5 | 42 | 33 | 8.67 | 6.00 | -0.378 | 0.004 | -0.737 | 0.015 | -0.569 | 0.033 | -0.011 | 0.001 |
| CoGa3 | tP16 FeGa3 | Co | Ga | -45 | 2.7 | 0.75 | 2 | 32 | 64 | 4.50 | 6.67 | -0.983 | 0.015 | -0.423 | 0.006 | -0.761 | 0.036 | -0.058 | 0.003 |
| CoGd3 | oP16 CFe3 | Gd | Co | -10 | 0.6 | 0.25 | 68 | 34 | 2 | 7.08 | 4.75 | -0.582 | 0.007 | -0.931 | 0.094 | -0.768 | 0.138 | -0.012 | 0.001 |
| Co3Gd4 | hP22 Co3Ho4 | Gd | Co | -14 | 0.8 | 0.429 | 53 | 72 | 8 | 7.04 | 4.67 | -0.585 | 0.030 | -0.948 | 0.015 | -0.773 | 0.076 | -0.006 | 0.000 |
| Co2Gd | cF24 Cu2Mg | Gd | Co | -16 | 1.0 | 0.667 | 16 | 96 | 48 | 8.00 | 6.00 | -0.515 | 0.004 | -0.737 | 0.015 | -0.640 | 0.025 | -0.014 | 0.001 |
| Co3Gd | hR12 Be3Nb | Gd | Co | -17 | 1.0 | 0.75 | 5 | 42 | 33 | 8.67 | 6.00 | -0.475 | 0.004 | -0.737 | 0.015 | -0.619 | 0.033 | -0.013 | 0.001 |
| Co7Gd2 | hR18 Co7Er2 | Gd | Co | -16 | 0.9 | 0.778 | 6 | 60 | 54 | 9.00 | 6.00 | -0.458 | 0.004 | -0.737 | 0.015 | -0.603 | 0.036 | -0.006 | 0.000 |
| Co5Gd | hP6 CaCu5 | Gd | Co | -11 | 0.7 | 0.833 | 0 | 18 | 21 | 9.00 | 6.00 | -0.458 | 0.004 | -0.737 | 0.015 | -0.604 | 0.043 | -0.007 | 0.000 |
| Co17Gd2 | hR19 Th2Zn17 | Gd | Co | -7 | 0.4 | 0.895 | 0 | 38 | 76 | 9.50 | 5.59 | -0.434 | 0.004 | -0.791 | 0.015 | -0.614 | 0.070 | -0.002 | 0.000 |
| Co5Ge3 | hP6 InNi2 | Co | Ge | -60 | 3.6 | 0.375 | 14 | 22 | 0 | 6.67 | 4.89 | -0.663 | 0.015 | -0.789 | 0.016 | -0.747 | 0.029 | -0.021 | 0.001 |
| Co5Ge7 | tI24 Co5Ge7 | Co | Ge | -44 | 2.6 | 0.583 | 16 | 64 | 36 | 4.80 | 4.86 | -0.921 | 0.019 | -0.794 | 0.016 | -0.872 | 0.062 | -0.014 | 0.001 |
| CoGe2 | oC24 PdSn2 | Co | Ge | -37 | 2.2 | 0.667 | 4 | 64 | 52 | 4.50 | 5.25 | -0.983 | 0.015 | -0.734 | 0.016 | -0.907 | 0.035 | -0.048 | 0.003 |
| Co2Hf | cF24 Cu2Mg | Co | Hf | -44 | 1.1 | 0.333 | 48 | 96 | 16 | 6.00 | 8.00 | -0.737 | 0.015 | -0.801 | 0.011 | -0.807 | 0.030 | -0.038 | 0.001 |
| CoHf | cP2 ClCs_2 | Co | Hf | -47 | 1.8 | 0.5 | 0 | 8 | 3 | 4.00 | 7.00 | -1.106 | 0.015 | -0.916 | 0.011 | -1.071 | 0.026 | -0.061 | 0.002 |
| CoIn3 | tP16 FeGa3 | In | Co | -2 | 0.1 | 0.25 | 64 | 32 | 2 | 6.67 | 4.50 | -0.378 | 0.007 | -0.983 | 0.015 | -0.683 | 0.041 | -0.002 | 0.000 |
| CoIn2 | oF48 CuMg2 | In | Co | -3 | 0.2 | 0.333 | 176 | 128 | 16 | 7.50 | 5.00 | -0.336 | 0.007 | -0.884 | 0.015 | -0.614 | 0.033 | -0.003 | 0.000 |
| Co5La | hP6 CaCu5 | Co | La | -3 | 0.2 | 0.167 | 21 | 18 | 0 | 6.00 | 9.00 | -0.737 | 0.015 | -0.496 | 0.004 | -0.618 | 0.043 | -0.002 | 0.000 |
| Co3Mo | hP8 Ni3Sn | Co | Mo | -5 | 0.3 | 0.25 | 24 | 24 | 0 | 6.00 | 6.00 | -0.737 | 0.015 | -1.138 | 0.006 | -0.942 | 0.040 | -0.005 | 0.000 |
| Co7Mo6 | hR13 Fe7W6 | Co | Mo | -4 | 0.2 | 0.462 | 18 | 48 | 15 | 6.00 | 6.50 | -0.737 | 0.015 | -1.051 | 0.116 | -0.895 | 0.191 | -0.001 | 0.000 |
| Co2Nb | hP12 MgZn2 | Co | Nb | -19 | 1.1 | 0.333 | 24 | 48 | 8 | 6.00 | 8.00 | -0.737 | 0.015 | -0.950 | 0.012 | -0.860 | 0.032 | -0.016 | 0.001 |
| Co2P | oP12 Co2Si | Co | P | -63 | 3.7 | 0.333 | 32 | 40 | 0 | 6.50 | 5.00 | -0.680 | 0.015 | -0.656 | 0.007 | -0.734 | 0.034 | -0.065 | 0.004 |
| CoP | oP8 MnP | Co | P | -63 | 3.7 | 0.5 | 4 | 24 | 12 | 4.00 | 6.00 | -1.106 | 0.015 | -0.547 | 0.007 | -0.935 | 0.029 | -0.109 | 0.006 |
| CoP3 | cI32 As3Co | Co | P | -51 | 3.0 | 0.75 | 0 | 48 | 24 | 3.00 | 2.00 | -1.474 | 0.015 | -1.640 | 0.007 | -1.645 | 0.029 | -0.088 | 0.005 |
| CoSb | hP4 AsNi | Co | Sb | -21 | 1.2 | 0.5 | 0 | 12 | 2 | 3.00 | 4.00 | -1.474 | 0.015 | -0.685 | 0.009 | -1.116 | 0.023 | -0.036 | 0.002 |
| CoSb3 | cI32 As3Co | Co | Sb | -16 | 1.0 | 0.75 | 0 | 48 | 24 | 3.00 | 2.00 | -1.474 | 0.015 | -1.370 | 0.009 | -1.450 | 0.034 | -0.028 | 0.002 |
| CoSc2 | tI12 Al2Cu | Sc | Co | -27 | 1.9 | 0.333 | 44 | 32 | 4 | 7.50 | 5.00 | -0.522 | 0.007 | -0.884 | 0.015 | -0.738 | 0.035 | -0.035 | 0.002 |
| Co2Sc | cF24 Cu2Mg | Sc | Co | -33 | 2.1 | 0.667 | 16 | 96 | 48 | 8.00 | 6.00 | -0.489 | 0.007 | -0.737 | 0.015 | -0.641 | 0.029 | -0.028 | 0.002 |
| Co2Si | oP12 Co2Si | Co | Si | -36 | 2.1 | 0.333 | 32 | 40 | 0 | 6.50 | 5.00 | -0.680 | 0.015 | -0.933 | 0.041 | -0.844 | 0.040 | -0.037 | 0.002 |
| CoSi | cP8 FeSi | Co | Si | -45 | 2.7 | 0.5 | 12 | 28 | 12 | 6.50 | 6.50 | -0.680 | 0.015 | -0.718 | 0.041 | -0.766 | 0.053 | -0.067 | 0.004 |
| CoSi2 | cF12 CaF2 | Co | Si | -31 | 1.9 | 0.667 | 24 | 32 | 24 | 10.00 | 5.00 | -0.442 | 0.015 | -0.933 | 0.041 | -0.728 | 0.076 | -0.041 | 0.002 |
| Co7Sm2 | hR18 Co7Er2 | Sm | Co | -11 | 0.7 | 0.778 | 6 | 60 | 54 | 9.00 | 6.00 | -0.238 | 0.004 | -0.737 | 0.015 | -0.491 | 0.036 | -0.004 | 0.000 |
| Co17Sm2 | hR19 Th2Zn17 | Sm | Co | -8 | 0.5 | 0.895 | 0 | 38 | 76 | 9.50 | 5.59 | -0.226 | 0.004 | -0.791 | 0.015 | -0.511 | 0.070 | -0.002 | 0.000 |
| Co3Ti | cP4 AuCu3_2 | Co | Ti | -21 | 1.2 | 0.25 | 12 | 12 | 0 | 6.00 | 6.00 | -0.737 | 0.015 | -0.817 | 0.005 | -0.795 | 0.041 | -0.018 | 0.001 |
| Co2Ti | cF24 Cu2Mg | Co | Ti | -28 | 1.7 | 0.333 | 48 | 96 | 16 | 6.00 | 8.00 | -0.737 | 0.015 | -0.613 | 0.005 | -0.699 | 0.027 | -0.024 | 0.001 |
| CoTi | cP2 ClCs_1 | Co | Ti | -43 | 2.6 | 0.5 | 3 | 8 | 3 | 7.00 | 7.00 | -0.632 | 0.015 | -0.700 | 0.005 | -0.722 | 0.026 | -0.056 | 0.003 |
| CoTi2 | cF96 NiTi2 | Co | Ti | -34 | 2.0 | 0.667 | 48 | 288 | 288 | 6.00 | 6.75 | -0.737 | 0.015 | -0.726 | 0.005 | -0.771 | 0.027 | -0.039 | 0.002 |
| Co3V | hP24 Co3V | Co | V | -10 | 0.6 | 0.25 | 72 | 72 | 0 | 6.00 | 6.00 | -0.737 | 0.015 | -0.890 | 0.012 | -0.822 | 0.044 | -0.009 | 0.001 |
| CoV3 | cP8 Cr3Si | Co | V | -11 | 0.6 | 0.75 | 0 | 24 | 30 | 6.00 | 7.00 | -0.737 | 0.015 | -0.763 | 0.012 | -0.760 | 0.043 | -0.009 | 0.001 |
| Co3W | hP8 Ni3Sn | Co | W | 2 | 0.1 | 0.25 | 24 | 24 | 0 | 6.00 | 6.00 | -0.737 | 0.015 | -1.470 | 0.009 | -1.101 | 0.042 | 0.002 | 0.000 |
| Co7W6 | hR13 Fe7W6 | Co | W | -2 | 0.1 | 0.462 | 18 | 48 | 15 | 6.00 | 6.50 | -0.737 | 0.015 | -1.357 | 0.116 | -1.047 | 0.233 | 0.000 | 0.000 |
| CoY3 | oP16 CFe3 | Y | Co | -19 | 1.1 | 0.25 | 68 | 34 | 2 | 7.08 | 4.75 | -0.621 | 0.007 | -0.931 | 0.101 | -0.799 | 0.142 | -0.023 | 0.003 |
| Co5Y8 | mP52 Co5Y8 | Y | Co | -29 | 1.7 | 0.385 | 172 | 145 | 14 | 7.64 | 4.33 | -0.576 | 0.006 | -1.023 | 0.017 | -0.808 | 0.054 | -0.008 | 0.001 |
| CoY | oC8 BCr | Y | Co | -36 | 2.1 | 0.5 | 20 | 28 | 4 | 8.50 | 4.50 | -0.518 | 0.004 | -0.983 | 0.015 | -0.803 | 0.024 | -0.053 | 0.003 |
| Co2Y | cF24 Cu2Mg | Y | Co | -28 | 1.6 | 0.667 | 16 | 96 | 48 | 8.00 | 6.00 | -0.550 | 0.004 | -0.737 | 0.015 | -0.668 | 0.026 | -0.024 | 0.001 |
| Co7Y2 | hR18 Co7Er2 | Y | Co | -20 | 1.2 | 0.778 | 6 | 60 | 54 | 9.00 | 6.00 | -0.489 | 0.004 | -0.737 | 0.015 | -0.620 | 0.036 | -0.007 | 0.000 |
| Co5Y | hP6 CaCu5 | Y | Co | -10 | 0.6 | 0.833 | 0 | 18 | 21 | 9.00 | 6.00 | -0.489 | 0.004 | -0.737 | 0.015 | -0.619 | 0.043 | -0.006 | 0.000 |
| Co17Y2 | hR19 Th2Zn17 | Y | Co | -10 | 0.6 | 0.895 | 0 | 38 | 76 | 9.50 | 5.59 | -0.463 | 0.004 | -0.791 | 0.015 | -0.630 | 0.070 | -0.003 | 0.000 |
| CoZn13 | mC28 CoZn13 | Zn | Co | -6 | 0.4 | 0.071 | 150 | 24 | 0 | 6.23 | 6.00 | -0.217 | 0.005 | -0.737 | 0.015 | -0.482 | 0.082 | -0.005 | 0.000 |
| Co2Zr | cF24 Cu2Mg | Co | Zr | -38 | 1.1 | 0.333 | 48 | 96 | 16 | 6.00 | 8.00 | -0.737 | 0.015 | -0.790 | 0.015 | -0.797 | 0.033 | -0.033 | 0.001 |
| CoZr | cP2 ClCs_2 | Co | Zr | -41 | 0.9 | 0.5 | 0 | 8 | 3 | 4.00 | 7.00 | -1.106 | 0.015 | -0.903 | 0.015 | -1.058 | 0.028 | -0.053 | 0.001 |
| CoZr2 | tI12 Al2Cu | Co | Zr | -30 | 1.5 | 0.667 | 4 | 32 | 44 | 5.00 | 7.50 | -0.884 | 0.015 | -0.843 | 0.015 | -0.902 | 0.057 | -0.038 | 0.002 |
| CrGe | cP8 FeSi | Cr | Ge | -10 | 0.6 | 0.5 | 12 | 28 | 12 | 6.50 | 6.50 | -0.634 | 0.006 | -0.593 | 0.016 | -0.628 | 0.021 | -0.015 | 0.001 |
| Cr2Hf | hP12 MgZn2 | Cr | Hf | -2 | 0.1 | 0.333 | 24 | 48 | 8 | 6.00 | 8.00 | -0.687 | 0.006 | -0.801 | 0.011 | -0.745 | 0.018 | -0.002 | 0.000 |
| Cr2Nb | cF24 Cu2Mg | Cr | Nb | -6 | 0.3 | 0.333 | 48 | 96 | 16 | 6.00 | 8.00 | -0.687 | 0.006 | -0.950 | 0.012 | -0.823 | 0.020 | -0.005 | 0.000 |
| Cr3P | tI32 Ni3P | Cr | P | -40 | 2.4 | 0.25 | 128 | 72 | 0 | 6.83 | 4.50 | -0.603 | 0.006 | -0.729 | 0.007 | -0.712 | 0.029 | -0.046 | 0.003 |
| Cr3Pt | cP8 Cr3Si | Cr | Pt | -23 | 1.4 | 0.25 | 30 | 24 | 0 | 7.00 | 6.00 | -0.589 | 0.006 | -0.977 | 0.002 | -0.803 | 0.021 | -0.020 | 0.001 |
| Cr2Ru | tP30 CrFe | Cr | Ru | -3 | 0.2 | 0.333 | 51 | 104 | 46 | 5.17 | 9.87 | -0.798 | 0.006 | -0.683 | 0.011 | -0.743 | 0.023 | -0.003 | 0.000 |
| CrSb2 | oP6 FeS2 | Cr | Sb | -6 | 0.4 | 0.667 | 0 | 12 | 2 | 3.00 | 2.00 | -1.373 | 0.006 | -1.370 | 0.009 | -1.382 | 0.018 | -0.011 | 0.001 |
| Cr3Si | cP8 Cr3Si | Cr | Si | -32 | 1.9 | 0.25 | 30 | 24 | 0 | 7.00 | 6.00 | -0.589 | 0.006 | -0.777 | 0.041 | -0.710 | 0.027 | -0.027 | 0.002 |
| Cr5Si3 | tI32 Si3W5 | Cr | Si | -32 | 1.9 | 0.375 | 92 | 112 | 4 | 7.40 | 5.00 | -0.557 | 0.006 | -0.933 | 0.041 | -0.757 | 0.024 | -0.012 | 0.001 |
| CrSi | cP8 FeSi | Cr | Si | -30 | 1.8 | 0.5 | 12 | 28 | 12 | 6.50 | 6.50 | -0.634 | 0.006 | -0.718 | 0.041 | -0.720 | 0.041 | -0.045 | 0.003 |
| CrSi2 | hP9 CrSi2 | Cr | Si | -26 | 1.5 | 0.667 | 6 | 30 | 27 | 7.00 | 7.00 | -0.589 | 0.006 | -0.666 | 0.041 | -0.654 | 0.061 | -0.027 | 0.002 |
| Cr2Ta | cF24 Cu2Mg | Cr | Ta | -9 | 0.5 | 0.333 | 48 | 96 | 16 | 6.00 | 8.00 | -0.687 | 0.006 | -1.013 | 0.004 | -0.858 | 0.014 | -0.008 | 0.000 |
| Cr2Ti | cF24 Cu2Mg | Cr | Ti | -1 | 0.0 | 0.333 | 48 | 96 | 16 | 6.00 | 8.00 | -0.687 | 0.006 | -0.613 | 0.005 | -0.650 | 0.014 | -0.001 | 0.000 |
| Cr2Zr | cF24 Cu2Mg | Cr | Zr | -29 | 1.7 | 0.333 | 48 | 96 | 16 | 6.00 | 8.00 | -0.687 | 0.006 | -0.790 | 0.015 | -0.764 | 0.022 | -0.025 | 0.001 |
| CuDy | cP2 ClCs_2 | Dy | Cu | -13 | 0.7 | 0.5 | 3 | 8 | 0 | 7.00 | 4.00 | -0.430 | 0.004 | -0.874 | 0.002 | -0.668 | 0.007 | -0.016 | 0.001 |
| Cu2Dy | oI12 CeCu2 | Dy | Cu | -16 | 1.0 | 0.667 | 8 | 48 | 16 | 8.00 | 5.00 | -0.376 | 0.004 | -0.699 | 0.002 | -0.552 | 0.006 | -0.014 | 0.001 |
| CuEr | cP2 ClCs_2 | Er | Cu | -15 | 0.9 | 0.5 | 3 | 8 | 0 | 7.00 | 4.00 | -0.468 | 0.004 | -0.874 | 0.002 | -0.690 | 0.007 | -0.019 | 0.001 |
| Cu2Er | oI12 CeCu2 | Er | Cu | -17 | 1.0 | 0.667 | 8 | 48 | 16 | 8.00 | 5.00 | -0.410 | 0.004 | -0.699 | 0.002 | -0.569 | 0.006 | -0.015 | 0.001 |
| Cu5Er | cF24 AuBe5 | Er | Cu | -11 | 0.6 | 0.833 | 0 | 64 | 96 | 8.00 | 6.40 | -0.410 | 0.004 | -0.546 | 0.002 | -0.485 | 0.009 | -0.007 | 0.000 |
| Cu2Gd | oI12 CeCu2 | Cu | Gd | -14 | 0.8 | 0.333 | 16 | 48 | 8 | 5.00 | 8.00 | -0.699 | 0.002 | -0.515 | 0.004 | -0.619 | 0.006 | -0.012 | 0.001 |
| CuGd | cP2 ClCs_2 | Cu | Gd | -11 | 0.6 | 0.5 | 0 | 8 | 3 | 4.00 | 7.00 | -0.874 | 0.002 | -0.589 | 0.004 | -0.745 | 0.006 | -0.014 | 0.001 |
| Cu10Hf7 | oC68 Ni10Zr7 | Cu | Hf | -4 | 0.2 | 0.412 | 84 | 254 | 88 | 5.28 | 7.68 | -0.663 | 0.016 | -0.835 | 0.015 | -0.749 | 0.049 | -0.001 | 0.000 |
| CuHf2 | tI6 MoSi2 | Cu | Hf | -15 | 0.9 | 0.667 | 4 | 20 | 18 | 7.00 | 7.00 | -0.500 | 0.002 | -0.916 | 0.011 | -0.723 | 0.025 | -0.016 | 0.001 |
| Cu6La | oP28 CeCu6 | Cu | La | -11 | 1.8 | 0.143 | 110 | 76 | 0 | 6.17 | 9.50 | -0.567 | 0.008 | -0.470 | 0.004 | -0.524 | 0.032 | -0.006 | 0.001 |
| Cu5La | hP6 CaCu5 | Cu | La | -12 | 0.7 | 0.167 | 21 | 18 | 0 | 6.00 | 9.00 | -0.583 | 0.002 | -0.496 | 0.004 | -0.547 | 0.008 | -0.007 | 0.000 |
| Cu2La | hP3 AlB2 | Cu | La | -16 | 1.6 | 0.333 | 3 | 12 | 4 | 4.50 | 10.00 | -0.777 | 0.002 | -0.447 | 0.004 | -0.626 | 0.007 | -0.014 | 0.001 |
| CuLa | oP8 BFe | Cu | La | -16 | 0.9 | 0.5 | 4 | 28 | 20 | 4.50 | 8.50 | -0.777 | 0.002 | -0.526 | 0.004 | -0.675 | 0.009 | -0.023 | 0.001 |
| CuMg2 | oF48 CuMg2 | Mg | Cu | -10 | 0.6 | 0.333 | 176 | 128 | 16 | 7.50 | 5.00 | -0.203 | 0.001 | -0.699 | 0.002 | -0.464 | 0.007 | -0.012 | 0.001 |
| Cu2Mg | cF24 Cu2Mg | Mg | Cu | -11 | 0.6 | 0.667 | 16 | 96 | 48 | 8.00 | 6.00 | -0.191 | 0.001 | -0.583 | 0.002 | -0.396 | 0.005 | -0.009 | 0.001 |
| Cu2Nd | oI12 CeCu2 | Nd | Cu | -17 | 2.7 | 0.667 | 8 | 48 | 16 | 8.00 | 5.00 | -0.424 | 0.004 | -0.699 | 0.002 | -0.576 | 0.008 | -0.015 | 0.002 |
| Cu6Nd | oP28 CeCu6 | Nd | Cu | -13 | 3.1 | 0.857 | 0 | 76 | 110 | 9.50 | 6.17 | -0.357 | 0.004 | -0.567 | 0.005 | -0.469 | 0.029 | -0.007 | 0.002 |
| Cu6Pr | oP28 CeCu6 | Cu | Pr | -11 | 2.7 | 0.143 | 110 | 76 | 0 | 6.17 | 9.50 | -0.567 | 0.008 | -0.389 | 0.004 | -0.484 | 0.033 | -0.006 | 0.001 |
| Cu2Pr | oI12 CeCu2 | Cu | Pr | -15 | 2.6 | 0.333 | 16 | 48 | 8 | 5.00 | 8.00 | -0.699 | 0.002 | -0.462 | 0.004 | -0.593 | 0.007 | -0.013 | 0.002 |
| Cu2Sb | tP6 Cu2Sb | Cu | Sb | -4 | 0.3 | 0.333 | 12 | 18 | 0 | 5.25 | 4.50 | -0.666 | 0.002 | -0.609 | 0.009 | -0.643 | 0.007 | -0.005 | 0.000 |
| Cu2Sc | tI6 MoSi2 | Cu | Sc | -17 | 1.1 | 0.333 | 18 | 20 | 4 | 7.00 | 7.00 | -0.500 | 0.002 | -0.559 | 0.007 | -0.547 | 0.011 | -0.018 | 0.001 |
| CuSc | cP2 ClCs_2 | Cu | Sc | -21 | 1.3 | 0.5 | 0 | 8 | 3 | 4.00 | 7.00 | -0.874 | 0.002 | -0.559 | 0.007 | -0.744 | 0.011 | -0.027 | 0.002 |
| Cu5Sm | hP6 CaCu5 | Sm | Cu | -14 | 0.8 | 0.833 | 0 | 18 | 21 | 9.00 | 6.00 | -0.238 | 0.004 | -0.583 | 0.002 | -0.418 | 0.008 | -0.008 | 0.000 |
| Cu6Sm | oP28 CeCu6 | Sm | Cu | -11 | 0.7 | 0.857 | 0 | 76 | 110 | 9.50 | 6.17 | -0.226 | 0.004 | -0.567 | 0.003 | -0.402 | 0.025 | -0.006 | 0.000 |
| Cu3Sn | oP8 Cu3Ti | Cu | Sn | -8 | 0.5 | 0.25 | 24 | 24 | 0 | 6.00 | 6.00 | -0.583 | 0.002 | -0.520 | 0.003 | -0.559 | 0.007 | -0.007 | 0.000 |
| CuSr | hP8 BaCu | Sr | Cu | -2 | 0.1 | 0.5 | 20 | 24 | 6 | 8.00 | 4.50 | -0.212 | 0.003 | -0.777 | 0.002 | -0.498 | 0.008 | -0.003 | 0.000 |
| Cu5Sr | hP6 CaCu5 | Sr | Cu | -2 | 0.1 | 0.833 | 0 | 18 | 21 | 9.00 | 6.00 | -0.189 | 0.003 | -0.583 | 0.002 | -0.387 | 0.007 | -0.001 | 0.000 |
| Cu4Ti | oP20 Au4Zr | Cu | Ti | -6 | 0.4 | 0.2 | 72 | 48 | 0 | 6.00 | 6.00 | -0.583 | 0.002 | -0.817 | 0.005 | -0.705 | 0.010 | -0.005 | 0.000 |
| Cu3Ti2 | tP10 Cu3Ti2 | Cu | Ti | -9 | 0.5 | 0.4 | 26 | 32 | 12 | 7.00 | 7.00 | -0.500 | 0.002 | -0.700 | 0.005 | -0.606 | 0.010 | -0.006 | 0.000 |
| Cu4Ti3 | tI14 Cu4Ti3 | Cu | Ti | -10 | 0.6 | 0.429 | 34 | 44 | 20 | 7.00 | 7.00 | -0.500 | 0.002 | -0.700 | 0.005 | -0.605 | 0.011 | -0.005 | 0.000 |
| CuTi | tP4 CuTi | Cu | Ti | -11 | 0.7 | 0.5 | 8 | 12 | 8 | 7.00 | 7.00 | -0.500 | 0.002 | -0.700 | 0.005 | -0.619 | 0.013 | -0.019 | 0.001 |
| CuTi2 | tI6 MoSi2 | Cu | Ti | -12 | 0.7 | 0.667 | 4 | 20 | 18 | 7.00 | 7.00 | -0.500 | 0.002 | -0.700 | 0.005 | -0.613 | 0.013 | -0.013 | 0.001 |
| Cu2Y | oI12 CeCu2 | Cu | Y | -20 | 1.2 | 0.333 | 16 | 48 | 8 | 5.00 | 8.00 | -0.699 | 0.002 | -0.550 | 0.004 | -0.642 | 0.006 | -0.017 | 0.001 |
| CuY | cP2 ClCs_2 | Cu | Y | -22 | 1.3 | 0.5 | 0 | 8 | 3 | 4.00 | 7.00 | -0.874 | 0.002 | -0.629 | 0.004 | -0.780 | 0.007 | -0.029 | 0.002 |
| Cu5Zn8 | cI52 Cu5Zn8 | Zn | Cu | -12 | 0.7 | 0.385 | 84 | 192 | 30 | 5.63 | 6.30 | -0.240 | 0.001 | -0.555 | 0.002 | -0.400 | 0.003 | -0.002 | 0.000 |
| CuZn | cP2 ClCs_1 | Zn | Cu | -9 | 0.6 | 0.5 | 3 | 8 | 3 | 7.00 | 7.00 | -0.193 | 0.001 | -0.500 | 0.002 | -0.359 | 0.004 | -0.012 | 0.001 |
| Cu5Zr | cF24 AuBe5 | Cu | Zr | -10 | 0.6 | 0.167 | 96 | 64 | 0 | 6.40 | 8.00 | -0.546 | 0.002 | -0.790 | 0.015 | -0.675 | 0.013 | -0.007 | 0.000 |
| Cu8Zr3 | oP44 Cu8Hf3 | Cu | Zr | -13 | 0.8 | 0.273 | 120 | 148 | 24 | 6.06 | 8.17 | -0.577 | 0.002 | -0.774 | 0.015 | -0.679 | 0.014 | -0.004 | 0.000 |
| Cu10Zr7 | oC68 Ni10Zr7 | Cu | Zr | -14 | 0.8 | 0.412 | 84 | 254 | 88 | 5.28 | 7.68 | -0.663 | 0.016 | -0.823 | 0.015 | -0.745 | 0.052 | -0.002 | 0.000 |
| CuZr2 | tI6 MoSi2 | Cu | Zr | -15 | 0.9 | 0.667 | 4 | 20 | 18 | 7.00 | 7.00 | -0.500 | 0.002 | -0.903 | 0.015 | -0.717 | 0.033 | -0.015 | 0.001 |
| DyIn3 | cP4 AuCu3_2 | In | Dy | -45 | 2.7 | 0.25 | 12 | 12 | 0 | 6.00 | 6.00 | -0.420 | 0.007 | -0.502 | 0.004 | -0.499 | 0.021 | -0.039 | 0.002 |
| DyMg2 | hP12 MgZn2 | Mg | Dy | -14 | 0.8 | 0.333 | 24 | 48 | 8 | 6.00 | 8.00 | -0.254 | 0.001 | -0.376 | 0.004 | -0.327 | 0.005 | -0.012 | 0.001 |
| DyMg | cP2 ClCs_1 | Mg | Dy | -14 | 0.8 | 0.5 | 3 | 8 | 3 | 7.00 | 7.00 | -0.218 | 0.001 | -0.430 | 0.004 | -0.342 | 0.007 | -0.018 | 0.001 |
| Dy3Ni | oP16 CFe3 | Dy | Ni | -22 | 1.3 | 0.25 | 68 | 34 | 2 | 7.08 | 4.75 | -0.425 | 0.005 | -0.938 | 0.069 | -0.709 | 0.131 | -0.027 | 0.003 |
| DyNi | oP8 BFe | Dy | Ni | -34 | 1.7 | 0.5 | 20 | 28 | 4 | 8.50 | 4.50 | -0.354 | 0.004 | -0.991 | 0.015 | -0.722 | 0.023 | -0.050 | 0.003 |
| DyNi2 | cF24 Cu2Mg | Dy | Ni | -33 | 1.9 | 0.667 | 16 | 96 | 48 | 8.00 | 6.00 | -0.376 | 0.004 | -0.743 | 0.015 | -0.588 | 0.026 | -0.028 | 0.002 |
| DyNi5 | hP6 CaCu5 | Dy | Ni | -26 | 0.8 | 0.833 | 0 | 18 | 21 | 9.00 | 6.00 | -0.334 | 0.004 | -0.743 | 0.015 | -0.553 | 0.043 | -0.015 | 0.000 |
| DyPb3 | cP4 AuCu3_2 | Pb | Dy | -43 | 2.5 | 0.25 | 12 | 12 | 0 | 6.00 | 6.00 | -0.337 | 0.001 | -0.502 | 0.004 | -0.456 | 0.007 | -0.037 | 0.002 |
| DyPd | oP8 BFe | Dy | Pd | -72 | 2.0 | 0.5 | 20 | 28 | 4 | 8.50 | 4.50 | -0.354 | 0.004 | -0.867 | 0.004 | -0.718 | 0.012 | -0.107 | 0.003 |
| Dy3Pd4 | hR14 Pd4Pu3 | Dy | Pd | -86 | 2.1 | 0.571 | 21 | 54 | 20 | 8.00 | 5.88 | -0.376 | 0.004 | -0.664 | 0.024 | -0.553 | 0.054 | -0.033 | 0.001 |
| DyPd3 | cP4 AuCu3_2 | Dy | Pd | -76 | 1.5 | 0.75 | 0 | 12 | 12 | 6.00 | 6.00 | -0.502 | 0.004 | -0.651 | 0.004 | -0.642 | 0.012 | -0.066 | 0.001 |
| DyPt | oP8 BFe | Dy | Pt | -97 | 1.8 | 0.5 | 20 | 28 | 4 | 8.50 | 4.50 | -0.354 | 0.004 | -1.303 | 0.002 | -0.973 | 0.011 | -0.144 | 0.003 |
| DyPt2 | cF24 Cu2Mg | Dy | Pt | -98 | 2.8 | 0.667 | 16 | 96 | 48 | 8.00 | 6.00 | -0.376 | 0.004 | -0.977 | 0.002 | -0.761 | 0.008 | -0.085 | 0.002 |
| DyPt3 | cP4 AuCu3_2 | Dy | Pt | -83 | 2.2 | 0.75 | 0 | 12 | 12 | 6.00 | 6.00 | -0.502 | 0.004 | -0.977 | 0.002 | -0.811 | 0.009 | -0.072 | 0.002 |
| Dy7Rh3 | hP20 Fe3Th7 | Dy | Rh | -57 | 2.2 | 0.3 | 78 | 53 | 0 | 7.45 | 4.38 | -0.404 | 0.004 | -1.317 | 0.012 | -0.883 | 0.042 | -0.022 | 0.002 |
| DyRh | cP2 ClCs_2 | Dy | Rh | -77 | 2.0 | 0.5 | 3 | 8 | 0 | 7.00 | 4.00 | -0.430 | 0.004 | -1.441 | 0.007 | -1.034 | 0.012 | -0.099 | 0.003 |
| DyRh2 | cF24 Cu2Mg | Dy | Rh | -62 | 0.8 | 0.667 | 16 | 96 | 48 | 8.00 | 6.00 | -0.376 | 0.004 | -0.960 | 0.007 | -0.722 | 0.013 | -0.054 | 0.001 |
| DyRu2 | hP12 MgZn2 | Dy | Ru | -27 | 0.9 | 0.667 | 8 | 48 | 24 | 8.00 | 6.00 | -0.376 | 0.004 | -1.124 | 0.011 | -0.774 | 0.020 | -0.024 | 0.001 |
| Dy5Sb3 | hP16 Mn5Si3 | Dy | Sb | -106 | 6.3 | 0.375 | 46 | 54 | 6 | 7.30 | 5.50 | -0.412 | 0.004 | -0.498 | 0.009 | -0.496 | 0.013 | -0.040 | 0.002 |
| Dy4Sb3 | cI28 P4Th3 | Dy | Sb | -112 | 6.6 | 0.429 | 40 | 96 | 48 | 5.50 | 8.00 | -0.547 | 0.004 | -0.343 | 0.009 | -0.493 | 0.016 | -0.048 | 0.003 |
| DySb | cF8 ClNa_1 | Dy | Sb | -114 | 6.8 | 0.5 | 0 | 24 | 0 | 3.00 | 3.00 | -1.003 | 0.004 | -0.913 | 0.009 | -1.155 | 0.020 | -0.197 | 0.012 |
| DyTl3 | cP4 AuCu3_2 | Tl | Dy | -34 | 2.0 | 0.25 | 12 | 12 | 0 | 6.00 | 6.00 | -0.315 | 0.001 | -0.502 | 0.004 | -0.438 | 0.005 | -0.029 | 0.002 |
| ErIn3 | cP4 AuCu3_2 | In | Er | -39 | 2.3 | 0.25 | 12 | 12 | 0 | 6.00 | 6.00 | -0.420 | 0.007 | -0.547 | 0.004 | -0.517 | 0.021 | -0.034 | 0.002 |
| ErIr | cP2 ClCs_2 | Er | Ir | -83 | 2.1 | 0.5 | 3 | 8 | 0 | 7.00 | 4.00 | -0.468 | 0.004 | -1.733 | 0.007 | -1.208 | 0.012 | -0.107 | 0.003 |
| ErIr2 | cF24 Cu2Mg | Er | Ir | -76 | 2.2 | 0.667 | 16 | 96 | 48 | 8.00 | 6.00 | -0.410 | 0.004 | -1.156 | 0.007 | -0.848 | 0.015 | -0.066 | 0.002 |
| Er3Ni | oP16 CFe3 | Er | Ni | -17 | 1.0 | 0.25 | 68 | 34 | 2 | 7.08 | 4.75 | -0.463 | 0.005 | -0.938 | 0.075 | -0.722 | 0.132 | -0.021 | 0.002 |
| ErNi | oP8 BFe | Er | Ni | -36 | 1.5 | 0.5 | 20 | 28 | 4 | 8.50 | 4.50 | -0.386 | 0.004 | -0.991 | 0.015 | -0.742 | 0.022 | -0.054 | 0.002 |
| ErNi2 | cF24 Cu2Mg | Er | Ni | -31 | 1.9 | 0.667 | 16 | 96 | 48 | 8.00 | 6.00 | -0.410 | 0.004 | -0.743 | 0.015 | -0.603 | 0.026 | -0.027 | 0.002 |
| ErNi5 | hP6 CaCu5 | Er | Ni | -25 | 0.7 | 0.833 | 0 | 18 | 21 | 9.00 | 6.00 | -0.364 | 0.004 | -0.743 | 0.015 | -0.568 | 0.043 | -0.014 | 0.000 |
| Er2Ni17 | hP38 Ni17Th2 | Er | Ni | -13 | 0.8 | 0.895 | 0 | 76 | 170 | 9.50 | 6.12 | -0.345 | 0.004 | -0.729 | 0.015 | -0.540 | 0.130 | -0.004 | 0.000 |
| ErPd | cP2 ClCs_2 | Er | Pd | -75 | 2.2 | 0.5 | 3 | 8 | 0 | 7.00 | 4.00 | -0.468 | 0.004 | -0.976 | 0.004 | -0.820 | 0.010 | -0.098 | 0.003 |
| Er3Pd4 | hR14 Pd4Pu3 | Er | Pd | -95 | 1.8 | 0.571 | 21 | 54 | 20 | 8.00 | 5.88 | -0.410 | 0.004 | -0.664 | 0.026 | -0.573 | 0.054 | -0.036 | 0.001 |
| ErPd3 | cP4 AuCu3_2 | Er | Pd | -75 | 1.4 | 0.75 | 0 | 12 | 12 | 6.00 | 6.00 | -0.547 | 0.004 | -0.651 | 0.004 | -0.663 | 0.012 | -0.065 | 0.001 |
| ErPt | oP8 BFe | Er | Pt | -100 | 2.3 | 0.5 | 20 | 28 | 4 | 8.50 | 4.50 | -0.386 | 0.004 | -1.303 | 0.002 | -0.992 | 0.011 | -0.148 | 0.003 |
| ErPt2 | cF24 Cu2Mg | Er | Pt | -108 | 2.8 | 0.667 | 16 | 96 | 48 | 8.00 | 6.00 | -0.410 | 0.004 | -0.977 | 0.002 | -0.787 | 0.008 | -0.093 | 0.002 |
| ErPt3 | cP4 AuCu3_2 | Er | Pt | -94 | 1.8 | 0.75 | 0 | 12 | 12 | 6.00 | 6.00 | -0.547 | 0.004 | -0.977 | 0.002 | -0.843 | 0.009 | -0.081 | 0.002 |
| ErRh | cP2 ClCs_2 | Er | Rh | -87 | 1.7 | 0.5 | 3 | 8 | 0 | 7.00 | 4.00 | -0.468 | 0.004 | -1.441 | 0.007 | -1.068 | 0.011 | -0.113 | 0.002 |
| ErRh2 | cF24 Cu2Mg | Er | Rh | -70 | 2.0 | 0.667 | 16 | 96 | 48 | 8.00 | 6.00 | -0.410 | 0.004 | -0.960 | 0.007 | -0.745 | 0.015 | -0.060 | 0.002 |
| ErRu2 | hP12 MgZn2 | Er | Ru | -26 | 1.3 | 0.667 | 8 | 48 | 24 | 8.00 | 6.00 | -0.410 | 0.004 | -1.124 | 0.011 | -0.789 | 0.020 | -0.023 | 0.001 |
| EuPb3 | cP4 AuCu3_2 | Eu | Pb | -44 | 2.6 | 0.75 | 0 | 12 | 12 | 6.00 | 6.00 | -0.306 | 0.003 | -0.337 | 0.001 | -0.360 | 0.008 | -0.038 | 0.002 |
| Eu5Pd2 | mC28 B2Pd5 | Eu | Pd | -45 | 2.7 | 0.286 | 112 | 60 | 0 | 7.10 | 3.75 | -0.259 | 0.003 | -1.041 | 0.004 | -0.681 | 0.018 | -0.031 | 0.002 |
| Eu3Pd2 | hR15 Er3Ni2 | Eu | Pd | -63 | 3.7 | 0.4 | 47 | 48 | 3 | 7.89 | 4.50 | -0.233 | 0.003 | -0.867 | 0.004 | -0.591 | 0.012 | -0.041 | 0.002 |
| EuPd | oC8 BCr | Eu | Pd | -79 | 4.7 | 0.5 | 20 | 28 | 4 | 8.50 | 4.50 | -0.216 | 0.003 | -0.867 | 0.004 | -0.658 | 0.016 | -0.116 | 0.007 |
| EuPd2 | cF24 Cu2Mg | Eu | Pd | -105 | 6.2 | 0.667 | 16 | 96 | 48 | 8.00 | 6.00 | -0.230 | 0.003 | -0.651 | 0.004 | -0.531 | 0.013 | -0.090 | 0.005 |
| EuPd3 | cP4 AuCu3_1 | Eu | Pd | -95 | 5.7 | 0.75 | 3 | 12 | 12 | 9.00 | 6.00 | -0.204 | 0.003 | -0.651 | 0.004 | -0.510 | 0.017 | -0.082 | 0.005 |
| Fe6Ga5 | mC44 Fe6Ge5 | Fe | Ga | -41 | 2.5 | 0.455 | 68 | 150 | 66 | 5.96 | 7.05 | -0.723 | 0.035 | -0.400 | 0.006 | -0.573 | 0.061 | -0.011 | 0.001 |
| FeGa3 | tP16 FeGa3 | Fe | Ga | -20 | 1.2 | 0.75 | 2 | 32 | 64 | 4.50 | 6.67 | -0.957 | 0.002 | -0.423 | 0.006 | -0.716 | 0.024 | -0.026 | 0.002 |
| Fe17Gd2 | hR19 Th2Zn17 | Fe | Gd | -2 | 0.1 | 0.105 | 76 | 38 | 0 | 5.59 | 9.50 | -0.771 | 0.002 | -0.434 | 0.004 | -0.603 | 0.011 | -0.001 | 0.000 |
| Fe3Gd | hR12 Be3Nb | Fe | Gd | -9 | 0.6 | 0.25 | 33 | 42 | 5 | 6.00 | 8.67 | -0.718 | 0.002 | -0.475 | 0.004 | -0.603 | 0.007 | -0.007 | 0.000 |
| Fe2Gd | cF24 Cu2Mg | Fe | Gd | -12 | 0.7 | 0.333 | 48 | 96 | 16 | 6.00 | 8.00 | -0.718 | 0.002 | -0.515 | 0.004 | -0.626 | 0.006 | -0.010 | 0.001 |
| Fe2N | hP9 NV2 | Fe | N | -1 | 0.1 | 0.333 | 0 | 18 | 0 | 1.49 | 2.98 | -2.890 | 0.002 | -1.644 | 0.004 | -2.269 | 0.005 | -0.002 | 0.000 |
| Fe2Nb | hP12 MgZn2 | Fe | Nb | -13 | 0.7 | 0.333 | 24 | 48 | 8 | 6.00 | 8.00 | -0.718 | 0.002 | -0.950 | 0.012 | -0.845 | 0.013 | -0.011 | 0.001 |
| Fe17Nd2 | hR19 Th2Zn17 | Nd | Fe | -1 | 0.0 | 0.895 | 0 | 38 | 76 | 9.50 | 5.59 | -0.357 | 0.004 | -0.771 | 0.002 | -0.564 | 0.011 | 0.000 | 0.000 |
| FeNi3 | cP4 AuCu3_2 | Fe | Ni | -14 | 0.8 | 0.75 | 0 | 12 | 12 | 6.00 | 6.00 | -0.718 | 0.002 | -0.743 | 0.015 | -0.742 | 0.038 | -0.012 | 0.001 |
| Fe3P | tI32 Ni3P | Fe | P | -41 | 2.4 | 0.25 | 128 | 72 | 0 | 6.83 | 4.50 | -0.630 | 0.002 | -0.729 | 0.007 | -0.727 | 0.012 | -0.047 | 0.003 |
| Fe2P | hP9 Fe2P | Fe | P | -50 | 2.9 | 0.333 | 29 | 28 | 0 | 7.06 | 4.63 | -0.610 | 0.027 | -0.709 | 0.016 | -0.715 | 0.087 | -0.056 | 0.005 |
| FeP | oP8 MnP | Fe | P | -59 | 3.5 | 0.5 | 12 | 24 | 4 | 6.00 | 4.00 | -0.718 | 0.002 | -0.820 | 0.007 | -0.871 | 0.012 | -0.102 | 0.006 |
| FeP2 | oP6 FeS2 | Fe | P | -74 | 4.4 | 0.667 | 0 | 12 | 2 | 3.00 | 2.00 | -1.435 | 0.002 | -1.640 | 0.007 | -1.666 | 0.014 | -0.128 | 0.008 |
| Fe17Pr2 | hR19 Th2Zn17 | Fe | Pr | 0 | 0.0 | 0.105 | 76 | 38 | 0 | 5.59 | 9.50 | -0.771 | 0.002 | -0.389 | 0.004 | -0.580 | 0.011 | 0.000 | 0.000 |
| Fe2Pu | cF24 Cu2Mg | Pu | Fe | -13 | 0.8 | 0.667 | 16 | 96 | 48 | 8.00 | 6.00 | -0.447 | 0.010 | -0.718 | 0.002 | -0.594 | 0.012 | -0.011 | 0.001 |
| FeS | hP24 FeS | Fe | S | -49 | 2.9 | 0.5 | 24 | 72 | 0 | 5.00 | 3.00 | -0.861 | 0.002 | -0.958 | 0.002 | -0.994 | 0.008 | -0.085 | 0.005 |
| FeS2 | oP6 FeS2 | Fe | S | -56 | 3.3 | 0.667 | 0 | 12 | 2 | 3.00 | 2.00 | -1.435 | 0.002 | -1.436 | 0.002 | -1.533 | 0.009 | -0.097 | 0.006 |
| Fe2Sc | hP12 MgZn2 | Fe | Sc | -11 | 1.2 | 0.333 | 24 | 48 | 8 | 6.00 | 8.00 | -0.718 | 0.002 | -0.489 | 0.007 | -0.613 | 0.009 | -0.010 | 0.001 |
| FeSi | cP8 FeSi | Fe | Si | -32 | 1.9 | 0.5 | 12 | 28 | 12 | 6.50 | 6.50 | -0.663 | 0.002 | -0.718 | 0.041 | -0.737 | 0.035 | -0.047 | 0.003 |
| FeSi2 | oC48 FeSi2 | Fe | Si | -24 | 1.4 | 0.667 | 16 | 128 | 80 | 5.00 | 4.50 | -0.861 | 0.002 | -1.036 | 0.041 | -0.980 | 0.050 | -0.032 | 0.002 |
| FeSn | hP6 CoSn | Fe | Sn | -19 | 1.2 | 0.5 | 6 | 18 | 6 | 5.00 | 5.00 | -0.861 | 0.002 | -0.624 | 0.179 | -0.776 | 0.171 | -0.034 | 0.002 |
| FeSn2 | tI12 Al2Cu | Fe | Sn | -23 | 1.4 | 0.667 | 4 | 32 | 44 | 5.00 | 7.50 | -0.861 | 0.002 | -0.416 | 0.003 | -0.668 | 0.012 | -0.030 | 0.002 |
| Fe2Ta | hP12 MgZn2 | Fe | Ta | -19 | 1.1 | 0.333 | 24 | 48 | 8 | 6.00 | 8.00 | -0.718 | 0.002 | -1.013 | 0.004 | -0.882 | 0.007 | -0.016 | 0.001 |
| Fe2Ti | hP12 MgZn2 | Fe | Ti | -19 | 1.1 | 0.333 | 24 | 48 | 8 | 6.00 | 8.00 | -0.718 | 0.002 | -0.613 | 0.005 | -0.682 | 0.008 | -0.017 | 0.001 |
| FeTi | cP2 ClCs_1 | Fe | Ti | -25 | 1.5 | 0.5 | 3 | 8 | 3 | 7.00 | 7.00 | -0.615 | 0.002 | -0.700 | 0.005 | -0.690 | 0.010 | -0.032 | 0.002 |
| Fe3Y | hR12 Be3Nb | Fe | Y | -9 | 0.5 | 0.25 | 33 | 42 | 5 | 6.00 | 8.67 | -0.718 | 0.002 | -0.508 | 0.004 | -0.619 | 0.007 | -0.007 | 0.000 |
| FeZn13 | mC28 CoZn13 | Zn | Fe | -2 | 0.1 | 0.071 | 150 | 24 | 0 | 6.23 | 6.00 | -0.217 | 0.005 | -0.718 | 0.002 | -0.469 | 0.075 | -0.002 | 0.000 |
| Fe11Zn40 | cF408 Fe11Zn39 | Zn | Fe | -6 | 0.4 | 0.216 | 1632 | 720 | 168 | 6.23 | 6.00 | -0.217 | 0.001 | -0.718 | 0.002 | -0.468 | 0.006 | -0.001 | 0.000 |
| Fe3Zn7 | cI52 Cu5Zn8 | Zn | Fe | -7 | 0.4 | 0.3 | 84 | 192 | 30 | 4.95 | 8.08 | -0.273 | 0.001 | -0.533 | 0.002 | -0.405 | 0.003 | -0.002 | 0.000 |
| Fe2Zr | cF24 Cu2Mg | Fe | Zr | -24 | 1.4 | 0.333 | 48 | 96 | 16 | 6.00 | 8.00 | -0.718 | 0.002 | -0.790 | 0.015 | -0.774 | 0.014 | -0.020 | 0.001 |
| FeZr3 | oC16 BRe3 | Fe | Zr | -15 | 0.9 | 0.75 | 2 | 34 | 68 | 4.75 | 7.08 | -0.907 | 0.147 | -0.893 | 0.015 | -0.918 | 0.179 | -0.019 | 0.002 |
| Ga2Mg5 | oI28 Ga2Mg5 | Mg | Ga | -11 | 0.6 | 0.286 | 96 | 84 | 0 | 6.90 | 5.25 | -0.221 | 0.003 | -0.537 | 0.011 | -0.384 | 0.026 | -0.005 | 0.001 |
| GaMg | tI32 GaMg | Mg | Ga | -13 | 0.8 | 0.5 | 64 | 108 | 24 | 7.38 | 4.88 | -0.207 | 0.004 | -0.578 | 0.006 | -0.413 | 0.024 | -0.020 | 0.002 |
| Ga2Mg | oP24 Ga2Mg | Mg | Ga | -10 | 0.6 | 0.667 | 22 | 86 | 36 | 8.13 | 4.94 | -0.188 | 0.003 | -0.571 | 0.006 | -0.389 | 0.018 | -0.010 | 0.001 |
| Ga5Mg2 | tI28 Ga5Mg2 | Mg | Ga | -10 | 0.6 | 0.714 | 4 | 80 | 42 | 5.50 | 4.10 | -0.277 | 0.025 | -0.687 | 0.006 | -0.487 | 0.025 | -0.005 | 0.000 |
| GaNd3 | cP4 AuCu3_2 | Nd | Ga | -41 | 2.4 | 0.25 | 12 | 12 | 0 | 6.00 | 6.00 | -0.565 | 0.004 | -0.470 | 0.006 | -0.553 | 0.013 | -0.035 | 0.002 |
| Ga3Nd5 | tI32 B3Cr5 | Nd | Ga | -55 | 3.3 | 0.375 | 100 | 104 | 4 | 7.60 | 4.67 | -0.446 | 0.012 | -0.604 | 0.006 | -0.547 | 0.037 | -0.022 | 0.001 |
| GaNd | oC8 BCr | Nd | Ga | -73 | 4.3 | 0.5 | 20 | 28 | 4 | 8.50 | 4.50 | -0.399 | 0.004 | -0.626 | 0.006 | -0.621 | 0.016 | -0.108 | 0.006 |
| Ga2Nd | hP3 AlB2 | Nd | Ga | -83 | 4.9 | 0.667 | 3 | 12 | 4 | 9.00 | 5.00 | -0.376 | 0.004 | -0.564 | 0.006 | -0.542 | 0.013 | -0.072 | 0.004 |
| GaNi3 | cP4 AuCu3_2 | Ni | Ga | -26 | 1.5 | 0.25 | 12 | 12 | 0 | 6.00 | 6.00 | -0.743 | 0.015 | -0.470 | 0.006 | -0.628 | 0.039 | -0.022 | 0.001 |
| Ga2Ni3 | hP4 AsNi | Ni | Ga | -41 | 2.4 | 0.4 | 0 | 12 | 2 | 2.50 | 5.00 | -1.783 | 0.015 | -0.564 | 0.006 | -1.201 | 0.023 | -0.028 | 0.002 |
| GaNi | cP2 ClCs_1 | Ni | Ga | -43 | 2.5 | 0.5 | 3 | 8 | 3 | 7.00 | 7.00 | -0.637 | 0.015 | -0.403 | 0.006 | -0.575 | 0.025 | -0.055 | 0.003 |
| Ga3Ni2 | hP5 Al3Ni2 | Ni | Ga | -45 | 2.7 | 0.6 | 3 | 16 | 9 | 5.50 | 5.67 | -0.810 | 0.015 | -0.497 | 0.006 | -0.683 | 0.023 | -0.029 | 0.002 |
| GaP | cF8 SZn | Ga | P | -57 | 3.4 | 0.5 | 0 | 16 | 0 | 2.00 | 2.00 | -1.409 | 0.006 | -1.640 | 0.007 | -1.673 | 0.017 | -0.149 | 0.009 |
| GaSb | cF8 SZn | Ga | Sb | -22 | 1.3 | 0.5 | 0 | 16 | 0 | 2.00 | 2.00 | -1.409 | 0.006 | -1.370 | 0.009 | -1.448 | 0.015 | -0.058 | 0.003 |
| GaTe | mC24 GaTe | Ga | Te | -39 | 2.3 | 0.5 | 6 | 36 | 0 | 2.00 | 1.50 | -1.409 | 0.006 | -1.358 | 0.015 | -1.519 | 0.024 | -0.135 | 0.008 |
| Ga2Te3 | cF8 SZn | Ga | Te | -38 | 2.2 | 0.6 | 0 | 16 | 0 | 2.50 | 1.67 | -1.127 | 0.006 | -1.223 | 0.015 | -1.214 | 0.013 | -0.039 | 0.002 |
| GaTi2 | hP6 InNi2 | Ga | Ti | -39 | 2.3 | 0.667 | 0 | 22 | 14 | 5.50 | 6.25 | -0.512 | 0.006 | -0.784 | 0.005 | -0.685 | 0.013 | -0.036 | 0.002 |
| GaTi3 | hP8 Ni3Sn | Ga | Ti | -34 | 2.0 | 0.75 | 0 | 24 | 24 | 6.00 | 6.00 | -0.470 | 0.006 | -0.817 | 0.005 | -0.673 | 0.017 | -0.029 | 0.002 |
| GaY | oC8 BCr | Y | Ga | -71 | 4.2 | 0.5 | 20 | 28 | 4 | 8.50 | 4.50 | -0.518 | 0.004 | -0.626 | 0.006 | -0.677 | 0.016 | -0.105 | 0.006 |
| Ga2Y | hP3 AlB2 | Y | Ga | -69 | 4.1 | 0.667 | 4 | 12 | 3 | 10.00 | 4.50 | -0.440 | 0.004 | -0.626 | 0.006 | -0.593 | 0.012 | -0.060 | 0.004 |
| GdIn3 | cP4 AuCu3_2 | In | Gd | -46 | 2.7 | 0.25 | 12 | 12 | 0 | 6.00 | 6.00 | -0.420 | 0.007 | -0.687 | 0.004 | -0.593 | 0.021 | -0.039 | 0.002 |
| GdIr2 | cF24 Cu2Mg | Gd | Ir | -69 | 2.2 | 0.667 | 16 | 96 | 48 | 8.00 | 6.00 | -0.515 | 0.004 | -1.156 | 0.007 | -0.894 | 0.015 | -0.059 | 0.002 |
| GdMg5 | cF448 Cd45Sm11 | Mg | Gd | -9 | 0.5 | 0.167 | 1560 | 1294 | 0 | 5.91 | 8.66 | -0.258 | 0.015 | -0.476 | 0.004 | -0.372 | 0.049 | -0.005 | 0.000 |
| GdMg3 | cF16 BiF3 | Mg | Gd | -12 | 0.7 | 0.25 | 56 | 56 | 0 | 7.00 | 7.00 | -0.218 | 0.001 | -0.589 | 0.004 | -0.412 | 0.005 | -0.009 | 0.001 |
| GdMg2 | cF24 Cu2Mg | Mg | Gd | -15 | 0.9 | 0.333 | 48 | 96 | 16 | 6.00 | 8.00 | -0.254 | 0.001 | -0.515 | 0.004 | -0.397 | 0.005 | -0.013 | 0.001 |
| GdMg | cP2 ClCs_1 | Mg | Gd | -15 | 0.9 | 0.5 | 3 | 8 | 3 | 7.00 | 7.00 | -0.218 | 0.001 | -0.589 | 0.004 | -0.423 | 0.007 | -0.020 | 0.001 |
| Gd6Mn23 | cF116 Mn23Th6 | Mn | Gd | -6 | 0.4 | 0.207 | 416 | 306 | 48 | 6.18 | 8.38 | -0.475 | 0.007 | -0.492 | 0.007 | -0.484 | 0.034 | -0.001 | 0.000 |
| GdMn2 | cF24 Cu2Mg | Mn | Gd | -6 | 0.4 | 0.333 | 48 | 96 | 16 | 6.00 | 8.00 | -0.489 | 0.007 | -0.515 | 0.004 | -0.508 | 0.013 | -0.006 | 0.000 |
| Gd3Ni | oP16 CFe3 | Gd | Ni | -16 | 1.0 | 0.25 | 68 | 34 | 2 | 7.08 | 4.75 | -0.582 | 0.007 | -0.938 | 0.094 | -0.780 | 0.140 | -0.020 | 0.002 |
| GdNi | oC8 BCr | Gd | Ni | -31 | 1.5 | 0.5 | 20 | 28 | 4 | 8.50 | 4.50 | -0.485 | 0.004 | -0.991 | 0.015 | -0.783 | 0.022 | -0.046 | 0.002 |
| GdNi2 | cF24 Cu2Mg | Gd | Ni | -35 | 2.1 | 0.667 | 16 | 96 | 48 | 8.00 | 6.00 | -0.515 | 0.004 | -0.743 | 0.015 | -0.659 | 0.026 | -0.030 | 0.002 |
| GdNi3 | hR12 Be3Nb | Gd | Ni | -38 | 2.2 | 0.75 | 5 | 42 | 33 | 8.67 | 6.00 | -0.475 | 0.004 | -0.743 | 0.015 | -0.637 | 0.034 | -0.028 | 0.002 |
| Gd2Ni7 | hP36 Ce2Ni7 | Gd | Ni | -35 | 2.1 | 0.778 | 11 | 120 | 108 | 8.88 | 6.00 | -0.464 | 0.007 | -0.743 | 0.015 | -0.616 | 0.040 | -0.012 | 0.001 |
| GdNi5 | hP6 CaCu5 | Gd | Ni | -27 | 1.0 | 0.833 | 0 | 18 | 21 | 9.00 | 6.00 | -0.458 | 0.004 | -0.743 | 0.015 | -0.616 | 0.043 | -0.016 | 0.001 |
| Gd2Ni17 | hP38 Ni17Th2 | Gd | Ni | -20 | 1.2 | 0.895 | 0 | 76 | 170 | 9.50 | 6.12 | -0.434 | 0.004 | -0.729 | 0.015 | -0.587 | 0.130 | -0.005 | 0.000 |
| GdPb3 | cP4 AuCu3_2 | Pb | Gd | -45 | 2.7 | 0.25 | 12 | 12 | 0 | 6.00 | 6.00 | -0.337 | 0.001 | -0.687 | 0.004 | -0.551 | 0.008 | -0.039 | 0.002 |
| GdPd3 | cP4 AuCu3_2 | Pd | Gd | -87 | 2.7 | 0.25 | 12 | 12 | 0 | 6.00 | 6.00 | -0.651 | 0.004 | -0.687 | 0.004 | -0.744 | 0.013 | -0.075 | 0.002 |
| Gd3Pd4 | hR14 Pd4Pu3 | Pd | Gd | -88 | 2.9 | 0.429 | 20 | 54 | 21 | 5.88 | 8.00 | -0.664 | 0.043 | -0.515 | 0.004 | -0.624 | 0.061 | -0.034 | 0.001 |
| GdPd | oC8 BCr | Pd | Gd | -79 | 1.1 | 0.5 | 4 | 28 | 20 | 4.50 | 8.50 | -0.867 | 0.004 | -0.485 | 0.004 | -0.793 | 0.011 | -0.117 | 0.002 |
| Gd7Pd3 | hP20 Fe3Th7 | Pd | Gd | -53 | 3.1 | 0.7 | 0 | 53 | 78 | 4.38 | 7.45 | -0.892 | 0.026 | -0.553 | 0.006 | -0.744 | 0.040 | -0.021 | 0.002 |
| Gd7Pt3 | hP20 Fe3Th7 | Gd | Pt | -73 | 4.3 | 0.3 | 78 | 53 | 0 | 7.45 | 4.38 | -0.553 | 0.004 | -1.340 | 0.016 | -0.975 | 0.043 | -0.029 | 0.003 |
| Gd2Pt | oP12 Co2Si | Gd | Pt | -77 | 4.6 | 0.333 | 32 | 40 | 0 | 6.50 | 5.00 | -0.634 | 0.004 | -1.173 | 0.002 | -0.983 | 0.013 | -0.080 | 0.005 |
| GdPt | oP8 BFe | Gd | Pt | -99 | 2.8 | 0.5 | 20 | 28 | 4 | 8.50 | 4.50 | -0.485 | 0.004 | -1.303 | 0.002 | -1.041 | 0.012 | -0.147 | 0.004 |
| Gd3Pt4 | hR14 Pd4Pu3 | Gd | Pt | -96 | 5.7 | 0.571 | 21 | 54 | 20 | 8.00 | 5.88 | -0.515 | 0.004 | -0.998 | 0.033 | -0.793 | 0.076 | -0.037 | 0.002 |
| GdPt2 | cF24 Cu2Mg | Gd | Pt | -96 | 2.6 | 0.667 | 16 | 96 | 48 | 8.00 | 6.00 | -0.515 | 0.004 | -0.977 | 0.002 | -0.829 | 0.008 | -0.083 | 0.002 |
| GdRh | cP2 ClCs_2 | Gd | Rh | -72 | 2.1 | 0.5 | 3 | 8 | 0 | 7.00 | 4.00 | -0.589 | 0.004 | -1.441 | 0.007 | -1.108 | 0.012 | -0.094 | 0.003 |
| GdRh2 | cF24 Cu2Mg | Gd | Rh | -65 | 1.6 | 0.667 | 16 | 96 | 48 | 8.00 | 6.00 | -0.515 | 0.004 | -0.960 | 0.007 | -0.794 | 0.014 | -0.056 | 0.001 |
| GdRu2 | hP12 MgZn2 | Gd | Ru | -22 | 1.2 | 0.667 | 8 | 48 | 24 | 8.00 | 6.00 | -0.515 | 0.004 | -1.124 | 0.011 | -0.838 | 0.020 | -0.019 | 0.001 |
| GdSb | cF8 ClNa_1 | Gd | Sb | -103 | 6.1 | 0.5 | 0 | 24 | 0 | 3.00 | 3.00 | -1.373 | 0.004 | -0.913 | 0.009 | -1.321 | 0.018 | -0.178 | 0.011 |
| GdSn3 | cP4 AuCu3_2 | Sn | Gd | -50 | 3.0 | 0.25 | 12 | 12 | 0 | 6.00 | 6.00 | -0.520 | 0.003 | -0.687 | 0.004 | -0.647 | 0.011 | -0.043 | 0.003 |
| GdTl3 | cP4 AuCu3_2 | Tl | Gd | -37 | 2.2 | 0.25 | 12 | 12 | 0 | 6.00 | 6.00 | -0.315 | 0.001 | -0.687 | 0.004 | -0.532 | 0.005 | -0.032 | 0.002 |
| Ge3Hf5 | hP16 Mn5Si3 | Hf | Ge | -339 | 20.1 | 0.375 | 46 | 54 | 6 | 7.30 | 5.50 | -0.878 | 0.011 | -0.701 | 0.016 | -0.920 | 0.034 | -0.130 | 0.008 |
| Ge2Hf3 | tP10 Si2U3 | Hf | Ge | -152 | 9.0 | 0.4 | 32 | 32 | 2 | 8.00 | 4.50 | -0.801 | 0.011 | -0.857 | 0.016 | -0.927 | 0.034 | -0.098 | 0.006 |
| Ge3La5 | hP16 Mn5Si3 | La | Ge | -68 | 4.0 | 0.375 | 46 | 54 | 6 | 7.30 | 5.50 | -0.612 | 0.004 | -0.701 | 0.016 | -0.683 | 0.014 | -0.026 | 0.002 |
| Ge3Mn5 | hP16 Mn5Si3 | Mn | Ge | -12 | 0.7 | 0.375 | 46 | 54 | 6 | 7.30 | 5.50 | -0.402 | 0.007 | -0.701 | 0.016 | -0.556 | 0.019 | -0.005 | 0.000 |
| GeMo3 | cP8 Cr3Si | Mo | Ge | -15 | 0.9 | 0.25 | 30 | 24 | 0 | 7.00 | 6.00 | -0.976 | 0.006 | -0.643 | 0.016 | -0.822 | 0.020 | -0.013 | 0.001 |
| Ge3Mo5 | hP16 Mn5Si3 | Mo | Ge | -19 | 1.1 | 0.375 | 46 | 54 | 6 | 7.30 | 5.50 | -0.936 | 0.006 | -0.701 | 0.016 | -0.826 | 0.018 | -0.007 | 0.000 |
| Ge2Mo | oP12 Co2Si | Mo | Ge | -14 | 0.8 | 0.667 | 0 | 40 | 32 | 5.00 | 6.50 | -1.366 | 0.006 | -0.593 | 0.016 | -0.994 | 0.023 | -0.015 | 0.001 |
| GeNb3 | cP8 Cr3Si | Nb | Ge | -15 | 0.9 | 0.25 | 30 | 24 | 0 | 7.00 | 6.00 | -1.085 | 0.012 | -0.643 | 0.016 | -0.877 | 0.039 | -0.013 | 0.001 |
| Ge2Nb | hP9 CrSi2 | Nb | Ge | -29 | 1.7 | 0.667 | 6 | 30 | 27 | 7.00 | 7.00 | -1.085 | 0.012 | -0.551 | 0.016 | -0.848 | 0.033 | -0.030 | 0.002 |
| Ge3Pr5 | hP16 Mn5Si3 | Pr | Ge | -70 | 4.2 | 0.375 | 46 | 54 | 6 | 7.30 | 5.50 | -0.507 | 0.004 | -0.701 | 0.016 | -0.631 | 0.014 | -0.027 | 0.002 |
| Ge3Sc5 | hP16 Mn5Si3 | Sc | Ge | -93 | 5.5 | 0.375 | 46 | 54 | 6 | 7.30 | 5.50 | -0.536 | 0.007 | -0.701 | 0.016 | -0.655 | 0.021 | -0.036 | 0.002 |
| Ge3Y5 | hP16 Mn5Si3 | Y | Ge | -90 | 5.3 | 0.375 | 46 | 54 | 6 | 7.30 | 5.50 | -0.603 | 0.004 | -0.701 | 0.016 | -0.686 | 0.014 | -0.034 | 0.002 |
| HfIr | oC8 BCr | Hf | Ir | -97 | 3.2 | 0.5 | 20 | 28 | 4 | 8.50 | 4.50 | -0.754 | 0.011 | -1.541 | 0.007 | -1.291 | 0.029 | -0.144 | 0.005 |
| HfMo2 | cF24 Cu2Mg | Mo | Hf | -11 | 0.6 | 0.333 | 48 | 96 | 16 | 6.00 | 8.00 | -1.138 | 0.006 | -0.801 | 0.011 | -0.979 | 0.017 | -0.009 | 0.001 |
| HfN | cF8 ClNa_2 | Hf | N | -186 | 11.0 | 0.5 | 24 | 24 | 0 | 9.00 | 3.00 | -0.712 | 0.011 | -1.633 | 0.004 | -1.494 | 0.041 | -0.321 | 0.019 |
| HfNi5 | cF24 AuBe5 | Ni | Hf | -37 | 2.2 | 0.167 | 96 | 64 | 0 | 6.40 | 8.00 | -0.697 | 0.015 | -0.801 | 0.011 | -0.773 | 0.054 | -0.024 | 0.001 |
| Hf2Ni7 | mC36 Ni7Zr2 | Ni | Hf | -42 | 2.5 | 0.222 | 112 | 112 | 8 | 6.00 | 8.00 | -0.743 | 0.015 | -0.801 | 0.011 | -0.787 | 0.043 | -0.015 | 0.001 |
| HfNi3 | hR12 BaPb3 | Ni | Hf | -45 | 2.6 | 0.25 | 36 | 36 | 0 | 6.00 | 6.00 | -0.743 | 0.015 | -1.068 | 0.011 | -0.944 | 0.044 | -0.039 | 0.002 |
| Hf9Ni11 | tI40 Pt11Zr9 | Ni | Hf | -50 | 3.0 | 0.45 | 62 | 154 | 48 | 6.32 | 6.94 | -0.706 | 0.077 | -0.923 | 0.011 | -0.821 | 0.130 | -0.007 | 0.000 |
| HfNi | oC8 BCr | Ni | Hf | -51 | 3.0 | 0.5 | 4 | 28 | 20 | 4.50 | 8.50 | -0.991 | 0.015 | -0.754 | 0.011 | -0.948 | 0.036 | -0.076 | 0.004 |
| Hf2Ni | tI12 Al2Cu | Ni | Hf | -36 | 2.2 | 0.667 | 4 | 32 | 44 | 5.00 | 7.50 | -0.892 | 0.015 | -0.855 | 0.011 | -0.920 | 0.047 | -0.047 | 0.003 |
| HfPd3 | hP16 Ni3Ti | Pd | Hf | -92 | 1.5 | 0.25 | 48 | 48 | 0 | 6.00 | 6.00 | -0.651 | 0.004 | -1.068 | 0.011 | -0.939 | 0.016 | -0.080 | 0.001 |
| HfRh | cP2 ClCs_2 | Rh | Hf | -92 | 5.3 | 0.5 | 0 | 8 | 3 | 4.00 | 7.00 | -1.441 | 0.007 | -0.916 | 0.011 | -1.298 | 0.024 | -0.120 | 0.007 |
| HfRu | cP2 ClCs_2 | Hf | Ru | -92 | 5.2 | 0.5 | 3 | 8 | 0 | 7.00 | 4.00 | -0.916 | 0.011 | -1.686 | 0.011 | -1.420 | 0.027 | -0.119 | 0.007 |
| Hf2Si | tI12 Al2Cu | Hf | Si | -64 | 3.8 | 0.333 | 44 | 32 | 4 | 7.50 | 5.00 | -0.855 | 0.011 | -0.933 | 0.041 | -0.976 | 0.052 | -0.083 | 0.005 |
| Hf3Si2 | tP10 Si2U3 | Hf | Si | -77 | 4.5 | 0.4 | 32 | 32 | 2 | 8.00 | 4.50 | -0.801 | 0.011 | -1.036 | 0.041 | -0.969 | 0.039 | -0.050 | 0.003 |
| Hf5Si4 | tP36 Si4Zr5 | Hf | Si | -75 | 4.4 | 0.444 | 100 | 128 | 8 | 8.20 | 4.50 | -0.782 | 0.011 | -1.036 | 0.041 | -0.933 | 0.033 | -0.024 | 0.001 |
| HfSi | oP8 BFe | Hf | Si | -73 | 4.3 | 0.5 | 20 | 28 | 4 | 8.50 | 4.50 | -0.754 | 0.011 | -1.036 | 0.041 | -1.003 | 0.041 | -0.108 | 0.006 |
| HfSi2 | oC12 Si2Zr | Hf | Si | -70 | 4.1 | 0.667 | 12 | 40 | 12 | 8.00 | 4.00 | -0.801 | 0.011 | -1.166 | 0.041 | -1.056 | 0.043 | -0.072 | 0.004 |
| HfW2 | cF24 Cu2Mg | Hf | W | -14 | 0.8 | 0.667 | 16 | 96 | 48 | 8.00 | 6.00 | -0.801 | 0.011 | -1.470 | 0.009 | -1.148 | 0.024 | -0.012 | 0.001 |
| Hg2K | oI12 Hg2K | Hg | K | -26 | 1.5 | 0.333 | 24 | 48 | 8 | 6.00 | 8.00 | -0.106 | 0.000 | -0.115 | 0.001 | -0.133 | 0.002 | -0.022 | 0.001 |
| HgK | aP8 HgK | Hg | K | -27 | 4.2 | 0.5 | 6 | 30 | 16 | 5.25 | 7.75 | -0.121 | 0.006 | -0.119 | 0.001 | -0.157 | 0.013 | -0.037 | 0.006 |
| Hg3Li | hP8 Ni3Sn | Hg | Li | -26 | 1.6 | 0.25 | 24 | 24 | 0 | 6.00 | 6.00 | -0.106 | 0.000 | -0.275 | 0.001 | -0.213 | 0.002 | -0.023 | 0.001 |
| Hg4Ni | cI10 Hg4Pt | Hg | Ni | -9 | 0.5 | 0.2 | 24 | 16 | 0 | 4.00 | 4.00 | -0.159 | 0.000 | -1.114 | 0.015 | -0.649 | 0.012 | -0.012 | 0.001 |
| Hg5Pd2 | tP14 Hg5Mn2 | Hg | Pd | -32 | 1.9 | 0.286 | 30 | 40 | 4 | 5.00 | 6.00 | -0.127 | 0.000 | -0.651 | 0.004 | -0.405 | 0.004 | -0.017 | 0.001 |
| HgPd | tP4 AuCu | Hg | Pd | -46 | 2.7 | 0.5 | 4 | 16 | 4 | 6.00 | 6.00 | -0.106 | 0.000 | -0.651 | 0.004 | -0.438 | 0.007 | -0.060 | 0.004 |
| Hg4Pt | cI10 Hg4Pt | Hg | Pt | -15 | 0.9 | 0.2 | 24 | 16 | 0 | 4.00 | 4.00 | -0.159 | 0.000 | -1.466 | 0.002 | -0.832 | 0.003 | -0.019 | 0.001 |
| Hg2Pt | tP3 Hg2Pt | Hg | Pt | -13 | 0.8 | 0.333 | 6 | 8 | 1 | 5.00 | 5.00 | -0.127 | 0.000 | -1.173 | 0.002 | -0.667 | 0.003 | -0.017 | 0.001 |
| Hg2Rh | tP3 Hg2Pt | Hg | Rh | -18 | 1.1 | 0.333 | 6 | 8 | 1 | 5.00 | 5.00 | -0.127 | 0.000 | -1.153 | 0.007 | -0.663 | 0.008 | -0.023 | 0.001 |
| HgTe | cF8 SZn | Hg | Te | -22 | 1.3 | 0.5 | 0 | 16 | 0 | 2.00 | 2.00 | -0.318 | 0.000 | -1.019 | 0.015 | -0.725 | 0.009 | -0.056 | 0.003 |
| HoIn3 | cP4 AuCu3_2 | In | Ho | -40 | 2.4 | 0.25 | 12 | 12 | 0 | 6.00 | 6.00 | -0.420 | 0.007 | -0.519 | 0.004 | -0.504 | 0.021 | -0.035 | 0.002 |
| HoIr | cP2 ClCs_2 | Ho | Ir | -81 | 2.3 | 0.5 | 3 | 8 | 0 | 7.00 | 4.00 | -0.445 | 0.004 | -1.733 | 0.007 | -1.194 | 0.012 | -0.105 | 0.003 |
| HoIr2 | cF24 Cu2Mg | Ho | Ir | -74 | 2.0 | 0.667 | 16 | 96 | 48 | 8.00 | 6.00 | -0.389 | 0.004 | -1.156 | 0.007 | -0.837 | 0.015 | -0.064 | 0.002 |
| HoMg2 | hP12 MgZn2 | Mg | Ho | -14 | 0.8 | 0.333 | 24 | 48 | 8 | 6.00 | 8.00 | -0.254 | 0.001 | -0.389 | 0.004 | -0.334 | 0.005 | -0.012 | 0.001 |
| HoMg | cP2 ClCs_1 | Mg | Ho | -14 | 0.8 | 0.5 | 3 | 8 | 3 | 7.00 | 7.00 | -0.218 | 0.001 | -0.445 | 0.004 | -0.349 | 0.007 | -0.018 | 0.001 |
| HoPd | oP8 BFe | Ho | Pd | -76 | 2.2 | 0.5 | 20 | 28 | 4 | 8.50 | 4.50 | -0.367 | 0.004 | -0.867 | 0.004 | -0.729 | 0.013 | -0.112 | 0.003 |
| Ho3Pd4 | hR14 Pd4Pu3 | Ho | Pd | -94 | 2.1 | 0.571 | 21 | 54 | 20 | 8.00 | 5.88 | -0.389 | 0.004 | -0.664 | 0.025 | -0.563 | 0.054 | -0.036 | 0.001 |
| HoPd3 | cP4 AuCu3_2 | Ho | Pd | -87 | 2.3 | 0.75 | 0 | 12 | 12 | 6.00 | 6.00 | -0.519 | 0.004 | -0.651 | 0.004 | -0.660 | 0.013 | -0.075 | 0.002 |
| HoPt | oP8 BFe | Ho | Pt | -103 | 5.1 | 0.5 | 20 | 28 | 4 | 8.50 | 4.50 | -0.367 | 0.004 | -1.303 | 0.002 | -0.987 | 0.015 | -0.152 | 0.008 |
| HoPt2 | cF24 Cu2Mg | Ho | Pt | -107 | 4.8 | 0.667 | 16 | 96 | 48 | 8.00 | 6.00 | -0.389 | 0.004 | -0.977 | 0.002 | -0.775 | 0.010 | -0.092 | 0.004 |
| HoPt3 | cP4 AuCu3_2 | Ho | Pt | -95 | 2.3 | 0.75 | 0 | 12 | 12 | 6.00 | 6.00 | -0.519 | 0.004 | -0.977 | 0.002 | -0.831 | 0.009 | -0.082 | 0.002 |
| HoRh | cP2 ClCs_2 | Ho | Rh | -87 | 2.3 | 0.5 | 3 | 8 | 0 | 7.00 | 4.00 | -0.445 | 0.004 | -1.441 | 0.007 | -1.056 | 0.012 | -0.113 | 0.003 |
| HoRh2 | cF24 Cu2Mg | Ho | Rh | -70 | 1.5 | 0.667 | 16 | 96 | 48 | 8.00 | 6.00 | -0.389 | 0.004 | -0.960 | 0.007 | -0.736 | 0.014 | -0.061 | 0.001 |
| HoRu2 | hP12 MgZn2 | Ho | Ru | -27 | 1.3 | 0.667 | 8 | 48 | 24 | 8.00 | 6.00 | -0.389 | 0.004 | -1.124 | 0.011 | -0.780 | 0.020 | -0.023 | 0.001 |
| HoSb | cF8 ClNa_1 | Ho | Sb | -64 | 3.8 | 0.5 | 0 | 24 | 0 | 3.00 | 3.00 | -1.038 | 0.004 | -0.913 | 0.009 | -1.087 | 0.014 | -0.111 | 0.007 |
| In3La | cP4 AuCu3_2 | In | La | -57 | 3.4 | 0.25 | 12 | 12 | 0 | 6.00 | 6.00 | -0.420 | 0.007 | -0.745 | 0.004 | -0.631 | 0.022 | -0.049 | 0.003 |
| In2La | oI12 CeCu2 | In | La | -57 | 3.4 | 0.333 | 16 | 48 | 8 | 5.00 | 8.00 | -0.504 | 0.007 | -0.558 | 0.004 | -0.580 | 0.015 | -0.049 | 0.003 |
| In5La3 | oC32 Pd5Pu3 | In | La | -57 | 3.4 | 0.375 | 37 | 116 | 44 | 4.75 | 8.50 | -0.530 | 0.051 | -0.526 | 0.004 | -0.548 | 0.069 | -0.020 | 0.001 |
| InLa | cP2 ClCs_2 | In | La | -58 | 3.4 | 0.5 | 0 | 8 | 3 | 4.00 | 7.00 | -0.630 | 0.007 | -0.638 | 0.004 | -0.709 | 0.014 | -0.075 | 0.004 |
| InLa2 | hP6 InNi2 | In | La | -38 | 2.2 | 0.667 | 0 | 22 | 14 | 5.50 | 6.25 | -0.458 | 0.007 | -0.715 | 0.004 | -0.622 | 0.012 | -0.036 | 0.002 |
| InLa3 | cP4 AuCu3_2 | In | La | -28 | 1.7 | 0.75 | 0 | 12 | 12 | 6.00 | 6.00 | -0.420 | 0.007 | -0.745 | 0.004 | -0.606 | 0.014 | -0.024 | 0.001 |
| In3Lu | cP4 AuCu3_2 | In | Lu | -35 | 2.1 | 0.25 | 12 | 12 | 0 | 6.00 | 6.00 | -0.420 | 0.007 | -0.739 | 0.004 | -0.609 | 0.021 | -0.030 | 0.002 |
| In3Nd | cP4 AuCu3_2 | In | Nd | -58 | 3.4 | 0.25 | 12 | 12 | 0 | 6.00 | 6.00 | -0.420 | 0.007 | -0.565 | 0.004 | -0.542 | 0.022 | -0.050 | 0.003 |
| InNd | cP2 ClCs_2 | In | Nd | -57 | 3.4 | 0.5 | 0 | 8 | 3 | 4.00 | 7.00 | -0.630 | 0.007 | -0.484 | 0.004 | -0.631 | 0.014 | -0.074 | 0.004 |
| InP | cF8 SZn | In | P | -37 | 2.2 | 0.5 | 0 | 16 | 0 | 2.00 | 2.00 | -1.259 | 0.007 | -1.640 | 0.007 | -1.546 | 0.019 | -0.097 | 0.006 |
| In3Pd2 | hP5 Al3Ni2 | In | Pd | -56 | 3.3 | 0.4 | 9 | 16 | 3 | 5.67 | 5.50 | -0.444 | 0.007 | -0.710 | 0.004 | -0.614 | 0.017 | -0.037 | 0.002 |
| In3Pd5 | oP16 Ge3Rh5 | In | Pd | -61 | 3.6 | 0.625 | 5 | 57 | 38 | 5.58 | 6.65 | -0.451 | 0.076 | -0.587 | 0.004 | -0.541 | 0.068 | -0.022 | 0.002 |
| InPd2 | oP12 Co2Si | In | Pd | -59 | 3.5 | 0.667 | 0 | 40 | 32 | 5.00 | 6.50 | -0.504 | 0.007 | -0.600 | 0.004 | -0.613 | 0.015 | -0.061 | 0.004 |
| In3Pr | cP4 AuCu3_2 | In | Pr | -60 | 3.6 | 0.25 | 12 | 12 | 0 | 6.00 | 6.00 | -0.420 | 0.007 | -0.617 | 0.004 | -0.570 | 0.022 | -0.052 | 0.003 |
| InPr2 | hP6 InNi2 | In | Pr | -33 | 2.0 | 0.667 | 0 | 22 | 14 | 5.50 | 6.25 | -0.458 | 0.007 | -0.592 | 0.004 | -0.556 | 0.012 | -0.031 | 0.002 |
| InPr3 | cP4 AuCu3_2 | In | Pr | -25 | 1.5 | 0.75 | 0 | 12 | 12 | 6.00 | 6.00 | -0.420 | 0.007 | -0.617 | 0.004 | -0.540 | 0.014 | -0.022 | 0.001 |
| InSb | cF8 SZn | In | Sb | -16 | 1.0 | 0.5 | 0 | 16 | 0 | 2.00 | 2.00 | -1.259 | 0.007 | -1.370 | 0.009 | -1.357 | 0.019 | -0.042 | 0.002 |
| In3Sm | cP4 AuCu3_2 | Sm | In | -50 | 3.0 | 0.75 | 0 | 12 | 12 | 6.00 | 6.00 | -0.357 | 0.004 | -0.420 | 0.007 | -0.431 | 0.022 | -0.043 | 0.003 |
| In3Tb | cP4 AuCu3_2 | In | Tb | -45 | 2.7 | 0.25 | 12 | 12 | 0 | 6.00 | 6.00 | -0.420 | 0.007 | -0.671 | 0.004 | -0.584 | 0.021 | -0.039 | 0.002 |
| In3Tm | cP4 AuCu3_2 | Tm | In | -36 | 2.2 | 0.75 | 0 | 12 | 12 | 6.00 | 6.00 | -0.401 | 0.004 | -0.420 | 0.007 | -0.442 | 0.021 | -0.031 | 0.002 |
| In3Y | cP4 AuCu3_2 | In | Y | -42 | 2.5 | 0.25 | 12 | 12 | 0 | 6.00 | 6.00 | -0.420 | 0.007 | -0.734 | 0.004 | -0.613 | 0.021 | -0.036 | 0.002 |
| In3Yb | cP4 AuCu3_2 | Yb | In | -40 | 2.4 | 0.75 | 0 | 12 | 12 | 6.00 | 6.00 | -0.269 | 0.004 | -0.420 | 0.007 | -0.379 | 0.021 | -0.034 | 0.002 |
| Ir2La | cF24 Cu2Mg | La | Ir | -63 | 2.2 | 0.667 | 16 | 96 | 48 | 8.00 | 6.00 | -0.558 | 0.004 | -1.156 | 0.007 | -0.911 | 0.015 | -0.054 | 0.002 |
| Ir3La | hR12 Be3Nb | La | Ir | -50 | 2.2 | 0.75 | 5 | 42 | 33 | 8.67 | 6.00 | -0.515 | 0.004 | -1.156 | 0.007 | -0.872 | 0.018 | -0.037 | 0.002 |
| IrLu | cP2 ClCs_2 | Lu | Ir | -86 | 4.8 | 0.5 | 3 | 8 | 0 | 7.00 | 4.00 | -0.633 | 0.004 | -1.733 | 0.007 | -1.294 | 0.015 | -0.111 | 0.006 |
| Ir2Lu | cF24 Cu2Mg | Lu | Ir | -71 | 1.2 | 0.667 | 16 | 96 | 48 | 8.00 | 6.00 | -0.554 | 0.004 | -1.156 | 0.007 | -0.916 | 0.014 | -0.061 | 0.001 |
| Ir2Nd | cF24 Cu2Mg | Nd | Ir | -68 | 1.5 | 0.667 | 16 | 96 | 48 | 8.00 | 6.00 | -0.424 | 0.004 | -1.156 | 0.007 | -0.848 | 0.014 | -0.058 | 0.001 |
| Ir2Pr | cF24 Cu2Mg | Pr | Ir | -71 | 2.8 | 0.667 | 16 | 96 | 48 | 8.00 | 6.00 | -0.462 | 0.004 | -1.156 | 0.007 | -0.870 | 0.015 | -0.061 | 0.002 |
| Ir2Tb | cF24 Cu2Mg | Tb | Ir | -71 | 2.6 | 0.667 | 16 | 96 | 48 | 8.00 | 6.00 | -0.504 | 0.004 | -1.156 | 0.007 | -0.891 | 0.015 | -0.061 | 0.002 |
| IrTi | tP2 HgMn | Ti | Ir | -84 | 3.5 | 0.5 | 3 | 8 | 3 | 7.00 | 7.00 | -0.700 | 0.005 | -0.991 | 0.007 | -0.954 | 0.018 | -0.109 | 0.005 |
| IrY | cP2 ClCs_2 | Y | Ir | -66 | 3.8 | 0.5 | 3 | 8 | 0 | 7.00 | 4.00 | -0.629 | 0.004 | -1.733 | 0.007 | -1.266 | 0.014 | -0.085 | 0.005 |
| IrZr3 | tI32 SV3 | Zr | Ir | -69 | 4.1 | 0.25 | 136 | 72 | 0 | 7.17 | 4.50 | -0.882 | 0.015 | -1.541 | 0.007 | -1.291 | 0.066 | -0.080 | 0.005 |
| IrZr2 | tI12 Al2Cu | Zr | Ir | -88 | 5.2 | 0.333 | 44 | 32 | 4 | 7.50 | 5.00 | -0.843 | 0.015 | -1.387 | 0.007 | -1.229 | 0.055 | -0.115 | 0.007 |
| IrZr | cP2 ClCs_1 | Zr | Ir | -90 | 5.3 | 0.5 | 3 | 8 | 3 | 7.00 | 7.00 | -0.903 | 0.015 | -0.991 | 0.007 | -1.063 | 0.031 | -0.116 | 0.007 |
| Ir2Zr | cF24 Cu2Mg | Zr | Ir | -86 | 5.1 | 0.667 | 16 | 96 | 48 | 8.00 | 6.00 | -0.790 | 0.015 | -1.156 | 0.007 | -1.047 | 0.024 | -0.074 | 0.004 |
| Ir3Zr | cP4 AuCu3_2 | Zr | Ir | -81 | 4.8 | 0.75 | 0 | 12 | 12 | 6.00 | 6.00 | -1.054 | 0.015 | -1.156 | 0.007 | -1.174 | 0.029 | -0.070 | 0.004 |
| K3Sb | hP8 AsNa3 | K | Sb | -75 | 8.4 | 0.25 | 20 | 22 | 0 | 5.17 | 5.50 | -0.179 | 0.001 | -0.498 | 0.009 | -0.409 | 0.014 | -0.070 | 0.008 |
| La2Mg17 | hP38 Ni17Th2 | Mg | La | -9 | 0.5 | 0.105 | 170 | 76 | 0 | 6.12 | 9.50 | -0.249 | 0.007 | -0.470 | 0.004 | -0.362 | 0.041 | -0.002 | 0.000 |
| LaMg3 | cF16 BiF3 | Mg | La | -20 | 1.2 | 0.25 | 56 | 56 | 0 | 7.00 | 7.00 | -0.218 | 0.001 | -0.638 | 0.004 | -0.443 | 0.006 | -0.015 | 0.001 |
| LaMg | cP2 ClCs_2 | Mg | La | -17 | 1.0 | 0.5 | 0 | 8 | 3 | 4.00 | 7.00 | -0.381 | 0.001 | -0.638 | 0.004 | -0.531 | 0.006 | -0.022 | 0.001 |
| LaNi5 | hP6 CaCu5 | Ni | La | -27 | 1.6 | 0.167 | 21 | 18 | 0 | 6.00 | 9.00 | -0.743 | 0.015 | -0.496 | 0.004 | -0.635 | 0.043 | -0.016 | 0.001 |
| La2Ni7 | hP36 Ce2Ni7 | Ni | La | -28 | 1.7 | 0.222 | 108 | 120 | 11 | 6.00 | 8.88 | -0.743 | 0.015 | -0.503 | 0.010 | -0.633 | 0.041 | -0.010 | 0.001 |
| LaNi3 | hR12 Be3Nb | Ni | La | -29 | 1.7 | 0.25 | 33 | 42 | 5 | 6.00 | 8.67 | -0.743 | 0.015 | -0.515 | 0.004 | -0.650 | 0.033 | -0.021 | 0.001 |
| La2Ni3 | oC20 La2Ni3 | Ni | La | -28 | 1.7 | 0.4 | 24 | 80 | 20 | 5.33 | 7.50 | -0.836 | 0.015 | -0.596 | 0.004 | -0.730 | 0.021 | -0.015 | 0.001 |
| LaNi | oC8 BCr | Ni | La | -27 | 1.6 | 0.5 | 4 | 28 | 20 | 4.50 | 8.50 | -0.991 | 0.015 | -0.526 | 0.004 | -0.798 | 0.023 | -0.040 | 0.002 |
| La7Ni3 | hP20 Fe3Th7 | Ni | La | -16 | 1.0 | 0.7 | 0 | 53 | 78 | 4.38 | 7.45 | -1.019 | 0.029 | -0.600 | 0.007 | -0.816 | 0.050 | -0.006 | 0.001 |
| La3Ni | oP16 CFe3 | Ni | La | -13 | 0.8 | 0.75 | 2 | 34 | 68 | 4.75 | 7.08 | -0.938 | 0.152 | -0.631 | 0.011 | -0.801 | 0.152 | -0.016 | 0.002 |
| LaPb3 | cP4 AuCu3_2 | Pb | La | -57 | 3.4 | 0.25 | 12 | 12 | 0 | 6.00 | 6.00 | -0.337 | 0.001 | -0.745 | 0.004 | -0.590 | 0.008 | -0.049 | 0.003 |
| La4Pb3 | cI28 P4Th3 | Pb | La | -70 | 4.1 | 0.571 | 48 | 96 | 40 | 8.00 | 5.50 | -0.253 | 0.001 | -0.812 | 0.004 | -0.563 | 0.009 | -0.030 | 0.002 |
| La5Pb3 | hP16 Mn5Si3 | Pb | La | -72 | 4.3 | 0.625 | 6 | 54 | 46 | 5.50 | 7.30 | -0.368 | 0.001 | -0.612 | 0.004 | -0.518 | 0.010 | -0.028 | 0.002 |
| LaPt | oC8 BCr | La | Pt | -93 | 2.9 | 0.5 | 20 | 28 | 4 | 8.50 | 4.50 | -0.526 | 0.004 | -1.303 | 0.002 | -1.052 | 0.012 | -0.138 | 0.004 |
| LaPt2 | cF24 Cu2Mg | La | Pt | -90 | 2.9 | 0.667 | 16 | 96 | 48 | 8.00 | 6.00 | -0.558 | 0.004 | -0.977 | 0.002 | -0.846 | 0.008 | -0.078 | 0.003 |
| LaRh | oC8 BCr | La | Rh | -56 | 2.3 | 0.5 | 20 | 28 | 4 | 8.50 | 4.50 | -0.526 | 0.004 | -1.281 | 0.007 | -0.986 | 0.016 | -0.083 | 0.003 |
| LaRh2 | cF24 Cu2Mg | La | Rh | -58 | 1.7 | 0.667 | 16 | 96 | 48 | 8.00 | 6.00 | -0.558 | 0.004 | -0.960 | 0.007 | -0.809 | 0.014 | -0.050 | 0.001 |
| LaRu2 | cF24 Cu2Mg | La | Ru | -11 | 1.9 | 0.667 | 16 | 96 | 48 | 8.00 | 6.00 | -0.558 | 0.004 | -1.124 | 0.011 | -0.850 | 0.020 | -0.009 | 0.002 |
| La2Sb | tI12 La2Sb | La | Sb | -98 | 5.8 | 0.333 | 40 | 36 | 0 | 7.25 | 4.50 | -0.616 | 0.004 | -0.609 | 0.009 | -0.725 | 0.018 | -0.113 | 0.007 |
| LaSb | cF8 ClNa_1 | La | Sb | -142 | 8.4 | 0.5 | 0 | 24 | 0 | 3.00 | 3.00 | -1.489 | 0.004 | -0.913 | 0.009 | -1.447 | 0.023 | -0.246 | 0.015 |
| LaSb2 | oC24 Sb2Sm | La | Sb | -92 | 5.5 | 0.667 | 0 | 72 | 20 | 4.50 | 3.50 | -0.993 | 0.004 | -0.783 | 0.009 | -0.994 | 0.017 | -0.106 | 0.006 |
| LaSn3 | cP4 AuCu3_2 | Sn | La | -61 | 3.6 | 0.25 | 12 | 12 | 0 | 6.00 | 6.00 | -0.520 | 0.003 | -0.745 | 0.004 | -0.685 | 0.012 | -0.053 | 0.003 |
| La3Sn5 | oC32 Pd5Pu3 | Sn | La | -78 | 4.6 | 0.375 | 44 | 116 | 37 | 5.10 | 7.92 | -0.612 | 0.003 | -0.564 | 0.059 | -0.616 | 0.070 | -0.028 | 0.002 |
| La5Sn4 | oP36 Ge4Sm5 | Sn | La | -87 | 5.2 | 0.556 | 6 | 126 | 88 | 4.31 | 7.55 | -0.724 | 0.032 | -0.592 | 0.005 | -0.687 | 0.034 | -0.029 | 0.002 |
| La5Sn3 | tI32 Si3W5 | Sn | La | -75 | 4.5 | 0.625 | 4 | 112 | 92 | 5.00 | 7.40 | -0.624 | 0.003 | -0.604 | 0.004 | -0.642 | 0.010 | -0.028 | 0.002 |
| LaTl3 | cP4 AuCu3_2 | Tl | La | -45 | 2.7 | 0.25 | 12 | 12 | 0 | 6.00 | 6.00 | -0.315 | 0.001 | -0.745 | 0.004 | -0.569 | 0.006 | -0.039 | 0.002 |
| Li22Sn5 | cF432 Li22Pb5 | Li | Sn | -43 | 2.6 | 0.185 | 1904 | 1120 | 0 | 7.00 | 7.00 | -0.236 | 0.001 | -0.446 | 0.003 | -0.347 | 0.007 | -0.006 | 0.000 |
| Li7Sn2 | oC36 Ge2Li7 | Li | Sn | -42 | 2.5 | 0.222 | 142 | 104 | 2 | 6.93 | 6.75 | -0.238 | 0.002 | -0.462 | 0.009 | -0.367 | 0.023 | -0.017 | 0.002 |
| Li13Sn5 | hP18 Li13Sn5 | Li | Sn | -40 | 2.4 | 0.278 | 58 | 66 | 2 | 7.00 | 7.00 | -0.236 | 0.001 | -0.446 | 0.003 | -0.347 | 0.005 | -0.006 | 0.000 |
| Li7Sn3 | mP20 Li7Sn3 | Li | Sn | -39 | 2.3 | 0.3 | 56 | 74 | 5 | 6.64 | 7.00 | -0.249 | 0.013 | -0.446 | 0.006 | -0.358 | 0.038 | -0.011 | 0.001 |
| LiSn | mP6 LiSn | Li | Sn | -36 | 2.1 | 0.5 | 6 | 24 | 6 | 6.00 | 6.00 | -0.275 | 0.001 | -0.520 | 0.003 | -0.444 | 0.007 | -0.047 | 0.003 |
| Li2Sn5 | tP14 Hg5Mn2 | Li | Sn | -22 | 1.3 | 0.714 | 4 | 40 | 30 | 6.00 | 5.00 | -0.275 | 0.001 | -0.624 | 0.003 | -0.461 | 0.008 | -0.011 | 0.001 |
| LiTl | cP2 ClCs_1 | Li | Tl | -27 | 1.6 | 0.5 | 3 | 8 | 3 | 7.00 | 7.00 | -0.236 | 0.001 | -0.270 | 0.001 | -0.288 | 0.005 | -0.035 | 0.002 |
| LuPb2 | tI6 MoSi2 | Pb | Lu | -33 | 2.0 | 0.333 | 18 | 20 | 4 | 7.00 | 7.00 | -0.289 | 0.001 | -0.633 | 0.004 | -0.495 | 0.008 | -0.034 | 0.002 |
| LuPt | oP8 BFe | Lu | Pt | -118 | 2.2 | 0.5 | 20 | 28 | 4 | 8.50 | 4.50 | -0.521 | 0.004 | -1.303 | 0.002 | -1.087 | 0.011 | -0.175 | 0.003 |
| LuPt3 | cP4 AuCu3_2 | Lu | Pt | -103 | 2.7 | 0.75 | 0 | 12 | 12 | 6.00 | 6.00 | -0.739 | 0.004 | -0.977 | 0.002 | -0.947 | 0.010 | -0.089 | 0.002 |
| LuRh2 | cF24 Cu2Mg | Lu | Rh | -68 | 3.2 | 0.667 | 16 | 96 | 48 | 8.00 | 6.00 | -0.554 | 0.004 | -0.960 | 0.007 | -0.816 | 0.016 | -0.059 | 0.003 |
| Mg2Ni | hP18 Mg2Ni | Mg | Ni | -18 | 1.1 | 0.333 | 66 | 48 | 6 | 7.50 | 5.00 | -0.203 | 0.001 | -0.892 | 0.015 | -0.571 | 0.018 | -0.023 | 0.001 |
| MgNi2 | hP24 MgNi2 | Mg | Ni | -18 | 1.1 | 0.667 | 16 | 96 | 48 | 8.00 | 6.00 | -0.191 | 0.001 | -0.743 | 0.015 | -0.483 | 0.024 | -0.016 | 0.001 |
| MgO | cF8 ClNa_2 | Mg | O | -301 | 17.8 | 0.5 | 24 | 24 | 0 | 9.00 | 3.00 | -0.169 | 0.001 | -0.861 | 0.000 | -1.035 | 0.034 | -0.520 | 0.031 |
| Mg2Pb | cF12 CaF2 | Mg | Pb | -18 | 1.3 | 0.333 | 24 | 32 | 24 | 5.00 | 10.00 | -0.305 | 0.001 | -0.202 | 0.001 | -0.276 | 0.007 | -0.023 | 0.002 |
| Mg3Pr | cF16 BiF3 | Mg | Pr | -11 | 2.1 | 0.25 | 56 | 56 | 0 | 7.00 | 7.00 | -0.218 | 0.001 | -0.528 | 0.004 | -0.382 | 0.006 | -0.008 | 0.002 |
| MgPr | cP2 ClCs_1 | Mg | Pr | -17 | 2.1 | 0.5 | 3 | 8 | 3 | 7.00 | 7.00 | -0.218 | 0.001 | -0.528 | 0.004 | -0.395 | 0.008 | -0.022 | 0.003 |
| MgSc | cP2 ClCs_1 | Mg | Sc | -23 | 1.3 | 0.5 | 3 | 8 | 3 | 7.00 | 7.00 | -0.218 | 0.001 | -0.559 | 0.007 | -0.418 | 0.011 | -0.029 | 0.002 |
| Mg2Si | cF12 CaF2 | Mg | Si | -21 | 1.3 | 0.333 | 24 | 32 | 24 | 5.00 | 10.00 | -0.305 | 0.001 | -0.466 | 0.041 | -0.413 | 0.046 | -0.028 | 0.002 |
| Mg2Tm | hP12 MgZn2 | Mg | Tm | -9 | 0.6 | 0.333 | 24 | 48 | 8 | 6.00 | 8.00 | -0.254 | 0.001 | -0.301 | 0.004 | -0.286 | 0.005 | -0.008 | 0.000 |
| MgTm | cP2 ClCs_1 | Mg | Tm | -10 | 0.6 | 0.5 | 3 | 8 | 3 | 7.00 | 7.00 | -0.218 | 0.001 | -0.344 | 0.004 | -0.293 | 0.006 | -0.013 | 0.001 |
| Mg2Y | hP12 MgZn2 | Mg | Y | -14 | 0.8 | 0.333 | 24 | 48 | 8 | 6.00 | 8.00 | -0.254 | 0.001 | -0.550 | 0.004 | -0.414 | 0.005 | -0.012 | 0.001 |
| Mg2Yb | hP12 MgZn2 | Mg | Yb | -13 | 0.7 | 0.333 | 24 | 48 | 8 | 6.00 | 8.00 | -0.254 | 0.001 | -0.202 | 0.004 | -0.239 | 0.005 | -0.011 | 0.001 |
| Mg2Zn11 | cP39 Mg2Zn11 | Zn | Mg | -6 | 0.3 | 0.154 | 153 | 93 | 3 | 6.05 | 8.25 | -0.224 | 0.002 | -0.185 | 0.007 | -0.206 | 0.012 | -0.002 | 0.000 |
| MgZn2 | hP12 MgZn2 | Zn | Mg | -12 | 0.7 | 0.333 | 24 | 48 | 8 | 6.00 | 8.00 | -0.225 | 0.001 | -0.191 | 0.001 | -0.218 | 0.003 | -0.010 | 0.001 |
| MnO | cF8 ClNa_2 | Mn | O | -193 | 11.5 | 0.5 | 24 | 24 | 0 | 9.00 | 3.00 | -0.326 | 0.007 | -0.861 | 0.000 | -0.928 | 0.034 | -0.334 | 0.020 |
| Mn3P | tI32 Ni3P | Mn | P | -45 | 2.6 | 0.25 | 128 | 72 | 0 | 6.83 | 4.50 | -0.430 | 0.007 | -0.729 | 0.007 | -0.631 | 0.031 | -0.051 | 0.003 |
| Mn2P | hP9 Fe2P | Mn | P | -50 | 3.0 | 0.333 | 29 | 28 | 0 | 7.06 | 4.63 | -0.416 | 0.018 | -0.709 | 0.011 | -0.619 | 0.072 | -0.056 | 0.005 |
| MnP | oP8 MnP | Mn | P | -50 | 3.0 | 0.5 | 12 | 24 | 4 | 6.00 | 4.00 | -0.489 | 0.007 | -0.820 | 0.007 | -0.741 | 0.019 | -0.086 | 0.005 |
| Mn2Sb | tP6 Cu2Sb | Mn | Sb | -11 | 0.7 | 0.333 | 12 | 18 | 0 | 5.25 | 4.50 | -0.559 | 0.007 | -0.609 | 0.009 | -0.597 | 0.018 | -0.013 | 0.001 |
| MnSb | hP4 AsNi | Mn | Sb | -20 | 1.2 | 0.5 | 2 | 12 | 0 | 4.00 | 3.00 | -0.734 | 0.007 | -0.913 | 0.009 | -0.857 | 0.015 | -0.034 | 0.002 |
| Mn3Si | cF16 BiF3 | Mn | Si | -18 | 1.1 | 0.25 | 56 | 56 | 0 | 7.00 | 7.00 | -0.419 | 0.007 | -0.666 | 0.041 | -0.556 | 0.023 | -0.014 | 0.001 |
| Mn5Si3 | hP16 Mn5Si3 | Mn | Si | -25 | 1.5 | 0.375 | 46 | 54 | 6 | 7.30 | 5.50 | -0.402 | 0.007 | -0.848 | 0.041 | -0.635 | 0.028 | -0.010 | 0.001 |
| MnSi | cP8 FeSi | Mn | Si | -28 | 1.7 | 0.5 | 12 | 28 | 12 | 6.50 | 6.50 | -0.452 | 0.007 | -0.718 | 0.041 | -0.626 | 0.041 | -0.041 | 0.002 |
| Mn2Ti | hP12 MgZn2 | Mn | Ti | -9 | 0.5 | 0.333 | 24 | 48 | 8 | 6.00 | 8.00 | -0.489 | 0.007 | -0.613 | 0.005 | -0.559 | 0.014 | -0.008 | 0.000 |
| Mn12Y | tI26 Mn12Th | Mn | Y | -2 | 0.1 | 0.077 | 132 | 40 | 0 | 6.33 | 10.00 | -0.464 | 0.007 | -0.440 | 0.004 | -0.453 | 0.049 | -0.001 | 0.000 |
| Mn23Y6 | cF116 Mn23Th6 | Mn | Y | -5 | 0.3 | 0.207 | 416 | 306 | 48 | 6.18 | 8.38 | -0.475 | 0.007 | -0.526 | 0.007 | -0.501 | 0.035 | -0.001 | 0.000 |
| Mn2Y | cF24 Cu2Mg | Mn | Y | -3 | 0.2 | 0.333 | 48 | 96 | 16 | 6.00 | 8.00 | -0.489 | 0.007 | -0.550 | 0.004 | -0.522 | 0.013 | -0.002 | 0.000 |
| Mn2Zr | hP12 MgZn2 | Mn | Zr | -40 | 2.4 | 0.333 | 24 | 48 | 8 | 6.00 | 8.00 | -0.489 | 0.007 | -0.790 | 0.015 | -0.674 | 0.022 | -0.035 | 0.002 |
| MoNi4 | tI10 MoNi4 | Ni | Mo | -2 | 0.1 | 0.2 | 36 | 24 | 0 | 6.00 | 6.00 | -0.743 | 0.015 | -1.138 | 0.006 | -0.942 | 0.054 | -0.002 | 0.000 |
| MoNi3 | oP8 Cu3Ti | Ni | Mo | -2 | 0.1 | 0.25 | 24 | 24 | 0 | 6.00 | 6.00 | -0.743 | 0.015 | -1.138 | 0.006 | -0.942 | 0.040 | -0.002 | 0.000 |
| MoNi | oP56 MoNi | Ni | Mo | -1 | 0.1 | 0.5 | 102 | 204 | 70 | 7.29 | 6.14 | -0.612 | 0.015 | -1.112 | 0.006 | -0.863 | 0.027 | -0.002 | 0.000 |
| Mo3Si | cP8 Cr3Si | Mo | Si | -31 | 1.8 | 0.25 | 30 | 24 | 0 | 7.00 | 6.00 | -0.976 | 0.006 | -0.777 | 0.041 | -0.903 | 0.025 | -0.027 | 0.002 |
| Mo5Si3 | tI32 Si3W5 | Mo | Si | -39 | 2.3 | 0.375 | 92 | 112 | 4 | 7.40 | 5.00 | -0.923 | 0.006 | -0.933 | 0.041 | -0.942 | 0.023 | -0.015 | 0.001 |
| Mo3Sn | cP8 Cr3Si | Sn | Mo | -4 | 0.2 | 0.75 | 0 | 24 | 30 | 6.00 | 7.00 | -0.520 | 0.003 | -0.976 | 0.006 | -0.751 | 0.018 | -0.003 | 0.000 |
| MoU2 | tI6 MoSi2 | U | Mo | -1 | 0.1 | 0.333 | 18 | 20 | 4 | 7.00 | 7.00 | -0.789 | 0.014 | -0.976 | 0.006 | -0.884 | 0.034 | -0.001 | 0.000 |
| Mo2Zr | cF24 Cu2Mg | Mo | Zr | -7 | 0.4 | 0.333 | 48 | 96 | 16 | 6.00 | 8.00 | -1.138 | 0.006 | -0.790 | 0.015 | -0.971 | 0.019 | -0.006 | 0.000 |
| NNb | hP4 AsNi | Nb | N | -118 | 7.0 | 0.5 | 2 | 12 | 0 | 4.00 | 3.00 | -1.899 | 0.012 | -1.633 | 0.004 | -1.970 | 0.029 | -0.204 | 0.012 |
| NTa2 | hP9 NV2 | Ta | N | -91 | 5.4 | 0.333 | 0 | 18 | 0 | 1.49 | 2.98 | -5.440 | 0.004 | -1.644 | 0.004 | -3.699 | 0.019 | -0.158 | 0.009 |
| NTa | hP6 NTa | Ta | N | -120 | 7.1 | 0.5 | 0 | 15 | 0 | 2.50 | 2.50 | -3.242 | 0.004 | -1.960 | 0.004 | -2.850 | 0.021 | -0.249 | 0.015 |
| NTi2 | tI12 NTi2 | Ti | N | -133 | 7.9 | 0.333 | 0 | 24 | 0 | 1.50 | 3.00 | -3.268 | 0.005 | -1.633 | 0.004 | -2.681 | 0.025 | -0.230 | 0.014 |
| NY | cF8 ClNa_2 | Y | N | -150 | 8.9 | 0.5 | 24 | 24 | 0 | 9.00 | 3.00 | -0.489 | 0.004 | -1.633 | 0.004 | -1.320 | 0.023 | -0.259 | 0.015 |
| NZr | cF8 ClNa_2 | Zr | N | -182 | 10.8 | 0.5 | 24 | 24 | 0 | 9.00 | 3.00 | -0.702 | 0.015 | -1.633 | 0.004 | -1.482 | 0.048 | -0.314 | 0.019 |
| NaPb | tI64 NaPb | Na | Pb | -24 | 1.4 | 0.5 | 72 | 224 | 64 | 5.75 | 5.50 | -0.194 | 0.001 | -0.368 | 0.001 | -0.317 | 0.005 | -0.036 | 0.002 |
| Na3Sb | hP8 AsNa3 | Na | Sb | -49 | 6.3 | 0.25 | 20 | 22 | 0 | 5.17 | 5.50 | -0.216 | 0.001 | -0.498 | 0.009 | -0.403 | 0.011 | -0.047 | 0.006 |
| NaSb | mP16 AsLi | Na | Sb | -33 | 2.0 | 0.5 | 20 | 51 | 8 | 5.69 | 4.19 | -0.196 | 0.015 | -0.654 | 0.009 | -0.479 | 0.052 | -0.054 | 0.006 |
| NbNi3 | oP8 Cu3Ti | Ni | Nb | -34 | 2.0 | 0.25 | 24 | 24 | 0 | 6.00 | 6.00 | -0.743 | 0.015 | -1.266 | 0.012 | -1.034 | 0.045 | -0.029 | 0.002 |
| Nb7Ni6 | hR13 Fe7W6 | Ni | Nb | -22 | 1.3 | 0.538 | 15 | 48 | 18 | 6.50 | 6.00 | -0.686 | 0.108 | -1.266 | 0.012 | -0.981 | 0.147 | -0.005 | 0.000 |
| NbPt3 | oP8 Cu3Ti | Pt | Nb | -47 | 3.7 | 0.25 | 24 | 24 | 0 | 6.00 | 6.00 | -0.977 | 0.002 | -1.266 | 0.012 | -1.162 | 0.016 | -0.040 | 0.003 |
| NbSi2 | hP9 CrSi2 | Nb | Si | -46 | 2.7 | 0.667 | 6 | 30 | 27 | 7.00 | 7.00 | -1.085 | 0.012 | -0.666 | 0.041 | -0.923 | 0.067 | -0.048 | 0.003 |
| NdNi | oC8 BCr | Nd | Ni | -25 | 1.5 | 0.5 | 20 | 28 | 4 | 8.50 | 4.50 | -0.399 | 0.004 | -0.991 | 0.015 | -0.732 | 0.022 | -0.037 | 0.002 |
| NdNi5 | hP6 CaCu5 | Nd | Ni | -26 | 1.1 | 0.833 | 0 | 18 | 21 | 9.00 | 6.00 | -0.376 | 0.004 | -0.743 | 0.015 | -0.575 | 0.043 | -0.015 | 0.001 |
| NdPd | oC8 BCr | Nd | Pd | -72 | 2.7 | 0.5 | 20 | 28 | 4 | 8.50 | 4.50 | -0.399 | 0.004 | -0.867 | 0.004 | -0.740 | 0.013 | -0.107 | 0.004 |
| NdPd3 | cP4 AuCu3_2 | Nd | Pd | -73 | 2.3 | 0.75 | 0 | 12 | 12 | 6.00 | 6.00 | -0.565 | 0.004 | -0.651 | 0.004 | -0.671 | 0.013 | -0.063 | 0.002 |
| NdPt | oP8 BFe | Nd | Pt | -102 | 2.6 | 0.5 | 20 | 28 | 4 | 8.50 | 4.50 | -0.399 | 0.004 | -1.303 | 0.002 | -1.001 | 0.012 | -0.151 | 0.004 |
| NdPt2 | cF24 Cu2Mg | Nd | Pt | -98 | 2.4 | 0.667 | 16 | 96 | 48 | 8.00 | 6.00 | -0.424 | 0.004 | -0.977 | 0.002 | -0.785 | 0.008 | -0.085 | 0.002 |
| NdPt5 | hP6 CaCu5 | Nd | Pt | -55 | 2.1 | 0.833 | 0 | 18 | 21 | 9.00 | 6.00 | -0.376 | 0.004 | -0.977 | 0.002 | -0.708 | 0.009 | -0.032 | 0.001 |
| Nd5Rh4 | oP36 Ge4Sm5 | Nd | Rh | -60 | 2.5 | 0.444 | 88 | 126 | 6 | 7.55 | 4.31 | -0.449 | 0.004 | -1.336 | 0.020 | -0.912 | 0.049 | -0.020 | 0.001 |
| NdRh | oC8 BCr | Nd | Rh | -64 | 2.0 | 0.5 | 20 | 28 | 4 | 8.50 | 4.50 | -0.399 | 0.004 | -1.281 | 0.007 | -0.935 | 0.016 | -0.095 | 0.003 |
| NdRh2 | cF24 Cu2Mg | Nd | Rh | -60 | 1.1 | 0.667 | 16 | 96 | 48 | 8.00 | 6.00 | -0.424 | 0.004 | -0.960 | 0.007 | -0.744 | 0.014 | -0.052 | 0.001 |
| Nd5Ru2 | mC28 B2Pd5 | Nd | Ru | -17 | 1.9 | 0.286 | 112 | 60 | 0 | 7.10 | 3.75 | -0.477 | 0.004 | -1.798 | 0.011 | -1.150 | 0.024 | -0.012 | 0.001 |
| NdRu2 | cF24 Cu2Mg | Nd | Ru | -19 | 1.1 | 0.667 | 16 | 96 | 48 | 8.00 | 6.00 | -0.424 | 0.004 | -1.124 | 0.011 | -0.790 | 0.020 | -0.016 | 0.001 |
| Nd5Sb3 | hP16 Mn5Si3 | Nd | Sb | -108 | 6.4 | 0.375 | 46 | 54 | 6 | 7.30 | 5.50 | -0.464 | 0.004 | -0.498 | 0.009 | -0.523 | 0.013 | -0.041 | 0.002 |
| Nd4Sb3 | cI28 P4Th3 | Nd | Sb | -116 | 6.9 | 0.429 | 40 | 96 | 48 | 5.50 | 8.00 | -0.616 | 0.004 | -0.343 | 0.009 | -0.529 | 0.016 | -0.050 | 0.003 |
| NdSb | cF8 ClNa_1 | Nd | Sb | -123 | 7.3 | 0.5 | 0 | 24 | 0 | 3.00 | 3.00 | -1.129 | 0.004 | -0.913 | 0.009 | -1.234 | 0.021 | -0.213 | 0.013 |
| NdSb2 | oC24 Sb2Sm | Nd | Sb | -91 | 5.4 | 0.667 | 0 | 72 | 20 | 4.50 | 3.50 | -0.753 | 0.004 | -0.783 | 0.009 | -0.872 | 0.017 | -0.105 | 0.006 |
| NdSn3 | cP4 AuCu3_2 | Nd | Sn | -66 | 3.9 | 0.75 | 0 | 12 | 12 | 6.00 | 6.00 | -0.565 | 0.004 | -0.520 | 0.003 | -0.599 | 0.012 | -0.057 | 0.003 |
| NdTl3 | cP4 AuCu3_2 | Tl | Nd | -42 | 2.5 | 0.25 | 12 | 12 | 0 | 6.00 | 6.00 | -0.315 | 0.001 | -0.565 | 0.004 | -0.476 | 0.006 | -0.036 | 0.002 |
| Nd3Zn11 | oI28 Al11La3 | Zn | Nd | -32 | 1.9 | 0.214 | 88 | 96 | 12 | 6.18 | 10.00 | -0.219 | 0.001 | -0.339 | 0.007 | -0.286 | 0.011 | -0.007 | 0.000 |
| NdZn3 | oP16 YZn3 | Zn | Nd | -33 | 1.9 | 0.25 | 36 | 57 | 4 | 5.38 | 8.13 | -0.251 | 0.002 | -0.417 | 0.004 | -0.358 | 0.012 | -0.024 | 0.002 |
| NdZn2 | oI12 CeCu2 | Zn | Nd | -38 | 2.2 | 0.333 | 16 | 48 | 8 | 5.00 | 8.00 | -0.270 | 0.001 | -0.424 | 0.004 | -0.379 | 0.005 | -0.032 | 0.002 |
| NdZn | cP2 ClCs_2 | Zn | Nd | -32 | 1.9 | 0.5 | 0 | 8 | 3 | 4.00 | 7.00 | -0.338 | 0.001 | -0.484 | 0.004 | -0.453 | 0.007 | -0.042 | 0.002 |
| Ni3P | tI32 Ni3P | Ni | P | -61 | 3.6 | 0.25 | 128 | 72 | 0 | 6.83 | 4.50 | -0.652 | 0.015 | -0.729 | 0.007 | -0.761 | 0.060 | -0.070 | 0.004 |
| NiP2 | mC12 NiP2 | Ni | P | -46 | 2.7 | 0.667 | 0 | 16 | 8 | 2.00 | 2.00 | -2.229 | 0.015 | -1.640 | 0.007 | -2.052 | 0.037 | -0.118 | 0.007 |
| NiPr | oC8 BCr | Pr | Ni | -28 | 1.1 | 0.5 | 20 | 28 | 4 | 8.50 | 4.50 | -0.435 | 0.004 | -0.991 | 0.015 | -0.754 | 0.022 | -0.042 | 0.002 |
| Ni5Pr | hP6 CaCu5 | Pr | Ni | -26 | 1.0 | 0.833 | 0 | 18 | 21 | 9.00 | 6.00 | -0.411 | 0.004 | -0.743 | 0.015 | -0.592 | 0.043 | -0.015 | 0.001 |
| NiPt | tP4 AuCu | Ni | Pt | -9 | 0.6 | 0.5 | 4 | 16 | 4 | 6.00 | 6.00 | -0.743 | 0.015 | -0.977 | 0.002 | -0.872 | 0.017 | -0.012 | 0.001 |
| Ni3Sb | oP8 Cu3Ti | Ni | Sb | -18 | 1.1 | 0.25 | 24 | 24 | 0 | 6.00 | 6.00 | -0.743 | 0.015 | -0.457 | 0.009 | -0.615 | 0.039 | -0.016 | 0.001 |
| NiSb | hP4 AsNi | Ni | Sb | -35 | 4.2 | 0.5 | 2 | 12 | 0 | 4.00 | 3.00 | -1.114 | 0.015 | -0.913 | 0.009 | -1.074 | 0.028 | -0.061 | 0.007 |
| NiSb2 | oP6 FeS2 | Ni | Sb | -26 | 1.5 | 0.667 | 0 | 12 | 2 | 3.00 | 2.00 | -1.486 | 0.015 | -1.370 | 0.009 | -1.473 | 0.027 | -0.045 | 0.003 |
| NiSc | cP2 ClCs_2 | Sc | Ni | -45 | 2.3 | 0.5 | 3 | 8 | 0 | 7.00 | 4.00 | -0.559 | 0.007 | -1.114 | 0.015 | -0.895 | 0.022 | -0.058 | 0.003 |
| Ni2Sc | cF24 Cu2Mg | Sc | Ni | -43 | 2.3 | 0.667 | 16 | 96 | 48 | 8.00 | 6.00 | -0.489 | 0.007 | -0.743 | 0.015 | -0.653 | 0.028 | -0.037 | 0.002 |
| Ni31Si12 | hP43 Ni31Si12 | Ni | Si | -43 | 2.6 | 0.279 | 130 | 127 | 0 | 6.23 | 5.27 | -0.715 | 0.019 | -0.885 | 0.041 | -0.804 | 0.095 | -0.004 | 0.000 |
| Ni2Si | oP12 Co2Si | Ni | Si | -43 | 2.5 | 0.333 | 32 | 40 | 0 | 6.50 | 5.00 | -0.686 | 0.015 | -0.933 | 0.041 | -0.854 | 0.040 | -0.044 | 0.003 |
| Ni3Si2 | oC80 Ni3Si2 | Ni | Si | -44 | 2.6 | 0.4 | 170 | 260 | 22 | 6.25 | 4.75 | -0.713 | 0.019 | -0.982 | 0.115 | -0.876 | 0.163 | -0.028 | 0.002 |
| NiSi | oP8 MnP | Ni | Si | -40 | 2.3 | 0.5 | 12 | 24 | 4 | 6.00 | 4.00 | -0.743 | 0.015 | -1.166 | 0.041 | -1.023 | 0.047 | -0.068 | 0.004 |
| NiSi2 | cF12 CaF2 | Ni | Si | -35 | 2.0 | 0.667 | 24 | 32 | 24 | 10.00 | 5.00 | -0.446 | 0.015 | -0.933 | 0.041 | -0.734 | 0.076 | -0.045 | 0.003 |
| NiSm | oC8 BCr | Sm | Ni | -36 | 0.7 | 0.5 | 20 | 28 | 4 | 8.50 | 4.50 | -0.252 | 0.004 | -0.991 | 0.015 | -0.675 | 0.021 | -0.054 | 0.001 |
| Ni5Sm | hP6 CaCu5 | Sm | Ni | -29 | 0.5 | 0.833 | 0 | 18 | 21 | 9.00 | 6.00 | -0.238 | 0.004 | -0.743 | 0.015 | -0.507 | 0.043 | -0.017 | 0.000 |
| Ni3Sn4 | mC14 Ni3Sn4 | Sn | Ni | -34 | 2.0 | 0.429 | 8 | 43 | 8 | 3.69 | 4.92 | -0.847 | 0.230 | -0.907 | 0.043 | -0.893 | 0.246 | -0.016 | 0.002 |
| Ni3Sn2 | oP20 Ni3Sn2 | Sn | Ni | -33 | 4.2 | 0.6 | 16 | 68 | 32 | 6.25 | 5.50 | -0.499 | 0.003 | -0.810 | 0.015 | -0.675 | 0.027 | -0.020 | 0.003 |
| Ni3Sn | hP8 Ni3Sn | Sn | Ni | -24 | 1.4 | 0.75 | 0 | 24 | 24 | 6.00 | 6.00 | -0.520 | 0.003 | -0.743 | 0.015 | -0.652 | 0.039 | -0.021 | 0.001 |
| Ni3Ta | oP8 Cu3Ti | Ni | Ta | -20 | 1.2 | 0.25 | 24 | 24 | 0 | 6.00 | 6.00 | -0.743 | 0.015 | -1.351 | 0.004 | -1.064 | 0.039 | -0.017 | 0.001 |
| Ni2Ta | tI6 MoSi2 | Ni | Ta | -26 | 1.5 | 0.333 | 18 | 20 | 4 | 7.00 | 7.00 | -0.637 | 0.015 | -1.158 | 0.004 | -0.924 | 0.035 | -0.026 | 0.002 |
| NiTa | hR13 Fe7W6 | Ni | Ta | -37 | 2.2 | 0.5 | 15 | 48 | 18 | 6.00 | 6.46 | -0.743 | 0.117 | -1.254 | 0.004 | -1.051 | 0.149 | -0.052 | 0.003 |
| NiTa2 | tI12 Al2Cu | Ni | Ta | -31 | 1.8 | 0.667 | 4 | 32 | 44 | 5.00 | 7.50 | -0.892 | 0.015 | -1.081 | 0.004 | -1.026 | 0.027 | -0.040 | 0.002 |
| Ni3Ti | hP16 Ni3Ti | Ni | Ti | -37 | 2.2 | 0.25 | 48 | 48 | 0 | 6.00 | 6.00 | -0.743 | 0.015 | -0.817 | 0.005 | -0.812 | 0.041 | -0.032 | 0.002 |
| NiTi | cP2 ClCs_1 | Ni | Ti | -34 | 2.0 | 0.5 | 3 | 8 | 3 | 7.00 | 7.00 | -0.637 | 0.015 | -0.700 | 0.005 | -0.713 | 0.025 | -0.044 | 0.003 |
| NiTi2 | cF96 NiTi2 | Ni | Ti | -27 | 1.6 | 0.667 | 48 | 288 | 288 | 6.00 | 6.75 | -0.743 | 0.015 | -0.726 | 0.005 | -0.766 | 0.026 | -0.031 | 0.002 |
| Ni3V | tI8 Al3Ti | Ni | V | -4 | 0.2 | 0.25 | 24 | 24 | 0 | 6.00 | 6.00 | -0.743 | 0.015 | -0.890 | 0.012 | -0.820 | 0.043 | -0.003 | 0.000 |
| Ni2V | oI6 MoPt2 | Ni | V | -6 | 0.3 | 0.333 | 14 | 20 | 2 | 6.00 | 6.00 | -0.743 | 0.015 | -0.890 | 0.012 | -0.823 | 0.037 | -0.006 | 0.000 |
| Ni4W | tI10 MoNi4 | Ni | W | -1 | 0.1 | 0.2 | 36 | 24 | 0 | 6.00 | 6.00 | -0.743 | 0.015 | -1.470 | 0.009 | -1.107 | 0.056 | -0.001 | 0.000 |
| NiY3 | oP16 CFe3 | Y | Ni | -21 | 1.2 | 0.25 | 68 | 34 | 2 | 7.08 | 4.75 | -0.621 | 0.007 | -0.938 | 0.101 | -0.805 | 0.143 | -0.025 | 0.003 |
| Ni2Y3 | tP80 Ni2Y3 | Y | Ni | -33 | 2.0 | 0.4 | 256 | 228 | 24 | 7.71 | 4.31 | -0.571 | 0.004 | -1.034 | 0.015 | -0.826 | 0.038 | -0.024 | 0.002 |
| NiY | oP8 BFe | Y | Ni | -33 | 2.0 | 0.5 | 20 | 28 | 4 | 8.50 | 4.50 | -0.518 | 0.004 | -0.991 | 0.015 | -0.804 | 0.023 | -0.049 | 0.003 |
| Ni2Y | cF24 Cu2Mg | Y | Ni | -32 | 1.9 | 0.667 | 16 | 96 | 48 | 8.00 | 6.00 | -0.550 | 0.004 | -0.743 | 0.015 | -0.674 | 0.026 | -0.028 | 0.002 |
| Ni3Y | hR12 Be3Nb | Y | Ni | -32 | 1.9 | 0.75 | 5 | 42 | 33 | 8.67 | 6.00 | -0.508 | 0.004 | -0.743 | 0.015 | -0.649 | 0.033 | -0.024 | 0.001 |
| Ni7Y2 | hR18 Co7Er2 | Y | Ni | -31 | 1.9 | 0.778 | 6 | 60 | 54 | 9.00 | 6.00 | -0.489 | 0.004 | -0.743 | 0.015 | -0.627 | 0.036 | -0.011 | 0.001 |
| Ni5Y | hP6 CaCu5 | Y | Ni | -30 | 1.8 | 0.833 | 0 | 18 | 21 | 9.00 | 6.00 | -0.489 | 0.004 | -0.743 | 0.015 | -0.633 | 0.043 | -0.017 | 0.001 |
| Ni17Y2 | hP38 Ni17Th2 | Y | Ni | -18 | 1.1 | 0.895 | 0 | 76 | 170 | 9.50 | 6.12 | -0.463 | 0.004 | -0.729 | 0.015 | -0.601 | 0.130 | -0.005 | 0.000 |
| Ni5Zr | cF24 AuBe5 | Ni | Zr | -30 | 1.8 | 0.167 | 96 | 64 | 0 | 6.40 | 8.00 | -0.697 | 0.015 | -0.790 | 0.015 | -0.763 | 0.056 | -0.020 | 0.001 |
| Ni7Zr2 | mC36 Ni7Zr2 | Ni | Zr | -38 | 2.2 | 0.222 | 112 | 112 | 8 | 6.00 | 8.00 | -0.743 | 0.015 | -0.790 | 0.015 | -0.781 | 0.044 | -0.014 | 0.001 |
| Ni21Zr8 | aP29 Hf8Ni21 | Ni | Zr | -41 | 2.4 | 0.276 | 80 | 96 | 6 | 6.07 | 6.75 | -0.734 | 0.023 | -0.937 | 0.015 | -0.840 | 0.082 | -0.004 | 0.000 |
| Ni10Zr7 | oC68 Ni10Zr7 | Ni | Zr | -46 | 2.7 | 0.412 | 84 | 254 | 88 | 5.28 | 7.68 | -0.845 | 0.020 | -0.823 | 0.020 | -0.842 | 0.070 | -0.008 | 0.001 |
| Ni11Zr9 | tI40 Pt11Zr9 | Ni | Zr | -46 | 2.7 | 0.45 | 62 | 154 | 48 | 6.32 | 6.94 | -0.706 | 0.077 | -0.910 | 0.015 | -0.814 | 0.133 | -0.006 | 0.000 |
| NiZr | oC8 BCr | Ni | Zr | -48 | 2.8 | 0.5 | 4 | 28 | 20 | 4.50 | 8.50 | -0.991 | 0.015 | -0.744 | 0.015 | -0.938 | 0.041 | -0.071 | 0.004 |
| NiZr2 | tI12 Al2Cu | Ni | Zr | -35 | 2.1 | 0.667 | 4 | 32 | 44 | 5.00 | 7.50 | -0.892 | 0.015 | -0.843 | 0.015 | -0.913 | 0.057 | -0.046 | 0.003 |
| O3Sc2 | cI80 Mn2O3 | Sc | O | -382 | 22.6 | 0.6 | 0 | 192 | 0 | 3.00 | 2.00 | -1.305 | 0.007 | -1.292 | 0.000 | -1.628 | 0.026 | -0.330 | 0.020 |
| O2Si | hP9 O2Si | Si | O | -304 | 18.0 | 0.667 | 0 | 12 | 0 | 2.00 | 1.00 | -2.332 | 0.041 | -2.583 | 0.000 | -3.244 | 0.067 | -0.787 | 0.047 |
| O3Y2 | cI80 Mn2O3 | Y | O | -387 | 22.9 | 0.6 | 0 | 192 | 0 | 3.00 | 2.00 | -1.467 | 0.004 | -1.292 | 0.000 | -1.714 | 0.023 | -0.334 | 0.020 |
| O2Zr | mP12 O2Zr | Zr | O | -367 | 21.8 | 0.667 | 0 | 28 | 0 | 3.50 | 1.75 | -1.806 | 0.015 | -1.476 | 0.000 | -2.185 | 0.045 | -0.543 | 0.032 |
| OsP2 | oP6 FeS2 | Os | P | -44 | 2.6 | 0.667 | 0 | 12 | 2 | 3.00 | 2.00 | -2.719 | 0.011 | -1.640 | 0.007 | -2.256 | 0.020 | -0.076 | 0.005 |
| Os2Pu | hP12 MgZn2 | Pu | Os | -20 | 1.2 | 0.667 | 8 | 48 | 24 | 8.00 | 6.00 | -0.447 | 0.010 | -1.359 | 0.011 | -0.920 | 0.025 | -0.017 | 0.001 |
| OsSi | cP8 FeSi | Os | Si | -25 | 1.5 | 0.5 | 12 | 28 | 12 | 6.50 | 6.50 | -1.255 | 0.011 | -0.718 | 0.041 | -1.024 | 0.046 | -0.038 | 0.002 |
| OsSi2 | oC48 FeSi2 | Os | Si | -29 | 1.7 | 0.667 | 16 | 128 | 80 | 5.00 | 4.50 | -1.631 | 0.011 | -1.036 | 0.041 | -1.371 | 0.058 | -0.037 | 0.002 |
| OsTi | cP2 ClCs_1 | Ti | Os | -69 | 3.6 | 0.5 | 3 | 8 | 3 | 7.00 | 7.00 | -0.700 | 0.005 | -1.165 | 0.011 | -1.022 | 0.023 | -0.089 | 0.005 |
| Os2Y | hP12 MgZn2 | Y | Os | -25 | 2.8 | 0.667 | 8 | 48 | 24 | 8.00 | 6.00 | -0.550 | 0.004 | -1.359 | 0.011 | -0.976 | 0.021 | -0.021 | 0.002 |
| PSi | oC48 PSi | P | Si | -31 | 1.8 | 0.5 | 0 | 72 | 12 | 1.50 | 2.00 | -2.187 | 0.007 | -2.332 | 0.041 | -2.365 | 0.044 | -0.106 | 0.006 |
| Pb2Pd | tI12 Al2Cu | Pb | Pd | -25 | 1.5 | 0.333 | 44 | 32 | 4 | 7.50 | 5.00 | -0.270 | 0.001 | -0.781 | 0.004 | -0.557 | 0.009 | -0.032 | 0.002 |
| Pb9Pd13 | mC88 Pb9Pd13 | Pb | Pd | -40 | 2.4 | 0.591 | 58 | 356 | 152 | 6.56 | 6.35 | -0.309 | 0.063 | -0.615 | 0.004 | -0.466 | 0.056 | -0.005 | 0.000 |
| Pb3Pd5 | mc32 Ge3Ni5 | Pb | Pd | -39 | 2.3 | 0.625 | 0 | 89 | 59 | 3.71 | 5.18 | -0.546 | 0.043 | -0.754 | 0.024 | -0.668 | 0.084 | -0.018 | 0.003 |
| PbPd3 | cP4 AuCu3_2 | Pb | Pd | -36 | 2.1 | 0.75 | 0 | 12 | 12 | 6.00 | 6.00 | -0.337 | 0.001 | -0.651 | 0.004 | -0.525 | 0.012 | -0.031 | 0.002 |
| Pb3Pr | cP4 AuCu3_2 | Pb | Pr | -53 | 3.1 | 0.25 | 12 | 12 | 0 | 6.00 | 6.00 | -0.337 | 0.001 | -0.617 | 0.004 | -0.522 | 0.008 | -0.045 | 0.003 |
| Pb3Sm | cP4 AuCu3_2 | Pb | Sm | -50 | 2.9 | 0.25 | 12 | 12 | 0 | 6.00 | 6.00 | -0.337 | 0.001 | -0.357 | 0.004 | -0.390 | 0.008 | -0.043 | 0.003 |
| Pb3Y | cP4 AuCu3_2 | Pb | Y | -33 | 1.9 | 0.25 | 12 | 12 | 0 | 6.00 | 6.00 | -0.337 | 0.001 | -0.734 | 0.004 | -0.564 | 0.007 | -0.028 | 0.002 |
| Pb2Y | oC12 Si2Zr | Pb | Y | -40 | 2.4 | 0.333 | 12 | 40 | 12 | 4.00 | 8.00 | -0.506 | 0.001 | -0.550 | 0.004 | -0.570 | 0.008 | -0.042 | 0.002 |
| Pb4Y5 | oP36 Ge4Sm5 | Pb | Y | -60 | 3.6 | 0.556 | 6 | 126 | 88 | 4.31 | 7.55 | -0.469 | 0.020 | -0.583 | 0.004 | -0.546 | 0.025 | -0.020 | 0.001 |
| Pb3Y5 | hP16 Mn5Si3 | Pb | Y | -67 | 4.0 | 0.625 | 6 | 54 | 46 | 5.50 | 7.30 | -0.368 | 0.001 | -0.603 | 0.004 | -0.511 | 0.010 | -0.026 | 0.002 |
| Pb3Yb | cP4 AuCu3_2 | Yb | Pb | -37 | 2.2 | 0.75 | 0 | 12 | 12 | 6.00 | 6.00 | -0.269 | 0.004 | -0.337 | 0.001 | -0.335 | 0.007 | -0.032 | 0.002 |
| PdSb | hP4 AsNi | Pd | Sb | -47 | 2.8 | 0.5 | 2 | 12 | 0 | 4.00 | 3.00 | -0.976 | 0.004 | -0.913 | 0.009 | -1.026 | 0.013 | -0.081 | 0.005 |
| PdSc | cP2 ClCs_1 | Pd | Sc | -101 | 8.6 | 0.5 | 3 | 8 | 3 | 7.00 | 7.00 | -0.558 | 0.004 | -0.559 | 0.007 | -0.690 | 0.023 | -0.131 | 0.011 |
| Pd3Si | oP16 CFe3 | Pd | Si | -58 | 0.3 | 0.25 | 68 | 34 | 2 | 7.08 | 4.75 | -0.551 | 0.006 | -0.982 | 0.089 | -0.837 | 0.143 | -0.070 | 0.004 |
| Pd2Si | hP9 Fe2P | Pd | Si | -65 | 3.9 | 0.333 | 29 | 28 | 0 | 7.06 | 4.63 | -0.553 | 0.025 | -1.008 | 0.041 | -0.853 | 0.096 | -0.073 | 0.006 |
| Pd3Sm7 | hP20 Fe3Th7 | Sm | Pd | -82 | 4.9 | 0.3 | 78 | 53 | 0 | 7.45 | 4.38 | -0.288 | 0.004 | -0.892 | 0.008 | -0.622 | 0.033 | -0.033 | 0.003 |
| PdSm | oC8 BCr | Sm | Pd | -88 | 5.2 | 0.5 | 20 | 28 | 4 | 8.50 | 4.50 | -0.252 | 0.004 | -0.867 | 0.004 | -0.690 | 0.017 | -0.130 | 0.008 |
| Pd4Sm3 | hR14 Pd4Pu3 | Sm | Pd | -86 | 5.1 | 0.571 | 21 | 54 | 20 | 8.00 | 5.88 | -0.268 | 0.004 | -0.664 | 0.017 | -0.499 | 0.052 | -0.033 | 0.002 |
| Pd3Sm | cP4 AuCu3_1 | Sm | Pd | -79 | 4.7 | 0.75 | 3 | 12 | 12 | 9.00 | 6.00 | -0.238 | 0.004 | -0.651 | 0.004 | -0.512 | 0.016 | -0.068 | 0.004 |
| PdSn4 | oC20 PtSn4 | Sn | Pd | -29 | 1.7 | 0.2 | 80 | 32 | 0 | 6.00 | 4.00 | -0.520 | 0.003 | -0.976 | 0.004 | -0.785 | 0.020 | -0.037 | 0.002 |
| PdSn3 | oC32 PdSn3 | Sn | Pd | -35 | 2.1 | 0.25 | 124 | 64 | 4 | 6.50 | 4.50 | -0.480 | 0.003 | -0.867 | 0.004 | -0.719 | 0.017 | -0.045 | 0.003 |
| PdSn2 | oC24 PdSn2 | Sn | Pd | -44 | 2.6 | 0.333 | 52 | 64 | 4 | 5.25 | 4.50 | -0.595 | 0.003 | -0.867 | 0.004 | -0.788 | 0.013 | -0.057 | 0.003 |
| PdSn | oP8 BFe | Sn | Pd | -61 | 3.6 | 0.5 | 20 | 28 | 4 | 8.50 | 4.50 | -0.367 | 0.003 | -0.867 | 0.004 | -0.708 | 0.013 | -0.090 | 0.005 |
| Pd3Sn2 | hP6 InNi2 | Sn | Pd | -65 | 3.8 | 0.6 | 0 | 22 | 14 | 4.58 | 6.94 | -0.681 | 0.003 | -0.562 | 0.004 | -0.658 | 0.010 | -0.037 | 0.002 |
| Pd2Sn | oP12 Co2Si | Sn | Pd | -64 | 3.8 | 0.667 | 0 | 40 | 32 | 5.00 | 6.50 | -0.624 | 0.003 | -0.600 | 0.004 | -0.679 | 0.013 | -0.067 | 0.004 |
| Pd3Sn | cP4 AuCu3_2 | Sn | Pd | -59 | 3.5 | 0.75 | 0 | 12 | 12 | 6.00 | 6.00 | -0.520 | 0.003 | -0.651 | 0.004 | -0.636 | 0.013 | -0.051 | 0.003 |
| Pd3Ta | tI8 Al3Ti | Pd | Ta | -36 | 2.0 | 0.25 | 24 | 24 | 0 | 6.00 | 6.00 | -0.651 | 0.004 | -1.351 | 0.004 | -1.032 | 0.013 | -0.031 | 0.002 |
| Pd3Ti | cP4 AuCu3_2 | Pd | Ti | -64 | 1.4 | 0.25 | 12 | 12 | 0 | 6.00 | 6.00 | -0.651 | 0.004 | -0.817 | 0.005 | -0.789 | 0.013 | -0.055 | 0.001 |
| PdTi | oP4 AuCd | Pd | Ti | -60 | 3.1 | 0.5 | 4 | 16 | 4 | 6.00 | 6.00 | -0.651 | 0.004 | -0.817 | 0.005 | -0.812 | 0.013 | -0.078 | 0.004 |
| Pd3U | hP16 Ni3Ti | Pd | U | -92 | 4.7 | 0.25 | 48 | 48 | 0 | 6.00 | 6.00 | -0.651 | 0.004 | -0.921 | 0.014 | -0.865 | 0.020 | -0.080 | 0.004 |
| Pd3Y | cP4 AuCu3_2 | Pd | Y | -79 | 6.5 | 0.25 | 12 | 12 | 0 | 6.00 | 6.00 | -0.651 | 0.004 | -0.734 | 0.004 | -0.760 | 0.016 | -0.068 | 0.006 |
| Pd3Zr | hP16 Ni3Ti | Pd | Zr | -84 | 2.7 | 0.25 | 48 | 48 | 0 | 6.00 | 6.00 | -0.651 | 0.004 | -1.054 | 0.015 | -0.924 | 0.019 | -0.072 | 0.002 |
| PdZr | oC8 BCr | Pd | Zr | -69 | 3.2 | 0.5 | 4 | 28 | 20 | 4.50 | 8.50 | -0.867 | 0.004 | -0.744 | 0.015 | -0.907 | 0.031 | -0.102 | 0.005 |
| PdZr2 | tI6 MoSi2 | Pd | Zr | -57 | 3.4 | 0.667 | 4 | 20 | 18 | 7.00 | 7.00 | -0.558 | 0.004 | -0.903 | 0.015 | -0.789 | 0.037 | -0.059 | 0.004 |
| PrPt | oP8 BFe | Pr | Pt | -103 | 2.7 | 0.5 | 20 | 28 | 4 | 8.50 | 4.50 | -0.435 | 0.004 | -1.303 | 0.002 | -1.021 | 0.012 | -0.152 | 0.004 |
| PrPt2 | cF24 Cu2Mg | Pr | Pt | -94 | 2.4 | 0.667 | 16 | 96 | 48 | 8.00 | 6.00 | -0.462 | 0.004 | -0.977 | 0.002 | -0.801 | 0.008 | -0.081 | 0.002 |
| PrRh2 | cF24 Cu2Mg | Pr | Rh | -60 | 1.7 | 0.667 | 16 | 96 | 48 | 8.00 | 6.00 | -0.462 | 0.004 | -0.960 | 0.007 | -0.764 | 0.014 | -0.052 | 0.001 |
| PrRu2 | cF24 Cu2Mg | Pr | Ru | -17 | 1.5 | 0.667 | 16 | 96 | 48 | 8.00 | 6.00 | -0.462 | 0.004 | -1.124 | 0.011 | -0.808 | 0.020 | -0.015 | 0.001 |
| Pr2Sb | tI12 La2Sb | Pr | Sb | -102 | 6.0 | 0.333 | 40 | 36 | 0 | 7.25 | 4.50 | -0.510 | 0.004 | -0.609 | 0.009 | -0.677 | 0.019 | -0.117 | 0.007 |
| Pr5Sb3 | hP16 Mn5Si3 | Pr | Sb | -112 | 6.6 | 0.375 | 46 | 54 | 6 | 7.30 | 5.50 | -0.507 | 0.004 | -0.498 | 0.009 | -0.545 | 0.014 | -0.043 | 0.003 |
| Pr4Sb3 | cI28 P4Th3 | Pr | Sb | -120 | 7.1 | 0.429 | 40 | 96 | 48 | 5.50 | 8.00 | -0.673 | 0.004 | -0.343 | 0.009 | -0.559 | 0.016 | -0.052 | 0.003 |
| PrSb | cF8 ClNa_1 | Pr | Sb | -129 | 7.6 | 0.5 | 0 | 24 | 0 | 3.00 | 3.00 | -1.233 | 0.004 | -0.913 | 0.009 | -1.295 | 0.021 | -0.222 | 0.013 |
| PrSb2 | oC24 Sb2Sm | Pr | Sb | -98 | 5.8 | 0.667 | 0 | 72 | 20 | 4.50 | 3.50 | -0.822 | 0.004 | -0.783 | 0.009 | -0.915 | 0.017 | -0.112 | 0.007 |
| PrSi | oP8 BFe | Pr | Si | -78 | 4.6 | 0.5 | 20 | 28 | 4 | 8.50 | 4.50 | -0.435 | 0.004 | -1.036 | 0.041 | -0.851 | 0.030 | -0.116 | 0.007 |
| PrSi2 | oI12 Gd2Si3 | Pr | Si | -62 | 3.6 | 0.667 | 16 | 42 | 9 | 9.25 | 3.75 | -0.400 | 0.004 | -1.244 | 0.041 | -0.883 | 0.032 | -0.061 | 0.004 |
| PrSn3 | cP4 AuCu3_2 | Pr | Sn | -56 | 3.3 | 0.75 | 0 | 12 | 12 | 6.00 | 6.00 | -0.617 | 0.004 | -0.520 | 0.003 | -0.617 | 0.012 | -0.048 | 0.003 |
| PrTl3 | cP4 AuCu3_2 | Tl | Pr | -46 | 2.7 | 0.25 | 12 | 12 | 0 | 6.00 | 6.00 | -0.315 | 0.001 | -0.617 | 0.004 | -0.505 | 0.006 | -0.040 | 0.002 |
| Pt2Pu | cF24 Cu2Mg | Pu | Pt | -63 | 3.7 | 0.667 | 16 | 96 | 48 | 8.00 | 6.00 | -0.447 | 0.010 | -0.977 | 0.002 | -0.766 | 0.014 | -0.054 | 0.003 |
| Pt3Pu | cP4 AuCu3_2 | Pu | Pt | -55 | 3.3 | 0.75 | 0 | 12 | 12 | 6.00 | 6.00 | -0.596 | 0.010 | -0.977 | 0.002 | -0.834 | 0.014 | -0.048 | 0.003 |
| Pt4Pu | oC10 Pt4Pu | Pu | Pt | -49 | 2.9 | 0.8 | 0 | 28 | 26 | 7.00 | 5.00 | -0.511 | 0.010 | -1.173 | 0.002 | -0.878 | 0.013 | -0.036 | 0.002 |
| Pt5Pu | hP6 CaCu5 | Pu | Pt | -44 | 2.6 | 0.833 | 0 | 18 | 21 | 9.00 | 6.00 | -0.397 | 0.010 | -0.977 | 0.002 | -0.713 | 0.012 | -0.025 | 0.002 |
| PtSc | cP2 ClCs_1 | Sc | Pt | -105 | 5.4 | 0.5 | 3 | 8 | 3 | 7.00 | 7.00 | -0.559 | 0.007 | -0.838 | 0.002 | -0.834 | 0.017 | -0.136 | 0.007 |
| Pt3Sc | cP4 AuCu3_2 | Sc | Pt | -95 | 2.0 | 0.75 | 0 | 12 | 12 | 6.00 | 6.00 | -0.653 | 0.007 | -0.977 | 0.002 | -0.897 | 0.011 | -0.082 | 0.002 |
| Pt2Si | tI6 H2Th | Pt | Si | -62 | 3.7 | 0.333 | 12 | 16 | 0 | 5.00 | 4.00 | -1.173 | 0.002 | -1.166 | 0.041 | -1.250 | 0.020 | -0.080 | 0.005 |
| PtSi | oP8 MnP | Pt | Si | -59 | 3.5 | 0.5 | 12 | 24 | 4 | 6.00 | 4.00 | -0.977 | 0.002 | -1.166 | 0.041 | -1.173 | 0.030 | -0.102 | 0.006 |
| PtSm | oP8 BFe | Sm | Pt | -101 | 3.5 | 0.5 | 20 | 28 | 4 | 8.50 | 4.50 | -0.252 | 0.004 | -1.303 | 0.002 | -0.927 | 0.013 | -0.150 | 0.005 |
| Pt2Sm | cF24 Cu2Mg | Sm | Pt | -100 | 2.6 | 0.667 | 16 | 96 | 48 | 8.00 | 6.00 | -0.268 | 0.004 | -0.977 | 0.002 | -0.709 | 0.008 | -0.087 | 0.002 |
| Pt2Sn3 | hP10 Pt2Sn3 | Sn | Pt | -54 | 3.2 | 0.4 | 18 | 28 | 2 | 5.33 | 4.00 | -0.585 | 0.003 | -1.466 | 0.002 | -1.066 | 0.010 | -0.040 | 0.002 |
| PtSn | hP4 AsNi | Sn | Pt | -59 | 3.5 | 0.5 | 2 | 12 | 0 | 4.00 | 3.00 | -0.780 | 0.003 | -1.954 | 0.002 | -1.469 | 0.011 | -0.102 | 0.006 |
| Pt3Sn | cP4 AuCu3_2 | Sn | Pt | -48 | 2.8 | 0.75 | 0 | 12 | 12 | 6.00 | 6.00 | -0.520 | 0.003 | -0.977 | 0.002 | -0.790 | 0.009 | -0.041 | 0.002 |
| PtTb | oP8 BFe | Tb | Pt | -101 | 2.9 | 0.5 | 20 | 28 | 4 | 8.50 | 4.50 | -0.474 | 0.004 | -1.303 | 0.002 | -1.038 | 0.012 | -0.150 | 0.004 |
| Pt2Tb | cF24 Cu2Mg | Tb | Pt | -97 | 3.1 | 0.667 | 16 | 96 | 48 | 8.00 | 6.00 | -0.504 | 0.004 | -0.977 | 0.002 | -0.824 | 0.008 | -0.084 | 0.003 |
| Pt3Tb | cP4 AuCu3_2 | Tb | Pt | -86 | 2.9 | 0.75 | 0 | 12 | 12 | 6.00 | 6.00 | -0.671 | 0.004 | -0.977 | 0.002 | -0.898 | 0.010 | -0.074 | 0.003 |
| PtTi | oP4 AuCd | Ti | Pt | -84 | 3.0 | 0.5 | 4 | 16 | 4 | 6.00 | 6.00 | -0.817 | 0.005 | -0.977 | 0.002 | -1.006 | 0.011 | -0.109 | 0.004 |
| PtTm | oP8 BFe | Tm | Pt | -121 | 2.0 | 0.5 | 20 | 28 | 4 | 8.50 | 4.50 | -0.283 | 0.004 | -1.303 | 0.002 | -0.972 | 0.011 | -0.179 | 0.003 |
| Pt2Tm | cF24 Cu2Mg | Tm | Pt | -112 | 3.1 | 0.667 | 16 | 96 | 48 | 8.00 | 6.00 | -0.301 | 0.004 | -0.977 | 0.002 | -0.736 | 0.008 | -0.097 | 0.003 |
| Pt3Tm | cP4 AuCu3_2 | Tm | Pt | -91 | 2.1 | 0.75 | 0 | 12 | 12 | 6.00 | 6.00 | -0.401 | 0.004 | -0.977 | 0.002 | -0.768 | 0.009 | -0.079 | 0.002 |
| Pt3V | tI8 Al3Ti | V | Pt | -27 | 3.0 | 0.75 | 0 | 24 | 24 | 6.00 | 6.00 | -0.890 | 0.012 | -0.977 | 0.002 | -0.957 | 0.015 | -0.024 | 0.003 |
| PtY | oP8 BFe | Y | Pt | -104 | 2.3 | 0.5 | 20 | 28 | 4 | 8.50 | 4.50 | -0.518 | 0.004 | -1.303 | 0.002 | -1.064 | 0.011 | -0.154 | 0.003 |
| Pt3Y | cP4 AuCu3_2 | Y | Pt | -87 | 2.0 | 0.75 | 0 | 12 | 12 | 6.00 | 6.00 | -0.734 | 0.004 | -0.977 | 0.002 | -0.930 | 0.009 | -0.075 | 0.002 |
| Pt3Zr | cP4 AuCu3_2 | Pt | Zr | -128 | 7.6 | 0.25 | 12 | 12 | 0 | 6.00 | 6.00 | -0.977 | 0.002 | -1.054 | 0.015 | -1.126 | 0.019 | -0.111 | 0.007 |
| PtZr | oC8 BCr | Pt | Zr | -97 | 5.9 | 0.5 | 4 | 28 | 20 | 4.50 | 8.50 | -1.303 | 0.002 | -0.744 | 0.015 | -1.167 | 0.034 | -0.144 | 0.009 |
| PuRh2 | cF24 Cu2Mg | Pu | Rh | -60 | 3.6 | 0.667 | 16 | 96 | 48 | 8.00 | 6.00 | -0.447 | 0.010 | -0.960 | 0.007 | -0.756 | 0.021 | -0.052 | 0.003 |
| PuRh3 | cP4 AuCu3_1 | Pu | Rh | -50 | 3.0 | 0.75 | 3 | 12 | 12 | 9.00 | 6.00 | -0.397 | 0.010 | -0.960 | 0.007 | -0.722 | 0.028 | -0.043 | 0.003 |
| PuRu2 | cF24 Cu2Mg | Pu | Ru | -41 | 2.4 | 0.667 | 16 | 96 | 48 | 8.00 | 6.00 | -0.447 | 0.010 | -1.124 | 0.011 | -0.821 | 0.026 | -0.035 | 0.002 |
| ReSi2 | tI6 MoSi2 | Re | Si | -30 | 1.8 | 0.667 | 4 | 20 | 18 | 7.00 | 7.00 | -1.146 | 0.011 | -0.666 | 0.041 | -0.937 | 0.064 | -0.031 | 0.002 |
| Rh4Sm5 | oP36 Ge4Sm5 | Sm | Rh | -67 | 1.0 | 0.444 | 88 | 126 | 6 | 7.55 | 4.31 | -0.284 | 0.004 | -1.336 | 0.012 | -0.832 | 0.047 | -0.022 | 0.001 |
| Rh2Sm | cF24 Cu2Mg | Sm | Rh | -66 | 1.2 | 0.667 | 16 | 96 | 48 | 8.00 | 6.00 | -0.268 | 0.004 | -0.960 | 0.007 | -0.671 | 0.014 | -0.057 | 0.001 |
| RhTb | cP2 ClCs_2 | Tb | Rh | -72 | 1.1 | 0.5 | 3 | 8 | 0 | 7.00 | 4.00 | -0.576 | 0.004 | -1.441 | 0.007 | -1.102 | 0.011 | -0.094 | 0.001 |
| Rh2Tb | cF24 Cu2Mg | Tb | Rh | -64 | 1.5 | 0.667 | 16 | 96 | 48 | 8.00 | 6.00 | -0.504 | 0.004 | -0.960 | 0.007 | -0.788 | 0.014 | -0.056 | 0.001 |
| RhTi | tP4 AuCu | Ti | Rh | -73 | 2.9 | 0.5 | 4 | 16 | 4 | 6.00 | 6.00 | -0.817 | 0.005 | -0.960 | 0.007 | -0.984 | 0.016 | -0.095 | 0.004 |
| Rh3U | cP4 AuCu3_2 | U | Rh | -70 | 2.2 | 0.75 | 0 | 12 | 12 | 6.00 | 6.00 | -0.921 | 0.014 | -0.960 | 0.007 | -1.001 | 0.026 | -0.060 | 0.002 |
| RhY | cP2 ClCs_2 | Y | Rh | -77 | 3.4 | 0.5 | 3 | 8 | 0 | 7.00 | 4.00 | -0.629 | 0.004 | -1.441 | 0.007 | -1.134 | 0.014 | -0.099 | 0.004 |
| Rh2Y | cF24 Cu2Mg | Y | Rh | -65 | 1.3 | 0.667 | 16 | 96 | 48 | 8.00 | 6.00 | -0.550 | 0.004 | -0.960 | 0.007 | -0.812 | 0.014 | -0.056 | 0.001 |
| RhZr | oP8 BFe | Rh | Zr | -77 | 3.1 | 0.5 | 4 | 28 | 20 | 4.50 | 8.50 | -1.281 | 0.007 | -0.744 | 0.015 | -1.126 | 0.034 | -0.114 | 0.005 |
| RuSc | cP2 ClCs_2 | Sc | Ru | -45 | 2.8 | 0.5 | 3 | 8 | 0 | 7.00 | 4.00 | -0.559 | 0.007 | -1.686 | 0.011 | -1.180 | 0.020 | -0.058 | 0.004 |
| RuSi | cP8 FeSi | Ru | Si | -53 | 3.1 | 0.5 | 12 | 28 | 12 | 6.50 | 6.50 | -1.037 | 0.011 | -0.718 | 0.041 | -0.956 | 0.048 | -0.078 | 0.005 |
| Ru2Tb5 | mC28 B2Pd5 | Tb | Ru | -30 | 1.9 | 0.286 | 112 | 60 | 0 | 7.10 | 3.75 | -0.567 | 0.004 | -1.798 | 0.011 | -1.203 | 0.024 | -0.021 | 0.001 |
| Ru2Tb | hP12 MgZn2 | Tb | Ru | -24 | 1.7 | 0.667 | 8 | 48 | 24 | 8.00 | 6.00 | -0.504 | 0.004 | -1.124 | 0.011 | -0.834 | 0.020 | -0.020 | 0.001 |
| RuTi | cP2 ClCs_1 | Ti | Ru | -77 | 3.7 | 0.5 | 3 | 8 | 3 | 7.00 | 7.00 | -0.700 | 0.005 | -0.963 | 0.011 | -0.932 | 0.023 | -0.100 | 0.005 |
| Ru3U | cP4 AuCu3_2 | U | Ru | -39 | 1.2 | 0.75 | 0 | 12 | 12 | 6.00 | 6.00 | -0.921 | 0.014 | -1.124 | 0.011 | -1.056 | 0.035 | -0.034 | 0.001 |
| Ru2Y5 | mC28 B2Pd5 | Y | Ru | -27 | 3.1 | 0.286 | 112 | 60 | 0 | 7.10 | 3.75 | -0.620 | 0.004 | -1.798 | 0.011 | -1.228 | 0.025 | -0.019 | 0.002 |
| Ru2Y | hP12 MgZn2 | Y | Ru | -20 | 1.2 | 0.667 | 8 | 48 | 24 | 8.00 | 6.00 | -0.550 | 0.004 | -1.124 | 0.011 | -0.854 | 0.020 | -0.017 | 0.001 |
| Ru2Zr | hP12 MgZn2 | Zr | Ru | -37 | 2.2 | 0.667 | 8 | 48 | 24 | 8.00 | 6.00 | -0.790 | 0.015 | -1.124 | 0.011 | -0.989 | 0.028 | -0.032 | 0.002 |
| SbSm2 | tI12 La2Sb | Sm | Sb | -102 | 6.0 | 0.333 | 40 | 36 | 0 | 7.25 | 4.50 | -0.295 | 0.004 | -0.609 | 0.009 | -0.570 | 0.019 | -0.117 | 0.007 |
| Sb3Sm5 | hP16 Mn5Si3 | Sm | Sb | -112 | 6.6 | 0.375 | 46 | 54 | 6 | 7.30 | 5.50 | -0.293 | 0.004 | -0.498 | 0.009 | -0.439 | 0.014 | -0.043 | 0.003 |
| Sb3Sm4 | cI28 P4Th3 | Sm | Sb | -118 | 7.0 | 0.429 | 40 | 96 | 48 | 5.50 | 8.00 | -0.390 | 0.004 | -0.343 | 0.009 | -0.417 | 0.016 | -0.051 | 0.003 |
| SbSm | cF8 ClNa_1 | Sm | Sb | -122 | 7.2 | 0.5 | 0 | 24 | 0 | 3.00 | 3.00 | -0.714 | 0.004 | -0.913 | 0.009 | -1.025 | 0.020 | -0.211 | 0.012 |
| Sb2Sm | oC24 Sb2Sm | Sm | Sb | -90 | 5.3 | 0.667 | 0 | 72 | 20 | 4.50 | 3.50 | -0.476 | 0.004 | -0.783 | 0.009 | -0.733 | 0.017 | -0.103 | 0.006 |
| SbSr2 | tI12 La2Sb | Sr | Sb | -109 | 4.2 | 0.333 | 40 | 36 | 0 | 7.25 | 4.50 | -0.234 | 0.003 | -0.609 | 0.009 | -0.547 | 0.015 | -0.125 | 0.005 |
| SbTb | cF8 ClNa_1 | Tb | Sb | -17 | 1.0 | 0.5 | 0 | 24 | 0 | 3.00 | 3.00 | -1.343 | 0.004 | -0.913 | 0.009 | -1.158 | 0.010 | -0.029 | 0.002 |
| SbY3 | tP32 PTi3 | Y | Sb | -64 | 3.8 | 0.25 | 128 | 72 | 0 | 6.83 | 4.50 | -0.644 | 0.031 | -0.609 | 0.009 | -0.700 | 0.142 | -0.074 | 0.004 |
| Sb3Y5 | hP16 Mn5Si3 | Y | Sb | -96 | 5.7 | 0.375 | 46 | 54 | 6 | 7.30 | 5.50 | -0.603 | 0.004 | -0.498 | 0.009 | -0.588 | 0.013 | -0.037 | 0.002 |
| SbY | cF8 ClNa_1 | Y | Sb | -108 | 6.4 | 0.5 | 0 | 24 | 0 | 3.00 | 3.00 | -1.467 | 0.004 | -0.913 | 0.009 | -1.377 | 0.019 | -0.186 | 0.011 |
| SbYb | cF8 ClNa_1 | Yb | Sb | -62 | 3.7 | 0.5 | 0 | 24 | 0 | 3.00 | 3.00 | -0.538 | 0.004 | -0.913 | 0.009 | -0.833 | 0.014 | -0.107 | 0.006 |
| SbZn | oP16 CdSb | Zn | Sb | -12 | 0.7 | 0.5 | 4 | 32 | 4 | 2.50 | 2.50 | -0.541 | 0.001 | -1.096 | 0.009 | -0.848 | 0.010 | -0.030 | 0.002 |
| Sc5Si3 | hP16 Mn5Si3 | Sc | Si | -125 | 7.4 | 0.375 | 46 | 54 | 6 | 7.30 | 5.50 | -0.536 | 0.007 | -0.848 | 0.041 | -0.740 | 0.030 | -0.048 | 0.003 |
| ScSi | oC8 BCr | Sc | Si | -117 | 6.9 | 0.5 | 20 | 28 | 4 | 8.50 | 4.50 | -0.461 | 0.007 | -1.036 | 0.041 | -0.922 | 0.039 | -0.173 | 0.010 |
| Sc3Si5 | hP3 AlB2 | Sc | Si | -71 | 4.2 | 0.625 | 4 | 12 | 3 | 8.89 | 4.80 | -0.441 | 0.007 | -0.972 | 0.041 | -0.729 | 0.031 | -0.023 | 0.001 |
| SeSn | oP8 GeS | Sn | Se | -55 | 3.2 | 0.5 | 0 | 24 | 0 | 3.00 | 3.00 | -1.041 | 0.003 | -0.785 | 0.028 | -1.007 | 0.015 | -0.095 | 0.006 |
| SiTa3 | tP32 PTi3 | Ta | Si | -39 | 2.3 | 0.25 | 128 | 72 | 0 | 6.83 | 4.50 | -1.186 | 0.058 | -1.036 | 0.041 | -1.156 | 0.255 | -0.045 | 0.003 |
| SiTa2 | tI12 Al2Cu | Ta | Si | -42 | 2.5 | 0.333 | 44 | 32 | 4 | 7.50 | 5.00 | -1.081 | 0.004 | -0.933 | 0.041 | -1.061 | 0.030 | -0.054 | 0.003 |
| Si3Ta5 | tI32 B3Cr5 | Ta | Si | -42 | 2.5 | 0.375 | 100 | 104 | 4 | 7.60 | 4.67 | -1.066 | 0.028 | -0.999 | 0.041 | -1.050 | 0.085 | -0.017 | 0.001 |
| Si2Ta | hP9 CrSi2 | Ta | Si | -30 | 1.8 | 0.667 | 6 | 30 | 27 | 7.00 | 7.00 | -1.158 | 0.004 | -0.666 | 0.041 | -0.943 | 0.059 | -0.031 | 0.002 |
| SiTi3 | tP32 PTi3 | Ti | Si | -50 | 3.0 | 0.25 | 128 | 72 | 0 | 6.83 | 4.50 | -0.717 | 0.035 | -1.036 | 0.041 | -0.934 | 0.165 | -0.058 | 0.003 |
| Si3Ti5 | hP16 Mn5Si3 | Ti | Si | -73 | 4.3 | 0.375 | 46 | 54 | 6 | 7.30 | 5.50 | -0.672 | 0.005 | -0.848 | 0.041 | -0.788 | 0.026 | -0.028 | 0.002 |
| SiTi | oP8 BFe | Ti | Si | -78 | 4.6 | 0.5 | 20 | 28 | 4 | 8.50 | 4.50 | -0.577 | 0.005 | -1.036 | 0.041 | -0.921 | 0.033 | -0.115 | 0.007 |
| Si2Ti | oF24 Si2Ti | Ti | Si | -58 | 3.5 | 0.667 | 16 | 80 | 40 | 7.00 | 5.00 | -0.700 | 0.005 | -0.933 | 0.041 | -0.877 | 0.045 | -0.060 | 0.004 |
| SiU3 | tI16 SiU3 | U | Si | -21 | 1.3 | 0.25 | 48 | 48 | 0 | 6.00 | 6.00 | -0.921 | 0.014 | -0.777 | 0.041 | -0.867 | 0.043 | -0.018 | 0.001 |
| Si2U3 | tP10 Si2U3 | U | Si | -35 | 2.0 | 0.4 | 32 | 32 | 2 | 8.00 | 4.50 | -0.691 | 0.014 | -1.036 | 0.041 | -0.886 | 0.044 | -0.022 | 0.001 |
| SiU | oP8 BFe | U | Si | -41 | 2.4 | 0.5 | 20 | 28 | 4 | 8.50 | 4.50 | -0.650 | 0.014 | -1.036 | 0.041 | -0.904 | 0.043 | -0.061 | 0.004 |
| Si5U3 | hP3 AlB2 | U | Si | -43 | 2.6 | 0.625 | 4 | 12 | 3 | 8.89 | 4.80 | -0.621 | 0.014 | -0.972 | 0.041 | -0.811 | 0.037 | -0.014 | 0.001 |
| Si2U | hP3 AlB2 | U | Si | -43 | 2.6 | 0.667 | 3 | 12 | 4 | 9.00 | 5.00 | -0.614 | 0.014 | -0.933 | 0.041 | -0.811 | 0.040 | -0.037 | 0.002 |
| Si3U | cP4 AuCu3_1 | U | Si | -33 | 2.0 | 0.75 | 3 | 12 | 12 | 9.00 | 6.00 | -0.614 | 0.014 | -0.777 | 0.041 | -0.724 | 0.074 | -0.029 | 0.002 |
| SiV3 | cP8 Cr3Si | V | Si | -47 | 2.8 | 0.25 | 30 | 24 | 0 | 7.00 | 6.00 | -0.763 | 0.012 | -0.777 | 0.041 | -0.811 | 0.045 | -0.040 | 0.002 |
| Si3V5 | tI32 Si3W5 | V | Si | -54 | 3.2 | 0.375 | 92 | 112 | 4 | 7.40 | 5.00 | -0.722 | 0.012 | -0.933 | 0.041 | -0.847 | 0.036 | -0.020 | 0.001 |
| Si5V6 | oI44 Ge5Ti6 | V | Si | -49 | 2.9 | 0.455 | 88 | 160 | 24 | 7.00 | 5.20 | -0.763 | 0.073 | -0.897 | 0.041 | -0.843 | 0.146 | -0.013 | 0.001 |
| Si2V | hP9 CrSi2 | V | Si | -40 | 2.4 | 0.667 | 6 | 30 | 27 | 7.00 | 7.00 | -0.763 | 0.012 | -0.666 | 0.041 | -0.756 | 0.067 | -0.041 | 0.002 |
| Si3W5 | tI32 Si3W5 | W | Si | -18 | 1.1 | 0.375 | 92 | 112 | 4 | 7.40 | 5.00 | -1.192 | 0.009 | -0.933 | 0.041 | -1.069 | 0.030 | -0.007 | 0.000 |
| Si2W | tI6 MoSi2 | W | Si | -31 | 1.9 | 0.667 | 4 | 20 | 18 | 7.00 | 7.00 | -1.260 | 0.009 | -0.666 | 0.041 | -0.996 | 0.064 | -0.033 | 0.002 |
| Si3Y5 | hP16 Mn5Si3 | Y | Si | -72 | 4.3 | 0.375 | 46 | 54 | 6 | 7.30 | 5.50 | -0.603 | 0.004 | -0.848 | 0.041 | -0.753 | 0.023 | -0.028 | 0.002 |
| Si4Y5 | oP36 Ge4Sm5 | Y | Si | -77 | 4.6 | 0.444 | 88 | 126 | 6 | 7.55 | 4.31 | -0.583 | 0.004 | -1.081 | 0.041 | -0.858 | 0.051 | -0.025 | 0.002 |
| SiY | oC8 BCr | Y | Si | -80 | 4.8 | 0.5 | 20 | 28 | 4 | 8.50 | 4.50 | -0.518 | 0.004 | -1.036 | 0.041 | -0.896 | 0.031 | -0.119 | 0.007 |
| Si5Y3 | tI12 ThSi2 | Y | Si | -74 | 4.4 | 0.625 | 16 | 48 | 12 | 8.89 | 4.80 | -0.495 | 0.004 | -0.972 | 0.041 | -0.757 | 0.028 | -0.024 | 0.001 |
| SiZr3 | tP32 PTi3 | Zr | Si | -66 | 3.9 | 0.25 | 128 | 72 | 0 | 6.83 | 4.50 | -0.925 | 0.045 | -1.036 | 0.041 | -1.057 | 0.226 | -0.077 | 0.005 |
| SiZr2 | tI12 Al2Cu | Zr | Si | -82 | 4.9 | 0.333 | 44 | 32 | 4 | 7.50 | 5.00 | -0.843 | 0.015 | -0.933 | 0.041 | -0.994 | 0.064 | -0.106 | 0.006 |
| Si2Zr3 | tP10 Si2U3 | Zr | Si | -93 | 5.5 | 0.4 | 32 | 32 | 2 | 8.00 | 4.50 | -0.790 | 0.015 | -1.036 | 0.041 | -0.974 | 0.047 | -0.060 | 0.004 |
| SiZr | oP8 BFe | Zr | Si | -87 | 5.2 | 0.5 | 20 | 28 | 4 | 8.50 | 4.50 | -0.744 | 0.015 | -1.036 | 0.041 | -1.019 | 0.048 | -0.129 | 0.008 |
| Si2Zr | oC12 Si2Zr | Zr | Si | -59 | 3.5 | 0.667 | 12 | 40 | 12 | 8.00 | 4.00 | -0.790 | 0.015 | -1.166 | 0.041 | -1.039 | 0.046 | -0.061 | 0.004 |
| SmSn3 | cP4 AuCu3_2 | Sm | Sn | -55 | 3.3 | 0.75 | 0 | 12 | 12 | 6.00 | 6.00 | -0.357 | 0.004 | -0.520 | 0.003 | -0.486 | 0.012 | -0.047 | 0.003 |
| SmTl3 | cP4 AuCu3_2 | Tl | Sm | -40 | 2.4 | 0.25 | 12 | 12 | 0 | 6.00 | 6.00 | -0.315 | 0.001 | -0.357 | 0.004 | -0.370 | 0.006 | -0.034 | 0.002 |
| Sn5Ti6 | hP22 Sn5Ti6 | Sn | Ti | -38 | 2.2 | 0.545 | 26 | 72 | 30 | 6.20 | 5.50 | -0.504 | 0.003 | -0.891 | 0.005 | -0.708 | 0.011 | -0.011 | 0.001 |
| Sn3Ti5 | hP16 Mn5Si3 | Sn | Ti | -39 | 2.3 | 0.625 | 6 | 54 | 46 | 5.50 | 7.30 | -0.568 | 0.003 | -0.672 | 0.005 | -0.635 | 0.013 | -0.015 | 0.001 |
| SnTi2 | hP6 InNi2 | Sn | Ti | -40 | 2.4 | 0.667 | 0 | 22 | 14 | 5.50 | 6.25 | -0.568 | 0.003 | -0.784 | 0.005 | -0.714 | 0.013 | -0.038 | 0.002 |
| SnTi3 | hP8 Ni3Sn | Sn | Ti | -37 | 2.2 | 0.75 | 0 | 24 | 24 | 6.00 | 6.00 | -0.520 | 0.003 | -0.817 | 0.005 | -0.701 | 0.016 | -0.032 | 0.002 |
| Sn3V2 | oF48 CuMg2 | Sn | V | -26 | 1.6 | 0.4 | 176 | 128 | 16 | 8.33 | 4.17 | -0.375 | 0.003 | -1.282 | 0.012 | -0.849 | 0.023 | -0.020 | 0.001 |
| SnV3 | cP8 Cr3Si | Sn | V | -17 | 1.0 | 0.75 | 0 | 24 | 30 | 6.00 | 7.00 | -0.520 | 0.003 | -0.763 | 0.012 | -0.656 | 0.038 | -0.014 | 0.001 |
| Sn3Y | cP4 AuCu3_2 | Sn | Y | -52 | 3.1 | 0.25 | 12 | 12 | 0 | 6.00 | 6.00 | -0.520 | 0.003 | -0.734 | 0.004 | -0.672 | 0.011 | -0.045 | 0.003 |
| Sn2Y | oC12 Si2Zr | Sn | Y | -65 | 3.8 | 0.333 | 12 | 40 | 12 | 4.00 | 8.00 | -0.780 | 0.003 | -0.550 | 0.004 | -0.732 | 0.011 | -0.067 | 0.004 |
| Sn3Y5 | hP16 Mn5Si3 | Sn | Y | -88 | 5.2 | 0.625 | 6 | 54 | 46 | 5.50 | 7.30 | -0.568 | 0.003 | -0.603 | 0.004 | -0.619 | 0.011 | -0.034 | 0.002 |
| Sn3Yb | cP4 AuCu3_2 | Yb | Sn | -45 | 2.7 | 0.75 | 0 | 12 | 12 | 6.00 | 6.00 | -0.269 | 0.004 | -0.520 | 0.003 | -0.433 | 0.011 | -0.039 | 0.002 |
| Sn2Zr | oF24 Si2Ti | Sn | Zr | -67 | 4.0 | 0.333 | 40 | 80 | 16 | 5.00 | 7.00 | -0.624 | 0.003 | -0.903 | 0.015 | -0.834 | 0.020 | -0.070 | 0.004 |
| Sn3Zr5 | hP16 Mn5Si3 | Sn | Zr | -85 | 5.1 | 0.625 | 6 | 54 | 46 | 5.50 | 7.30 | -0.568 | 0.003 | -0.866 | 0.015 | -0.750 | 0.032 | -0.033 | 0.002 |
| SnZr4 | cP8 Cr3Si | Sn | Zr | -59 | 3.5 | 0.8 | 0 | 24 | 30 | 7.50 | 6.56 | -0.416 | 0.003 | -0.963 | 0.015 | -0.731 | 0.045 | -0.041 | 0.002 |
| TaV2 | cF24 Cu2Mg | V | Ta | -1 | 0.1 | 0.333 | 48 | 96 | 16 | 6.00 | 8.00 | -0.890 | 0.012 | -1.013 | 0.004 | -0.953 | 0.023 | -0.001 | 0.000 |
| TbTl3 | cP4 AuCu3_2 | Tl | Tb | -36 | 2.1 | 0.25 | 12 | 12 | 0 | 6.00 | 6.00 | -0.315 | 0.001 | -0.671 | 0.004 | -0.524 | 0.005 | -0.031 | 0.002 |
| TeZn | cF8 SZn | Zn | Te | -60 | 3.6 | 0.5 | 0 | 16 | 0 | 2.00 | 2.00 | -0.676 | 0.001 | -1.019 | 0.015 | -1.003 | 0.016 | -0.155 | 0.009 |
| Tl3Yb | cP4 AuCu3_2 | Yb | Tl | -37 | 2.2 | 0.75 | 0 | 12 | 12 | 6.00 | 6.00 | -0.269 | 0.004 | -0.315 | 0.001 | -0.324 | 0.005 | -0.032 | 0.002 |
| V2Zr | cF24 Cu2Mg | V | Zr | -1 | 0.1 | 0.333 | 48 | 96 | 16 | 6.00 | 8.00 | -0.890 | 0.012 | -0.790 | 0.015 | -0.841 | 0.029 | -0.001 | 0.000 |
| W2Zr | cF24 Cu2Mg | Zr | W | -3 | 0.2 | 0.667 | 16 | 96 | 48 | 8.00 | 6.00 | -0.790 | 0.015 | -1.470 | 0.009 | -1.133 | 0.025 | -0.002 | 0.000 |

**References**

[1] D.B. Miracle, G.B. Wilks, A.G. Dahlman, J.E. Dahlman, The strength of chemical bonds in solids and liquids, Acta Mater. 59(20) (2011) 7840-7854. <https://doi.org/10.1016/j.actamat.2011.09.003>
